# Comparative study on chromatin loop callers using Hi-C data reveals their effectiveness

**DOI:** 10.1186/s12859-024-05713-w

**Published:** 2024-03-21

**Authors:** H. M. A. Mohit Chowdhury, Terrance Boult, Oluwatosin Oluwadare

**Affiliations:** 1 Department of Computer Science, University of Colorado at Colorado Springs, 1420 Austin Bluffs Pkwy, Colorado Springs, CO 80918 USA; 2 Department of Biomedical Informatics, University of Colorado Anschutz Medical Campus, Aurora, CO USA

**Keywords:** Chromosome, Chromatin, Loop, DNA, Hi-C, Machine learning, Computer vision, Clustering, Probability, Classification

## Abstract

**Background:**

Chromosome is one of the most fundamental part of cell biology where DNA holds the hierarchical information. DNA compacts its size by forming loops, and these regions house various protein particles, including CTCF, SMC3, H3 histone. Numerous sequencing methods, such as Hi-C, ChIP-seq, and Micro-C, have been developed to investigate these properties. Utilizing these data, scientists have developed a variety of loop prediction techniques that have greatly improved their methods for characterizing loop prediction and related aspects.

**Results:**

In this study, we categorized 22 loop calling methods and conducted a comprehensive study of 11 of them. Additionally, we have provided detailed insights into the methodologies underlying these algorithms for loop detection, categorizing them into five distinct groups based on their fundamental approaches. Furthermore, we have included critical information such as resolution, input and output formats, and parameters. For this analysis, we utilized the GM12878 Hi-C datasets at 5 KB, 10 KB, 100 KB and 250 KB resolutions. Our evaluation criteria encompassed various factors, including memory usages, running time, sequencing depth, and recovery of protein-specific sites such as CTCF, H3K27ac, and RNAPII.

**Conclusion:**

This analysis offers insights into the loop detection processes of each method, along with the strengths and weaknesses of each, enabling readers to effectively choose suitable methods for their datasets. We evaluate the capabilities of these tools and introduce a novel Biological, Consistency, and Computational robustness score ($BCC_{score}$) to measure their overall robustness ensuring a comprehensive evaluation of their performance.

## Background

**Fig. 1:**
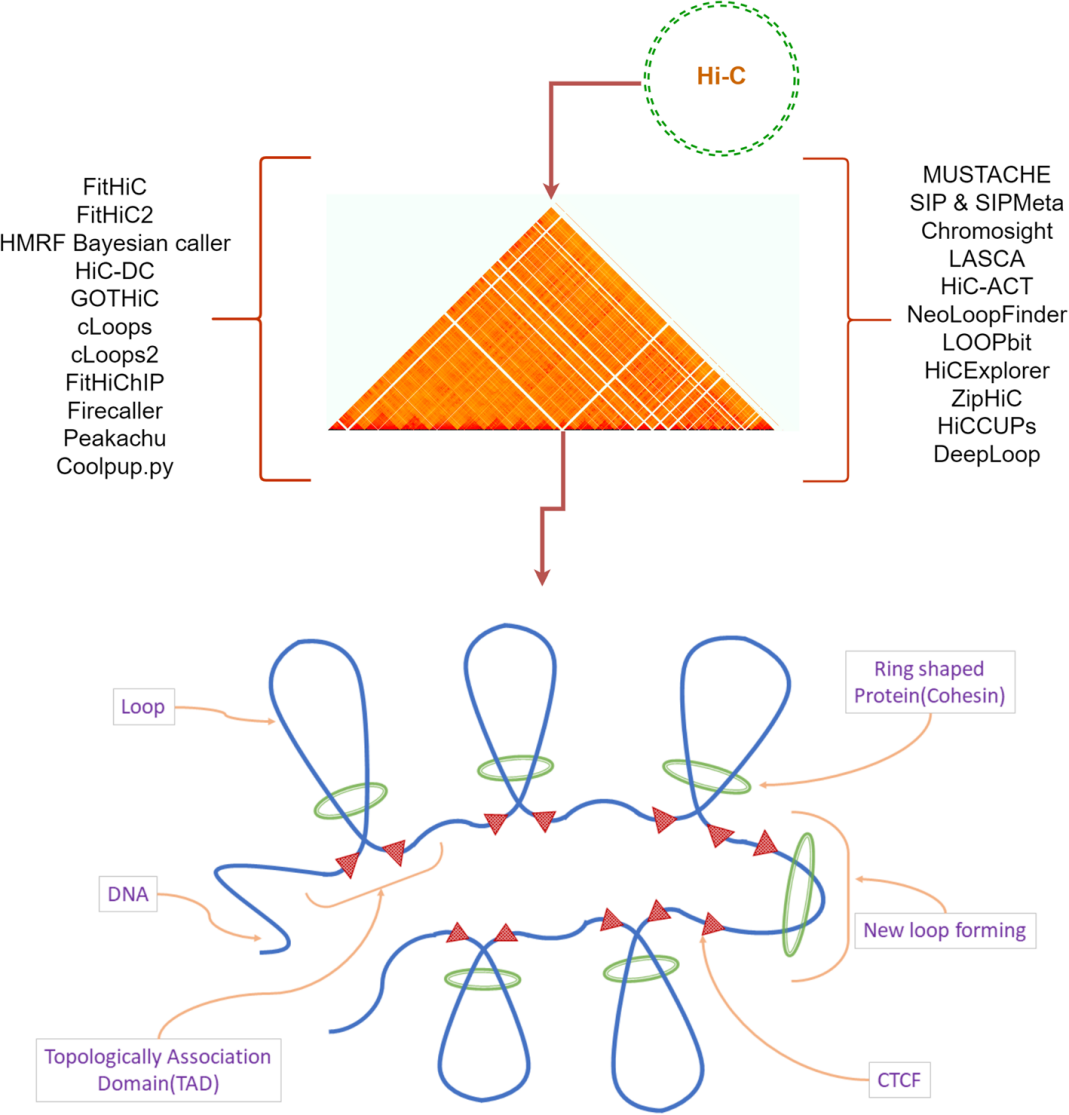
A brief overview of chromatin loops. The green-colored ring-shaped protein first pulls DNA through it creating a loop. CTCF as a binder or lock for this ring and widely known as CCCTC binding or transcription factor. TADs [11] are formed by the folding of chromatin, which is a complex of DNA, RNA, and proteins. The ring-shaped protein, which tightens the loops is called the cohesin [65]

DNA and chromosomes hold the most important information about a species. Scientists have been working to reveal the internal structure of chromosomes and DNA to answer questions about intra-chromosomal interaction, hierarchical properties, and DNA segments [1, 2]. Regulatory information is also important to solve real-life problems such as disease prediction and analysis [3]. Studies have revealed that each chromosome is positioned in a specific region known as a *chromosome territory* [4], characterized by a specific pattern. In the nucleus (Fig. 1), a ring-shaped cohesin protein pulls DNA through its center to create a loop and is bounded by CTCF (called extrusion barrier) [5, 6]. This loop results in the 3D structure of DNA in a small region inside the chromatin. Peaks are areas enriched in aligned reads due to protein binding from ChIP-sequencing or MDIP-sequencing [7, 8]. These loops and peaks are important regions from which we can answer questions about chromatin interaction and conformation [6, 9]. Various proteins have been found in these regions, such as cohesin, CTCF, and some H3 protein markers like H3K27ac and H3K27me3 [5, 10]. Scientists have also observed that Topologically Associating Domains (TADs) around these loop regions are crucial for chromosome interaction [2, 11].

**Fig. 2:**
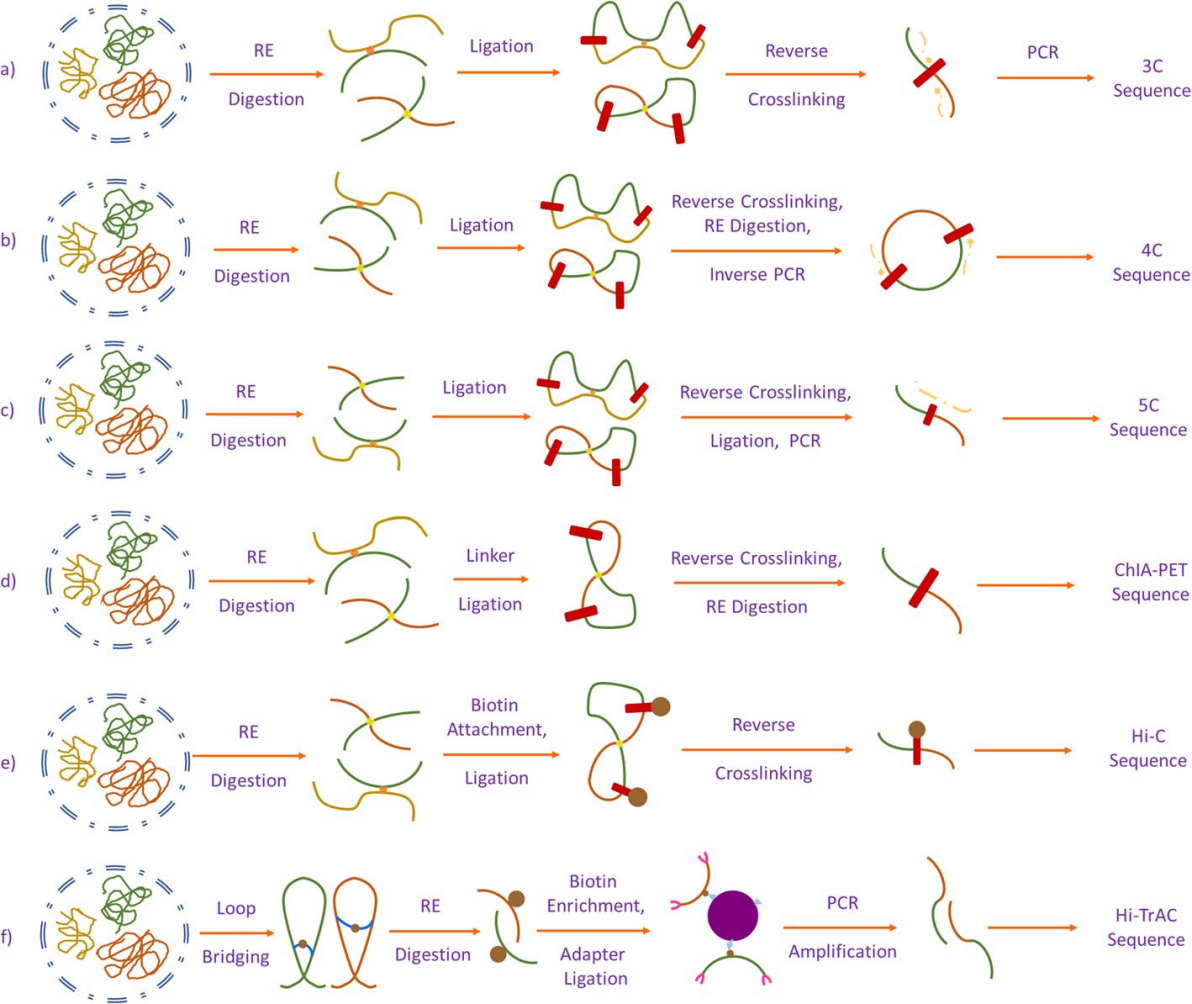
A brief overview of sequencing techniques. Chromosome conformation capture technologies: **a** Chromosome spatial organization analysis in 3C technology starts with cell population crosslinking and fragmentation with a restriction enzyme. Next, it goes through intramolecular ligation and reverse-crosslinking and performs semi-quantitative or quantitative PCR. **b** Chromosome spatial organization analysis in 4C technology starts with cell population crosslinking and fragmentation with restriction enzyme. Next, it goes through intramolecular ligation and reverse crosslinking. Next, it goes through digestion with a restriction enzyme and ligation, and finally, applies inverse PCR. **c** Chromosome spatial organization analysis in 5C technology starts with cell population crosslinking and fragmentation with a restriction enzyme. Next, it goes through intramolecular ligation and reverse-crosslinking and performs synthetic ligation and multiplex PCR. **d** Chromosome spatial organization analysis in ChIA-PET technology starts with cell population crosslinking and fragmentation with a restriction enzyme. Then, DNA linker ligation attracts nucleotides and performs reverse crosslinking and PCR. **e** Chromosome spatial organization analysis in Hi-C technology starts with cell population crosslinking and fragmentation with restriction enzyme. Then, it attaches biotin-labeled nucleotide, and goes through blunt ligation and PCR [1, 2, 10, 13, 17, 66, 67]. **f** First, HiC-TrAC creates a bridge on chromatin loops and splices DNA with restriction enzymes. Then, the process is fertilized with streptavidin beads. Finally, DNA fragments having a biotin label attach with a multiplexing adapter and go through a PCR amplification [9]

The evolution in C-technology was initiated by Dekker et al. when they expanded the Chromatin Conformation Capture (3C) method [1]. Subsequently, other 3C-based methods (Hi-C [12], ChIA-PET [13–15], Hi-TrAC [16]) were developed sharing some common methodology briefly presented in Fig. 2. Hi-C, a combination of 3C and next-generation sequencing techniques, represents a significant advance in genome analysis. One of its main advantages is that it is not subject to a set of any primers [17–19]. It is an unbiased and unsupervised method [2, 12] for genome analysis, generating genome-wide contact maps [17]. It is widely used for analyzing genomic organizational principles, chromosome structure at the mitotic stage, and anatomical changes in human disease [2, 20–22]. The advent of 3C technology [17] and Hi-C technology [18] has propelled gene analysis in various directions and has influenced the development of numerous loop and peak calling techniques [23–42]. Although their primary aim is to identify loops and peaks, these methods offer a secondary advantage by providing information about gene regulation, such as interaction, structure, and protein reactions [16, 30, 42]. The development of machine learning algorithms has propelled 3D genome spatial architecture analyses into a new dimension [43–47]. Specifically in the loop detection domain, Scientists have developed different tools to predict loop regions, employing various machine learning-based approaches such as computer vision and classification based methods. Mustache [40], Chromosight [42], SIP & SIPMeta [41] have demonstrated the application of computer vision algorithms to predict loop regions, marking a new era in genomic analysis with many other tools.

In this manuscript, we present a comprehensive analysis of eleven loop detection tools based on Hi-C datasets. We evaluate how these tools perform in predicting loops, their recovery of biological features such as H3K27ac, RNAPII, and CTCF, the impact of sequencing depth, and discuss their strengths and weaknesses. Our analysis goes beyond theory, giving practical insights into how these tools can be used, including the necessary technical details and parameters. By merging these aspects, we identify overlaps, uncovering connections, computational efficiency, similarities and results consistency in the studied techniques. To quantitatively measure the capabilities of these tools across these analysis categories, we created a novel aggregated score called the $BCC_{score}$ to measure their overall robustness ensuring a comprehensive evaluation of their performance.

## Results

We used GM12878 [48] (Human Lymphoblastoid) primary full genome Hi-C dataset at 5 KB, 10 KB, 100 KB and 250 KB, and for specific case study, chromosome 1 and 6 replicate and Knight–Ruiz (KR) normalized Hi-C dataset at 5 KB and 10 KB for our analysis. We prepared input data using HiCExplorer (cool), and sam and bam (bed and bedpe) tools. All methods were analyzed with their input and output details, and we presented their loop count across different resolutions. For assessments, we evaluated their overlap, peak and Aggregate Peak Analysis (APA) score, biological feature recovery (CTCF, H3K27ac, and RNAPII), the recovery performance and efficiency across sequencing depths, tools running time and memory usages dividing them into two subsections: Computational Analysis and Biological Validation in the following sections.

### Computational analysis

This section is dedicated to analyzing results directly generated from the execution of loop detection algorithms. These analyses include the comparative analysis of the tools’ results across different resolutions, normalization algorithm, their overlap and reproducibility, the peak and APA analysis, running time and memory consumption. The conducted analysis aims to assess the comparative performance of these tools in terms of loop predictions and computational consistency. It is important to note that the primary objective of this section is to present, evaluate and compare the computational aspects of each tool and not to demonstrate biological accuracy or validity.

#### Loop detection within different resolutions and normalization

**Fig. 3:**
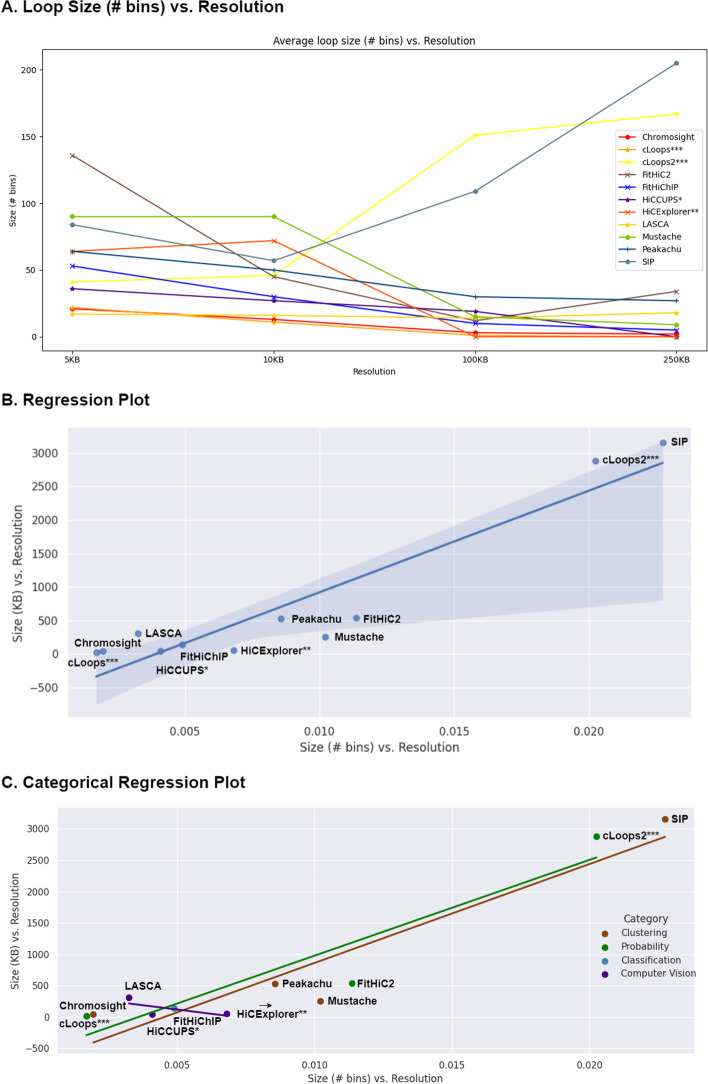
**A** Illustration of chromatin loop size in terms of number of mean bins. Regression plots of chromatin loop caller tools calculating the slope of resolution and loop size (KB) versus loop size (# bins) and resolution where **B** overall regression and **C** categorical regression plot. HiCCUPS* and HiCExplorer** didn’t produce results at low resolutions and cLoops*** and cLoops2*** do not have resolution parameter

We successfully executed 11 out of 22 methods in our analysis, presenting their loop prediction capabilities (Fig. 3). The remaining 11 methods couldn’t be executed due to computational issues, with some failing to produce results or encountering errors during execution: ZipHiC [34] does not provide a clear instruction to run their script, HMRF Bayesian caller [36] has no public source code repository, LOOPBit [23], Coolpup.py [39], DeepLoop [49] and FIREcaller [37] errored out during analysis with no results, HiC-ACT [28] and GOTHiC [32] did not produce any output upon execution, and we couldn’t access a R library for HiC-DC [33] or access its installation instruction.

In Table 1, our analysis shows that FitHiC2 predicts a higher loop count, suggesting probable chromosomal contacts, while cLoops predicts the fewest loops. Additionally, FitHiChIP, Mustache, and LASCA predict a significant number of loops. Most tools tend to predict more loops at 5 KB and 10 KB (high) resolutions compared to 100 KB and 250 KB (low) resolutions. Notably, cLoops2, HiCCUPS, and Mustache predict a higher loop count at 10 KB resolution, whereas other tools show more loops at 5 KB resolution. Interestingly, the loop count detected by Chromosight, LASCA, Mustache, Peakachu, and SIP significantly decreases at lower resolutions. HiCExplorer and HiCCUPS do not generate results at lower resolutions; their minimum resolutions are 10 KB and 25 KB, respectively [27, 48]. Though cLoops and cLoops2 does not provide any resolution parameter, we changed the eps, and cLoops produced same number of loop counts at different eps and cLoops2 produced different results.

**Table 1:** Loop count of primary GM12878 at 5 KB, 10 KB, 100 KB and 250 KB resolution

Tools	5 KB	10 KB	100 KB	250 KB	Average
Chromosight	15402	10542	562	85	6648
cLoops***	763	763	763	763	763
cLoops2***	18634	23986	282	2	10726
FitHiC2	463587	448482	378871	332268	405802
FitHiChIP	8554	39239	62101	21139	32758
HiCCUPS*	33196	41471	0	0	37334
HiCExplorer**	26895	23212	0	0	25054
LASCA	64975	33580	4876	1886	26329
Mustache	20113	68703	18277	266	26840
Peakachu	49668	28985	137	12	19701
SIP	8765	4114	167	383	3357

HiCCUPS* and HiCExplorer** didn’t produce results at low resolutions and cLoops*** and cLoops2*** do not have a resolution parameter

We compared loop sizes (Fig. 3A) at 5 KB, 10 KB, 100 KB and 250 KB resolution using GM12878 primary dataset, revealing a trend across different sequencing depth. For most loop callers, the average number of bins in the loop decreases with resolution decrease (from high to low resolution). Conversely, the average loop sizes demonstrated an opposite trend, increased with the resolution increase. Only cLoops2 and SIP exhibited an increase in size (# of bins) with the resolution decrease. A linear regression plot (Fig. 3B) demonstrated that most of them fell within the regression boundary except cLoops2, LASCA, HiCExplorer and Mustache. The regression category-wise plot further elucidated individual category information (Fig. 3C). Additional file 1: Figs. S1 and S2 shows the chromatin loop size and regression plots for GM12878 primary, replicate and KR normalized dataset for chromosome 1 and 6. Further individual analyses of each tool are presented in the subsequent paragraphs, comparing results related to loop counts and input parameter robustness.

LASCA [24] implements Weibull distribution mechanism for loop detection and enhancer-promoter interaction using Hi-C data across different types of organisms. Though they do not provide any command line facility to run, we used LASCA to identify loops at high and low resolution importing LASCA as a Python library. While analyzing LASCA, we counted 26329 average loop count at 5 KB, 10 KB 100 KB and 250 KB resolution using primary GM12878 cell line. LASCA predicts more loops at 5 KB resolution compared to other resolutions. We used replicate and normalized data (chromosome 1 and 6 at 5 KB and 10 KB) and found more loops at 5 KB resolution compared to 10 KB (Additional file 1: Tables S7 and S8).

HiCExplorer provides a robust toolset (such as normalization, data conversion, loop prediction) for chromosomal data analysis and performs well with high-resolution data. HiCExplorer provides an option to set user-specific *p* value and threads, threads per chromosome. We used default setting and got 25053 (average) loop count at 5 KB and 10 KB resolution, we also recorded the result obtained from the normalized and replicate data in Additional file 1: Tables S7 and S8. HiCExplorer does not produce results at low resolution (100 KB and 250 KB), and mentioned in their work that their algorithm accepts only 5 KB and 10 KB resolution data [27]. HiCExplorer also detects protein binding sites that correlate with detected loops and they used different types of dataset for their analysis such as ChIA-PET, HiChIP along with Hi-C.

FitHiC mainly identifies mid-range intra-chromosomal contacts considering the looping effect and biases and finds high-confidence contacts in insulator and heterochromatin regions. FitHiC2 is an updated version of FitHiC where they minimized the mid-range intra-chromosomal contact analysis limitation. They introduced genome-wide contacts analysis in high resolution without sacrificing significant loops. FitHiC2 can analyze data at a specific resolution and has an option to specify the intra-chromosome or inter-chromosome analysis. It requires input files from other analysis tools such as HiCPro and they provided all the scripts for getting these inputs. FitHiC2 produces outputs of significant interaction contact and indirectly we can infer loops at that region. Here we used FitHiC2 in our analysis and it produces 405802 contacts for GM12878 primary data at 5 KB, 10 KB, 100 KB and 250 KB resolution filtering out with $FRD = \frac{1}{\text {total count}}$ as they suggested in their manuscript. While running their repository, we encountered a Python error which is also fixed in our fork repository and we uploaded a docker image for further analysis.

FitHiChIP [31] is mainly focused on HiChIP/PLAC-seq data where they analyzed non-uniform coverage by scaling contact counts which ultimately produces loops even at 2.5 KB resolution. This tool is a versatile tool providing differential loop analysis option. During our analysis, FitHiChIP produces 32758 contact loops from primary GM12878 at different (5 KB, 10 KB, 100 KB and 250 KB) resolution using $1e^{-6}$ threshold of *p* value as they suggested. FitHiChIP accepts HiCPro valid pair files, bin interval and contact matrix, bed, cool, and hic formatted files. It requires a configuration file where we can pass all the settings. In our analysis, we used chromosome-wise cool files and considered peak-to-all interaction analysis using coverage bias correction setting. Though it does not support chromosome-wise analysis, it has a parameter for passing the bin size where user can specify their intended resolution in full form.

Peakachu [38] is a Random Forest classifier and provides pre-build models for different combinations of intra reads and high confidence for different types of datasets such as Hi-C, Micro-C, HiChIP, etc. They accept specific chromosome numbers and resolutions which facilitate the user to analyze as needed. Though it provides balancing parameter for using ICE or KR matrix, it did not accept any specific parameter. In our analysis, we used KR normalization data from HiCExplorer along with primary data. From our primary data analysis, we got 19700 interactions using q-value < 1e−5 on average for the whole genome. In addition to specific chromosome analysis, they have the option to analyze the whole genome. Peakachu can recover most of the loops from protein-centric datasets such as ChIP and ChIA-PET, and they also showed short-range interaction recovery in their analysis result.

Mustache [40] utilized the scale-space theory of computer vision to detect chromatin loops at different sequencing depths of Hi-C and Micro-C data. Mustache provides normalization techniques for users for hic, cool and bias files for text-based contact map along with process, thread, threshold (*p* value), resolution, and chromosome-wise analysis. Mustache detects 26840 loops on average at 5 KB, 10 KB, 100 KB and 250 KB resolutions in our analysis. Mustache can analyze chromosomes at 1 KB resolution Micro-C and 5 KB resolution Hi-C data.

Chromosight [42] implemented pattern recognition technique to detect loops. From our analysis, Chromosight detects 6648 at 5 KB, 10 KB, 100 KB and 250 KB resolutions on average, and it predicts more loops at 5 KB resolution compared to 10 KB, 100 KB and 250 KB resolution, even using replicate and normalized dataset (Additional file 1: Tables S6, S7 and S8). It can identify borders, centromeres, etc and accepts thread parameter. Chromosight analyzes the whole genome and it does not have any parameter for specific resolution. We provided a specific chromosome contact map at a specific resolution in our analysis. It provides three normalizations (auto, raw, and forced) from the user and has inter chromosomal analysis option.

SIP [41] developed to identify missing loops from previous loop callers considering the noise and sequencing depth. SIP can detect more loops at 5 KB resolution compared to 10 KB, 100 KB and 250 KB resolution. Overall, SIP identified 3357 loops on average using GM12878 cell. SIP provides UI for users flexibility. It accepts resolution, CPUs, normalization (VC, VC_SQRT, and KR), FDR, and threshold value parameters. In our analysis, we used cool files, but it support hic and processed files as input. It can analyze deeply sequenced genomes even at 1 KB resolution.

cLoops [25] and cLoops2 [26] are DBSCAN based loop detection algorithm. cLoops calculates the distance between two neighbors describing the distance between two neighbors and analyzes pair-end tags without considering resolution to identify loops with *O*(*Nlog*(*N*)) running time in addition to parallel computation. They provides different analysis plot scripts (heatmap, data quality plots) and chromosome-wise analysis. cLoops2 is the updated version of cLoops with an optimized DBSCAN clustering algorithm with running time *O*(*N*) and provides loop and peak calling algorithm in different ways along with differential loop and domain calling. cLoops2 was developed for detecting loops on Hi-TrAC/TrAC looping data. It can still be used for loop detection for ChIA-PET and HiChIP data like cLoops. cLoops2 provides chromosome-specific analysis but we cannot provide specific resolution to it. Though their loop detection is comparably close to other methods, cLoops2 (10726) predicts more loops compared to cLoops (768) in our analysis (Additional file 1: Tables S6, S7 and S8). Like cLoops, cLoops2 provides chromosome-wise genomic analysis regardless of any resolution. cLoops2 has analysis scripts such as aggregated peaks, domains, etc. cLoops and cLoops2 do not provide any normalization parameter in their command line. Users can specify the number of CPUs to be used in cLoops2 and it has a data conversion tool to other formats.

HiCCUPS [48] provides different versions to run on CPU and GPU. They provide in-tool normalization techniques along with a specific chromosome and resolution. Users can specify thread, threshold value (FDR), and merging distance. During our analysis, we used default parameters, and on average, it predicts 37333 loops at 5 KB and 10 KB and predicts the maximum number of loops at 10 KB resolution rather than 5 KB resolution even using replicate and normalized dataset (Additional file 1: Tables S7 and S8). We limited the highest resolution for HiCCUPS to 25 KB resolution as we could not generate a result beyond this resolution.

#### Overlap and reproducibility

**Fig. 4:**
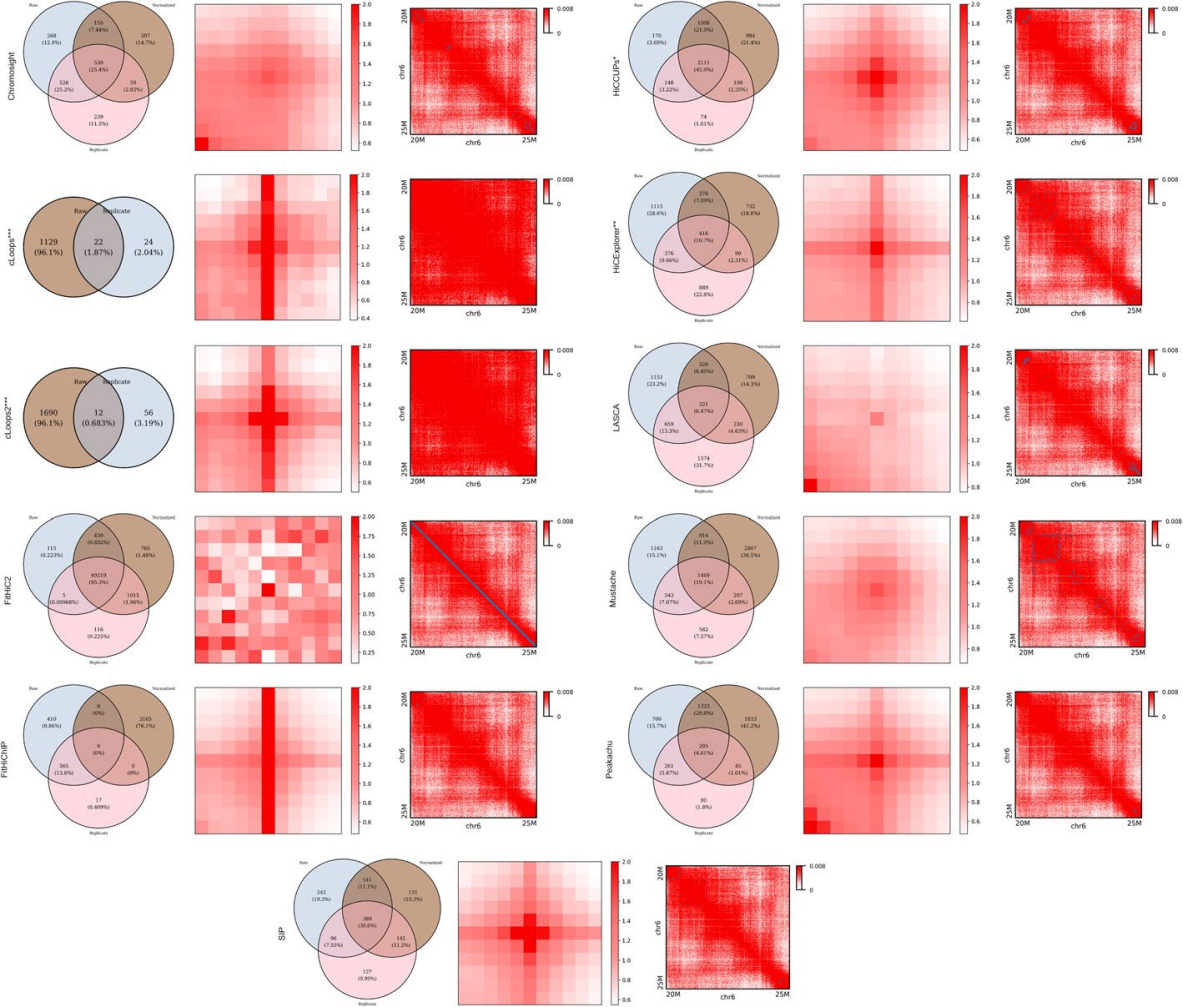
Overlap, APA, and Peak plots (left to right) using primary GM12878 (chromosome 6 at 5 KB). FitHiC2 overlaps 95.3% across the analysis. Apart from that, HiCCUPS has the highest amount (45.9%) of overlap across three different datasets and FitHiChIP does not overlap any loops. SIP (30.6%), Mustache (19.1%), and Chromosight (25.4%) have a significant amount of overlap. cLoops and cLoops2 produce results only for primary and replicate data and the overlap percentage is around 1. FitHiC2 shows enrichment in different regions and has the strong enrichment compared with other tools. cLoops, cLoops2, and FitHiChIP have enrichment in the middle vertical region and they are almost in the same shape. HiCCUPS, SIP, Peakachu, and HiCExplorer have enrichment in the middle region. Peakachu, LASCA, and Chromosight have enrichment in the left lower corner from the center and Mustache shows enrichment in the center pixel. Though FitHiC2 creates a diagonal dark straight line marking peaks, Mustache and HiCExplorer mark the highest number of peaks. Chromosight, LASCA, and SIP mark peaks in the upper left and lower right corners near 20 M and 25 M region. HiCCUPS* and HiCExplorer** didn’t produce results at low resolutions and cLoops*** and cLoops2*** do not have a resolution parameter

In this study, we recognize the significance of comparing Raw and Normalized experiments, in addition to Replicate datasets, for a comprehensive evaluation of the effects of normalization and replication on loop detection tools. Our primary goal is to assess the computational consistency or compromise of loop identification under normalization and to discern differences observed with replicate data sourced from the same dataset. For empirical analysis, we executed all the methods using the GM12878 dataset to observe the overlap (Fig. 4-left). For ease of comparison, we divided our dataset into various combinations, involving chromosomes 1 and 6 at 5 KB and 10 KB resolution. Notably, cLoops and cLoops2 accept bedpe files and do not allow any normalization parameter, leading our analysis without normalization data. For the 5 KB data on chromosome 6, FitHiC2 exhibited a 95% overlap among primary, replicate, and KR normalized data, indicating high reproducibility. In contrast, cLoops, cLoops2, and FitHiChIP displayed the lowest overlap, nearly 0%. On average, Chromosight, HiCCUPS, and SIP exhibited 25% - 46% overlap. Chromosight showed more reproducibility between primary and replicate datasets, while SIP displayed more reproducibility between normalized and replicate data. Chromosight, FitHiC2, and HiCCUPS demonstrated higher reproducibility rates across our dataset combinations.

For further analysis, we conducted the same assessment on chromosome 1 at 5 KB and 10 KB, and chromosome 6 at 10 KB (Additional file 1: Figs. S3, S4 and S5). FitHiC2 consistently showed overlaps above 90%, reaching almost 100% for chromosome 6 at 10 KB. In contrast, FitHiChIP exhibited no overlap, while cLoops and cLoops2 showed around 1% overlap. Chromosight and Mustache displayed an opposite trend for chromosome 1 and 6, increasing for chromosome 1 at 5 KB and decreasing for chromosome 6 at 5 KB. HiCCUPS consistently demonstrated overlaps of over 45% on 10 KB resolution. Peakachu showed significant overlap on chromosome 6 at 10 KB, ranging from 4.61% to 50.6%, and HiCExplorer, LASCA, and SIP consistently displayed overlaps ranging from 8% to 40% throughout the analysis.

#### Running time and memory consumption on different resolution within different methods

We compared the average running time and memory consumption (Fig. 12) of every individual tool to further assess their robustness. We ran all our tools on an Ubuntu Server operating on Intel Xeon E7-4870 @ 2.40GHz with 160 cores and 1038GB memory. Although each tool accepts different types of parameters, such as threads, chromosomes, and resolution, we attempted to compare them on the same scale using their default settings. The average running time and memory consumption on our server is calculated and the detailed running times and memory consumption are provided in Additional file 1: Tables S1, S2, S3, S4 and S5. We observed that Chromosight took the least amount of time, while cLoops2 took the highest amount of time. In comparison, SIP, HiCCUPS, Peakachu, Chromosight, HiCExplorer and Mustache ran within a shorter period, while LASCA, FitHiC2, FitHiChIP and cLoops ran within comparable time range (Fig. 12A). While analyzing memory consumption, we observed that FitHiC2 consumed least amount of memory while Chromosight used the highest amount of memory. On average, cLoops2, FitHiChIP, Mustache and Peakachu used a satisfactory amount of memory compared to other tools (Fig. 12B).

#### Peak and APA analysis

Peaks represent regions with the highest observed interactions, contributing to the formation of loops within chromatin. In our analysis, we focused on the 20–25 M region to visualize peaks using loop lists from various tools. Fig. 4-right displays peaks on chromosome 6 at 5 KB resolution for primary GM12878 data. FitHiC2, Mustache, and HiCExplorer exhibit the highest number of peaks in this specific region. Chromosight, LASCA, and SIP mark peaks in the upper left and lower right corners near the 20 M and 25 M regions. FitHiC2 marks the highest number of peaks, forming a diagonal straight line for every dataset combination. We analyzed peak regions for chromosome 1 at 5 KB and 10 KB, and chromosome 6 at 10 KB resolution (Additional file 1: Figs. S6, S7, S8, S9, S10, S11, S12, S13, S14, S15 and S16). For chromosome 1 at 5 KB and 10 KB, all tools preserve almost the same peaks, except for Mustache. For primary and replicate data, Mustache marks more peaks at 5 KB compared to 10 KB, which is the opposite for KR normalized data.

To quantify the loop prediction, we conducted aggregated peak analysis (APA) across the results. APA measures the Hi-C signal enrichment over an entire peak list, providing insights into the quality of loop lists, especially at lower resolutions. Submatrices are calculated from the Hi-C contact map file, and the sum of these submatrices produces an APA matrix. An APA score greater than 1 indicates enrichment, with darker colors in the heatmap indicating higher enrichment. Figure 4-middle shows APA plots for chromosome 6 at 5 KB resolution using GM12878 primary data. FitHiC2 shows strong enrichment at different location with APA score 28.1 and cLoops (5.12), cLoops2 (3.98), FitHiChIP (4.89), and SIP (3.7) exhibit the highest enrichment in the center of the plots. Except LASCA ($<1$), all the tools show enrichment greater than one (Additional file 1: Table S19). Furthermore, we performed APA analysis for chromosome 1 (5 KB and 10 KB) and chromosome 6 (10 KB) using primary, KR normalized, and replicated GM12878 data (Additional file 1: Figs. S17, S18, S19, S20, S21, S22, S23, S24, S25, S26 and S27). For normalized data, using chromosome 6 at 5 KB, all tools scored greater than one and FitHiC2 (36.9) has the highest score (Additional file 1: Table S10). HiCCUPS (2.12), SIP (2.72), Mustache (1.52), Chromosight (1.19), and Peakachu (1.26) show gradual enrichment around the center. For 10 KB data, SIP (2.77) produces a stronger central pixel color compared to 5 KB resolution data, whereas other tools produce prominent plots at 5 KB resolution. HiCExplorer produces almost identical visual plots for primary and replicate, KR normalized plots, and they are highly enriched at the central pixel of heatmaps. Throughout the analysis, FitHiC2 (83.73) shows strong and void enrichment at different focal points with robust enrichment at various locations (Additional file 1: Tables S9, S10 and S11).

### Biological validation

The analysis conducted in this section serves to validate the computational results produced by each of the loop prediction algorithms. The assessments performed here include: evaluation of CTCF, RNAPII and H3k27ac recovery rate, recovery efficiency, and recovery performance across sequencing depth. These analyses aims to demonstrate both the biological validity of detected loops and the robustness of the tools’ results under varying depth of reads coverage in an Hi-C experiment.

#### CTCF, H3K27ac and RNAPII recovery

**Fig. 5:**
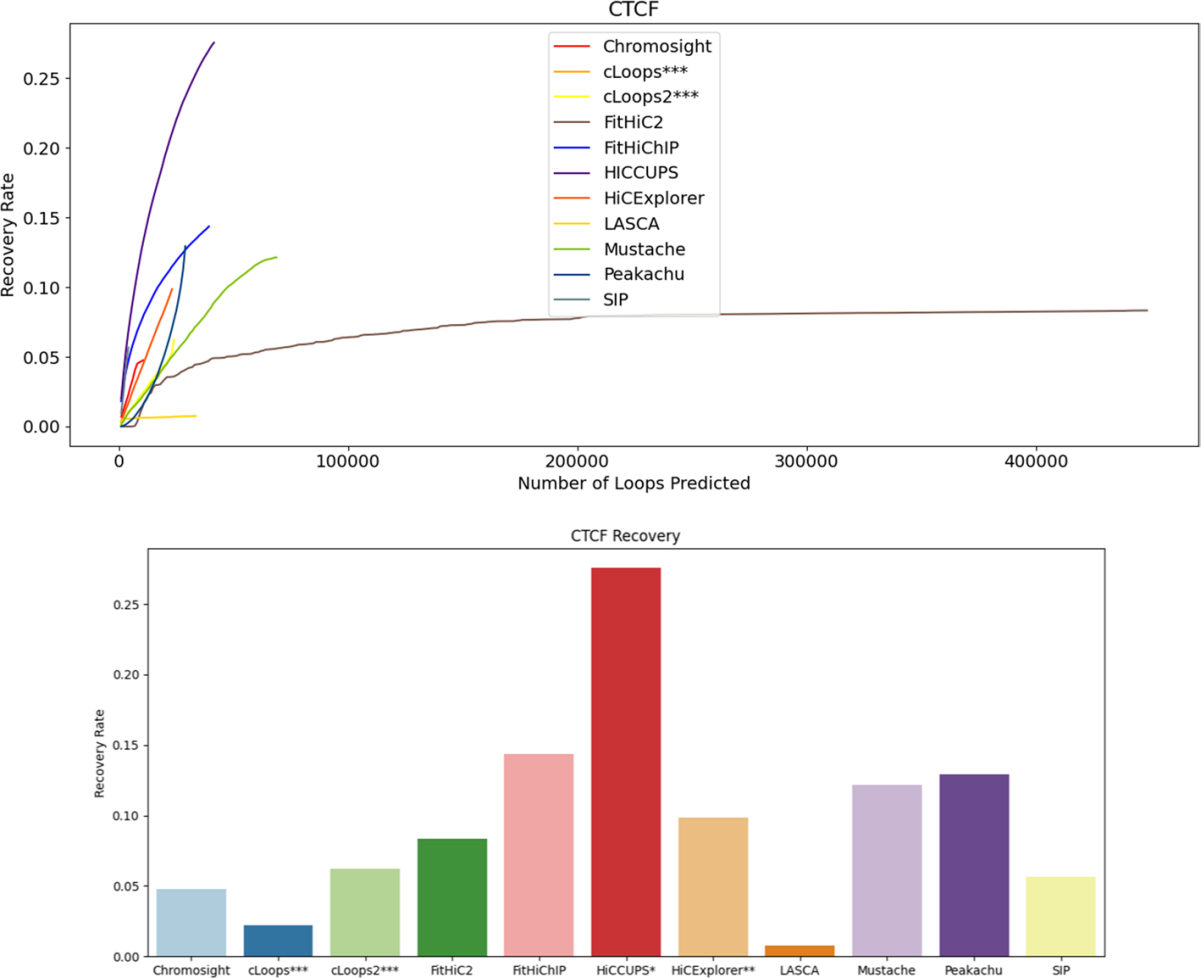
CTCF recovery rate using primary GM12878 at 10 KB. HiCCUPS recovers most of the CTCF, and LASCA recovers the least fraction of CTCF. HiCCUPS* and HiCExplorer** didn’t produce results at low resolutions and cLoops*** and cLoops2*** do not have a resolution parameter

**Fig. 6:**
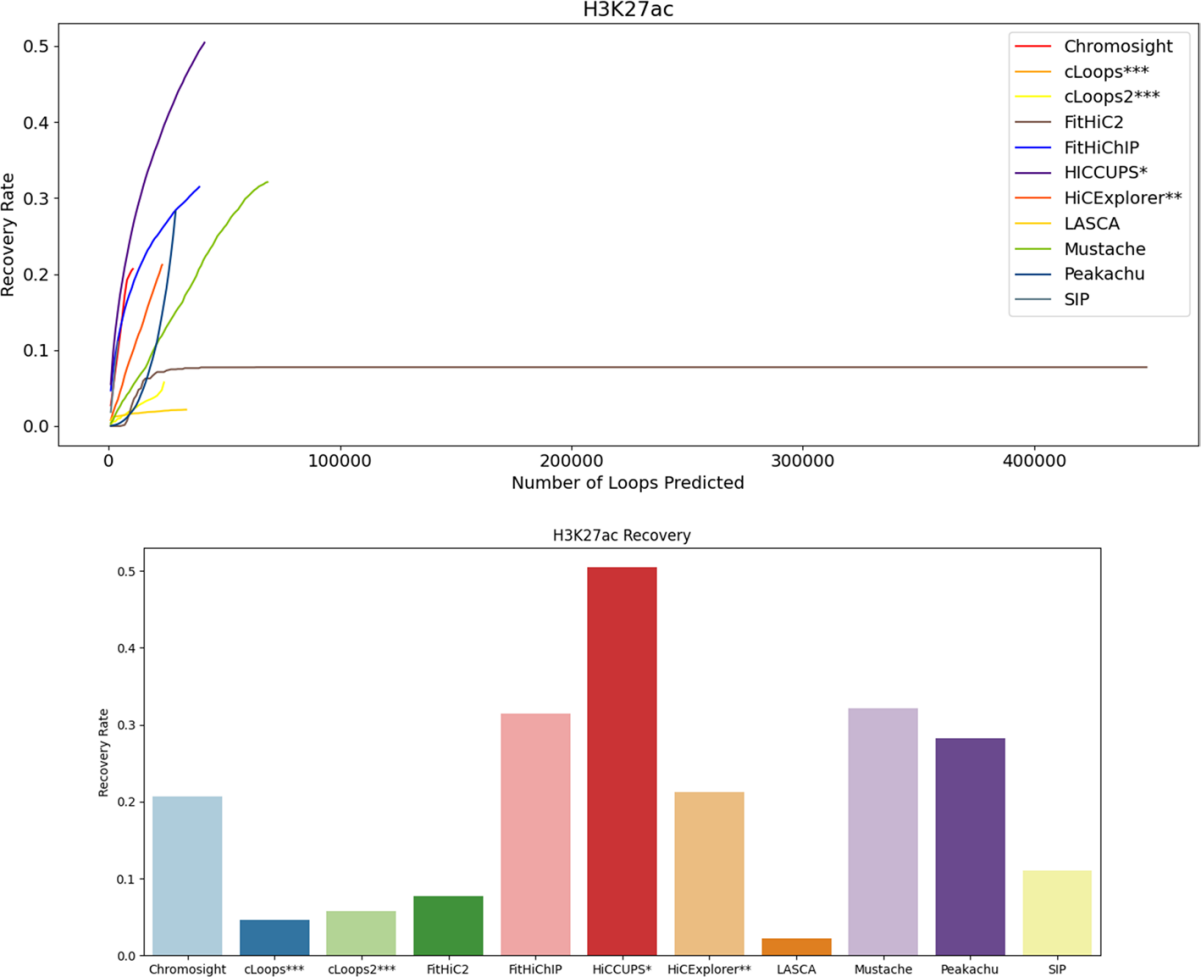
H3K27ac recovery recovery using primary GM12878 at 10 KB. HiCCUPS recovers most of the H3K27ac and LASCA recovers the least fraction of H3K27ac. HiCCUPS* and HiCExplorer** didn’t produce results at low resolutions and cLoops*** and cLoops2*** do not have a resolution parameter

**Fig. 7:**
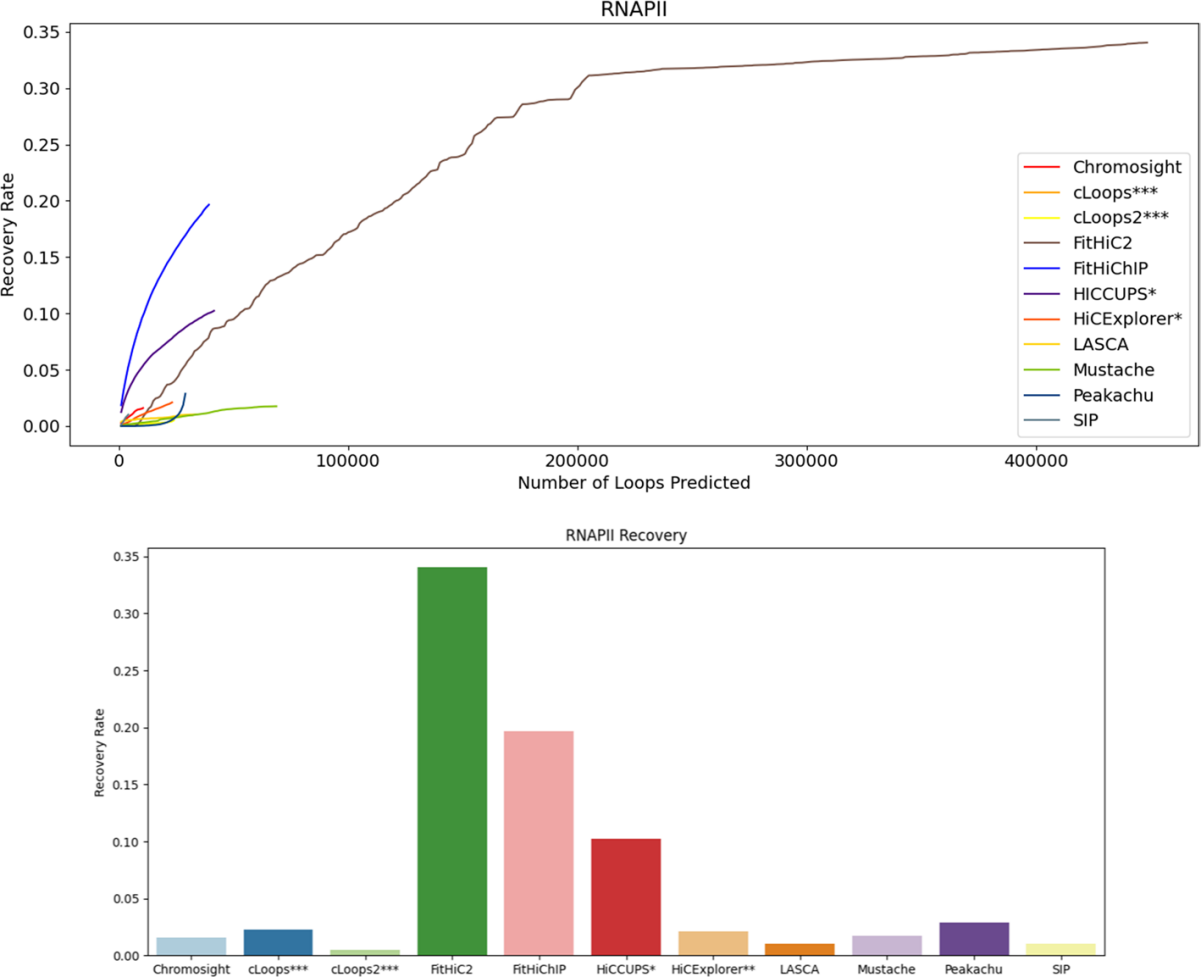
RNAPII recovery rate using primary GM12878 at 10 KB. FitHiC2 recovers most of the RNAPII and cLoops2 recovers the least fraction of RNAPII. HiCCUPS* and HiCExplorer** didn’t produce results at low resolutions and cLoops*** and cLoops2*** do not have a resolution parameter

To assess the robustness of each tool detecting relevant biological features, we calculated the recovery of specific biological features namely CTCF [40, 42], H3K27ac [25], and RNAPII [29] within the loops and scrutinized the results (Eq. 3). CCCTC-binding factor (CTCF) is a transcription factor that plays a crucial role in regulating the spatial organization of chromatin. It acts as an insulator protein, helping to define boundaries between different chromatin domains. Histone 3 Lysine 27 Acetylation (H3K27ac) are proteins around which DNA is wound to form nucleosomes and are often found near the promoters of actively transcribed genes. RNA Polymerase II (RNAPII) is an enzyme responsible for transcribing DNA into RNA during the process of transcription. The presence of RNAPII is a key indicator of active transcription. Each of these molecular components serves as distinctive markers or features for the nuanced analysis of chromatin loops. Evaluating the recovery of CTCF, H3K27ac, and RNAPII becomes paramount, signifying the proficiency of the analytical tools in precisely identifying or predicting these features within the intricate landscape of chromatin organization. We conducted comparisons in combination with CTCF, H3K27ac, and RNAPII using GM12878 primary full genome dataset at 5 KB, 10 KB, 100 KB and 250 KB (Figs. 5, 6, 7 and Additional file 1: Figs. S28, S29, S30, S31, S32, S33, S34, S35 and S36).

At 10 KB resolution data (Figs. 5, 6, 7), FitHiC2 exhibits an enhancement in RNAPII, which is the highest among other tools for primary datasets (Fig. 7). HiCCUPS recovers the highest CTCF and H3K27ac at 10 KB resolution (Figs. 5, 6). FitHiChIP, using GM12878 primary data, recovers more RNAPII at 10 KB resolution compared to HiCCUPS (Fig. 7). Mustache recovers a substantial amount across the three biological features at 5 KB resolution compared to 10 KB, 100 KB and 250 KB resolution (Additional file 1: Figs. S28, S29, S30, S31, S32, S33, S34, S35 and S36). In the case of KR normalized data, HiCCUPS and Peakachu recover almost the same number of loops, except for RNAPII at 10 KB resolution where they exhibit similar recovery rates (Additional file 1: Figs. S44, S45, S46 and S47). FitHiC2 recovers the majority of RNAPII at 10 KB resolution from normalized data, displaying the highest number of outlier points at 10 KB resolution (Additional file 1: Figs. S45, S46 and S47). Mustache recovers a noteworthy number of loops, positioning itself between HiCCUPS and Peakachu in most cases (Additional file 1: Figs. S39, S40, S41, S42, S43, S44, S45, S46, S47, S48, S49, S50 and S51). cLoops recovers the majority of CTCF at 5 KB, and HiCCUPS and Mustache recover most of the H3K27ac from replicate data (Additional file 1: Figs. S40, S42, S44, S46, S48 and S50). SIP, Chromosight, and HiCExplorer consistently exhibit competence in almost all cases. In summary, FitHiChIP, HiCCUPS and FitHiC2 demonstrate the highest recovery of CTCF, H3K27ac, and RNAPII biological markers or features in the loops identified.

Overall across the four resolutions considered for primary GM12878 genome, FitHiChIP, HiCCUPS, and FitHiC2 and FitHiChIP exhibits the highest recovery for CTCF (0.25), H3K27ac (0.45) and RNAPII (0.18) respectively, while LASCA shows the lowest amount of recovery rate in all three cases. Notably, Mustache, Peakachu, and Chromosight also demonstrate substantial recovery rate. These findings were further validated specifically for chromosome 1 and 6 at 5 KB and 10 KB resolution (Additional file 1: Figs. S28, S29, S30, S31, S32, S33, S34, S35, S36, S37, S38, S39, S40, S41, S42, S43, S44, S45, S46, S47, S48, S49, S50 and S51).

**Fig. 8:**
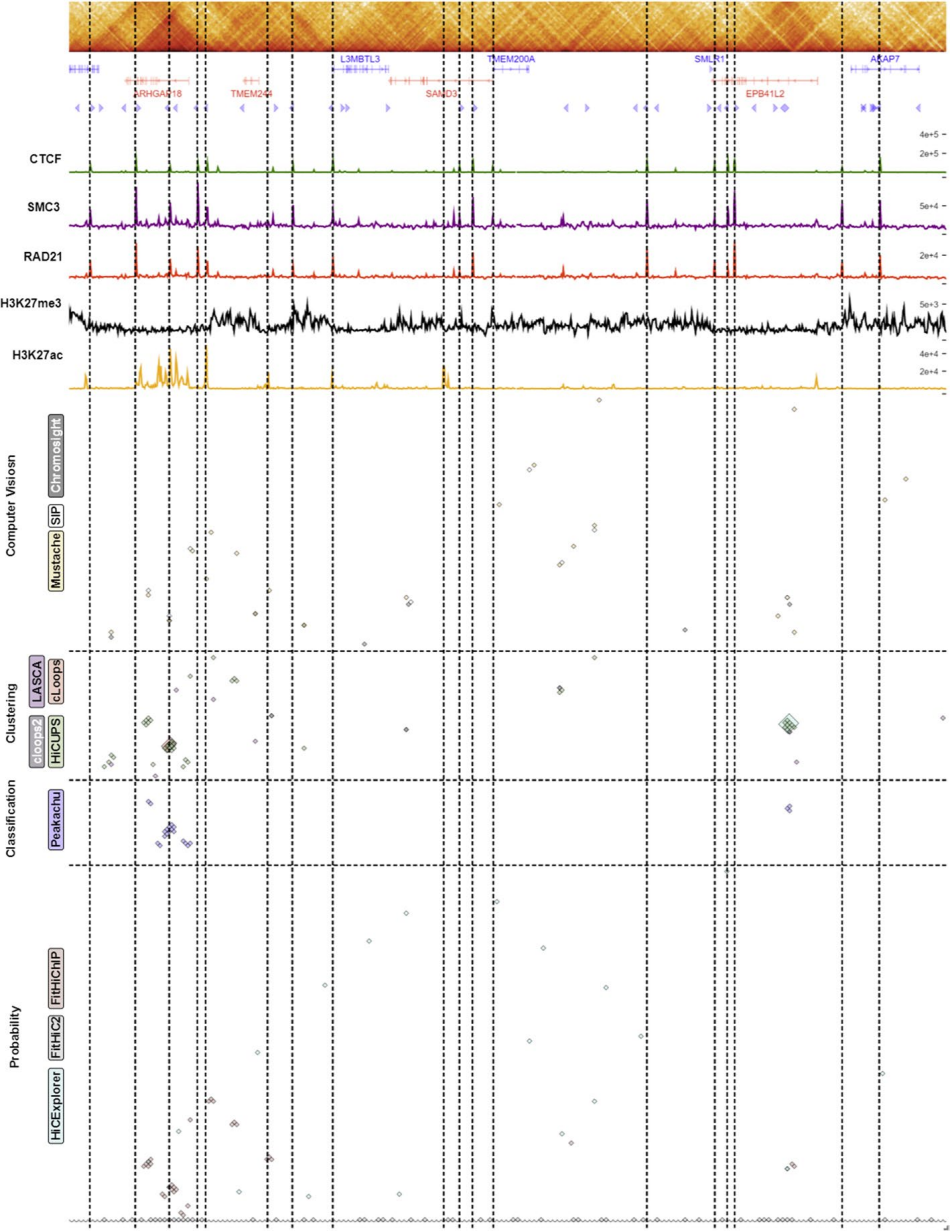
Enrichment signal representation in 129.7–131.6 M and 62.4–62.5 M for chromosome 6 (primary GM12878). This plot depictes gene annotation, CTCF motif orientation, and ChIP signals for CTCF, SMC3, RAD21, H3K27me3, and H3K27ac below the contact map (Plotted using HiGlass). Below this biological features enrichment data, we illustrated the loops identified by each of the algoritms across different categoiries and the vertical line marks the highest enrichment point for the signals identified

To visualize the biological significance within the loop area, we generated a ChIP-seq signal arrangement plot (Fig. 8) for each individual category, including the contact map, gene annotation, CTCF motif orientation, ChIP-seq signals of CTCF, SMC3, RAD21, H3K27me3, and H3K27ac. At the bottom, we included loops from different individual categories. Additionally, we incorporated separate categorical ChIP-seq signal plots for four tools in Additional file 1: Figs. S52, S53, S54 and S55. We selected a random region (129.7–131.6M and 62.4–62.5M) for all these plots and marked their biologically significant areas according to their loops. Figure 8 shows that SIP and Chromosight overlap in some areas, while Mustache and Chromosight exhibit a high signal of CTCF, SMC3, H3K27ac, H3K27me3, and RAD21 loops. HiCCUPS, cLoops, cLoops2, and LASCA overlap in some regions. HiCCUPS, LASCA, and cLoops show high signals for CTCF, SMC3, RAD21, H3K27ac, and H3K27me3 in some regions within this randomly selected region, and Peakachu demonstrates signal enrichment. FitHiC2, predicting a large number of contacts across the analysis, shows a high ChIP-seq signal around the selected region. HiCExplorer and FitHiChIP display ChIP-seq signals in some regions, and ChIP-seq signal enrichment from all these tools validates our recovery analysis with a visual representation.

#### Recovery efficiency analysis across loop callers

**Fig. 9:**
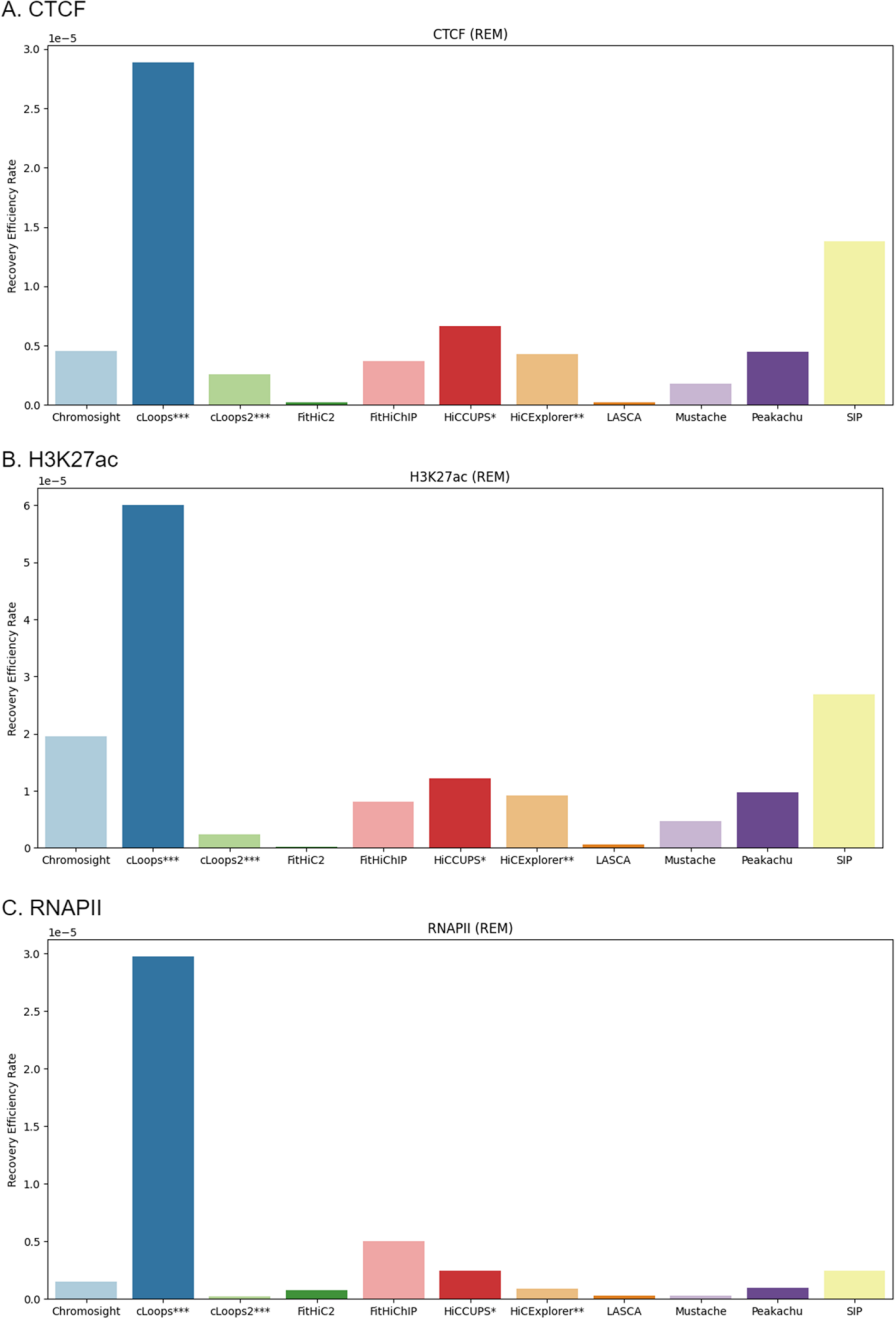
Recovery efficiency rate using primary GM12878 at 10  KB. cLoops has the highest recovery efficiency rate (REM) and LASCA has the least REM across CTCF, H3K27ac and RNAPII recovery. HiCCUPS* and HiCExplorer** didn’t produce results at low resolutions and cLoops*** and cLoops2*** do not have a resolution parameter

In assessing the efficacy of various loop detection methods, a notable observation arises when comparing their performance metrics. Some methods demonstrate a commendable ability to recover loops, achieving high rates of successful detection. However, a closer examination reveals that these methods often come hand-in-hand with a higher number of overall loop counts e.g. FitHiC2. On the other hand, there are alternative methods that exhibit comparable levels of recovery success while maintaining a notably lower count of detected loops e.g. HiCCUPS. This dichotomy in results prompts a crucial consideration in the evaluation process. Traditional metrics that solely emphasize recovery rates or loop counts may not encapsulate the nuanced performance of these methods adequately. Therefore, in assessing the biological correctness of the loops detected across the different tools, especially considering their varied success rates stemming from diverse counts of detected loops, it is essential to account for the potential impact of variations in the spread of loop detection counts. The inherent variability introduced by these diverse counts requires a careful consideration of the metrics used for assessment. To ensure a fair evaluation of correctness, we propose utilizing a recovery efficiency metric (*REM*) (“Recovery efficiency metric” section). This metric quantifies the recovery rate relative to the number of loops predicted. Consequently, regardless of the recovery rate for a biological feature, normalization is applied to prevent certain methods from disproportionately influencing the analysis by introducing excessive loops or mitigating the impact of approximations (Fig. 9 and Additional file 1: Figs. S37, S38 and S39). In our comprehensive investigation of the *REM* values for CTCF, H3K27ac, and RNAPII recovery across various tools, cLoops emerged as the leader with the highest *REM*, while LASCA displayed the least efficiency. A notable distinction lies in the absence of a resolution parameter in both cLoops and its updated version, cLoops2. This becomes particularly salient as our analysis was conducted at a fixed 10  KB resolution. This feature distinguishes cLoops2, as it directly analyzes paired-end tags (PETs) to identify candidate peaks and loops, estimating statistical significance with a permuted local background [25, 26]. Consequently, our analysis revealed a consistent loop count with cLoops, in contrast to other tools that rely on resolution-dependent contact matrices for loop detection. It is imperative to interpret the results in the Fig. 9 and Additional file 1: Figs. S37, S38 and S39 cautiously, considering this critical divergence in methodology, where cLoops operates independently of resolution-specific data for peak detection, while others do.

#### Evaluation of recovery performance across sequencing depth

To further validate the results of the tools and assess their performance under varying read coverages, specifically measuring sensitivity at high versus low depths of read coverage, we conducted a sequencing depth analysis using the recovery efficiency metric outcomes from the preceding section. Our analysis focused on GM12878 primary data, where we determined the recovery efficiency rate at high-sequencing depths (5 KB and 10 KB) in comparison to low-sequencing depths (100 KB and 250 KB).

**Fig. 10:**
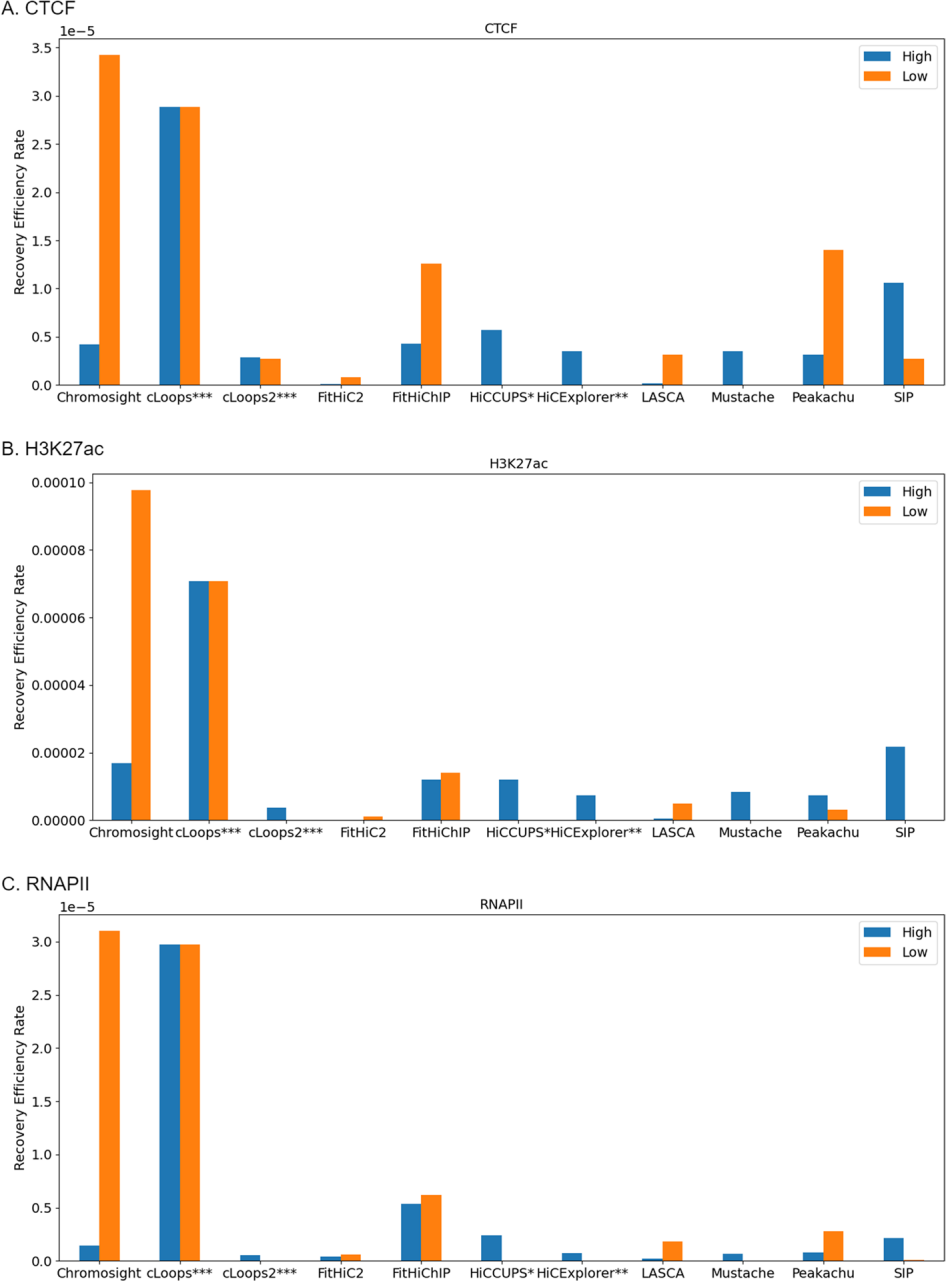
Recovery efficiency across sequencing depth (CTCF, H3K27ac, and RNAPII) using primary GM12878. cLoops showed the highest recovery consistency across high (5 KB, 10 KB) and low (100 KB, 250 KB) sequencing depths. HiCCUPS* and HiCExplorer** didn’t produce results at low resolutions and cLoops*** and cLoops2*** do not have a resolution parameter

We systematically calculated the *REM* value for key genomic features- CTCF, H3K27ac, and RNAPII at both high and low sequencing depths. This quantitative approach provides a comprehensive depiction of how consistently the tools make predictions across different depths. By examining the recovery efficiency of specific genomic elements, we gain insights into the tools’ reliability and accuracy across a spectrum of sequencing depths, contributing to a thorough understanding of their performance characteristics in diverse genomic scenarios (Fig. 10).

**Fig. 11:**
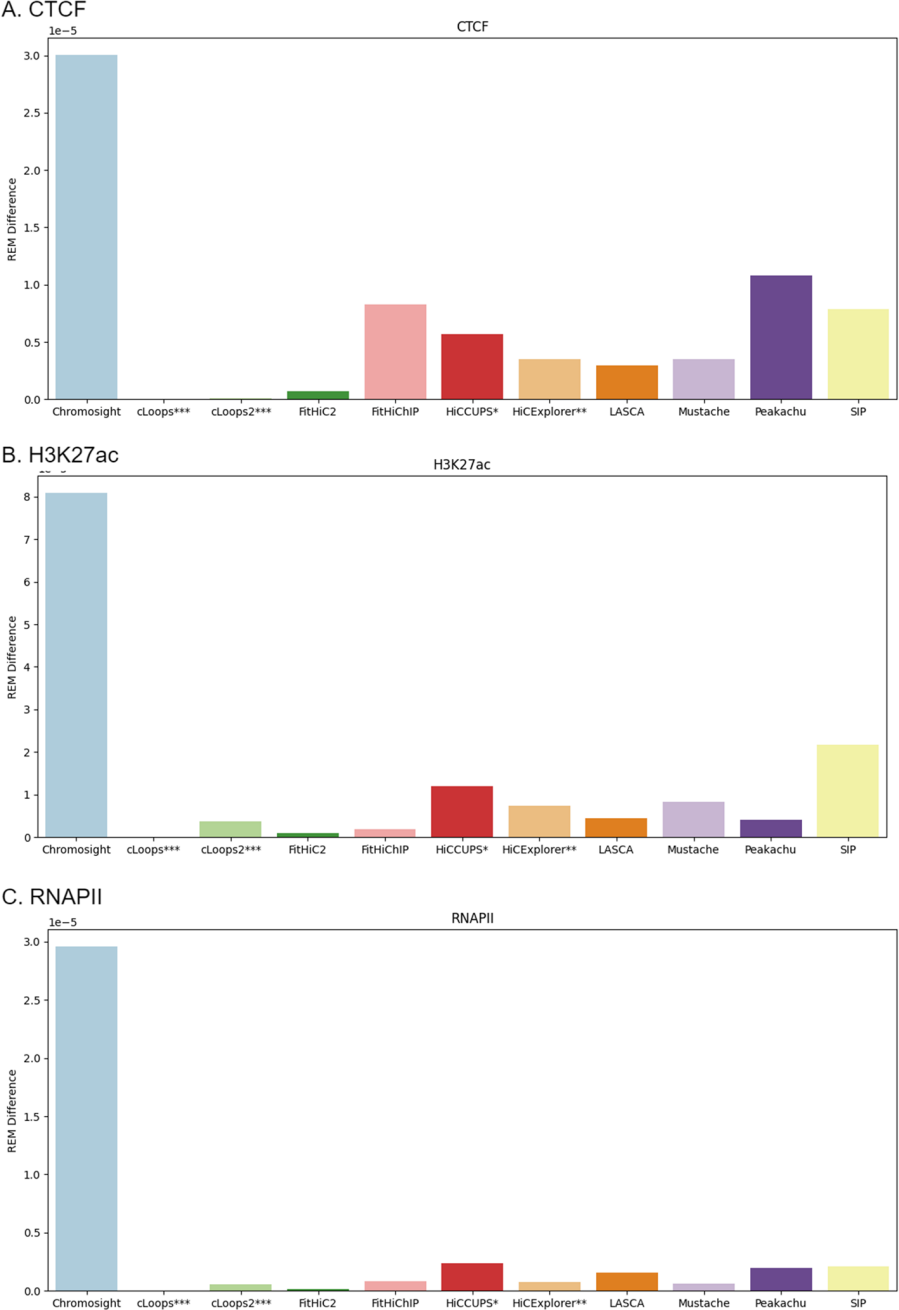
Recovery consistency across sequencing depth (5 KB, 10 KB, 100 KB, 250 KB) using primary GM12878. Here, higher REM difference value refers to lower consistency and vice versa. A lower score is considered better. HiCCUPS* and HiCExplorer** didn’t produce results at low resolutions and cLoops*** and cLoops2*** do not have a resolution parameter

cLoops consistently exhibited robust recovery performance across diverse sequencing depths, encompassing both high and low resolutions. As previously elucidated, the utilization of PETs by cLoops for loop detection underscores the stability of results observed across varying sequencing depths (Fig. 10). Across key genomic features, Chromosight shows the highest *REM* value at a low sequencing depth. Meanwhile, at high sequencing depths, SIP records a higher *REM* value compared to most methods for CTCF and H3K27ac. In the case of RNAPII, FitHiChIP records a higher *REM* compared to other tools at a high sequencing depth. It is worth noting that, HiCExplorer and HiCCUPS lack results at low sequencing depths, as they do not support this particular condition [27, 48]. This nuanced analysis provides valuable insights into the tools’ proficiency and limitations across diverse sequencing depths, offering a scientific understanding of their performance in recovering distinct genomic features. The difference between the *REM* values between the sequencing depth represents the consistency of each of the tools (“Consistency score” section) is provided in Fig. 11. Consequently, a lower value is indicative of superior consistency performance by the tools across various sequencing depths (Fig. 11, Tables 2).

**Table 2:** Consistency score using CTCF, H3K27ac and RNAPII recovery between high and low resolution

Tools	CTCF (%)	H3K27ac (%)	RNAPII (%)	Consistency score (%)
Chromosight	0.00	0.00	0.00	0.00
cLoops***	100.00	100.00	100.00	100.00
cLoops2***	99.70	95.35	98.20	97.75
FitHiC2	97.74	98.84	99.41	98.66
FitHiChIP	72.33	97.57	97.15	89.02
HiCCUPS*	81.07	85.19	91.95	86.07
HiCExplorer**	88.37	90.91	97.48	92.25
LASCA	90.06	94.55	94.63	93.08
Mustache	88.24	89.69	97.80	91.91
Peakachu	63.93	94.88	93.37	84.06
SIP	73.86	73.15	93.00	80.00

HiCCUPS* and HiCExplorer** didn’t produce results at low resolutions and cLoops*** and cLoops2*** do not have a resolution parameter

## Discussion

**Fig. 12:**
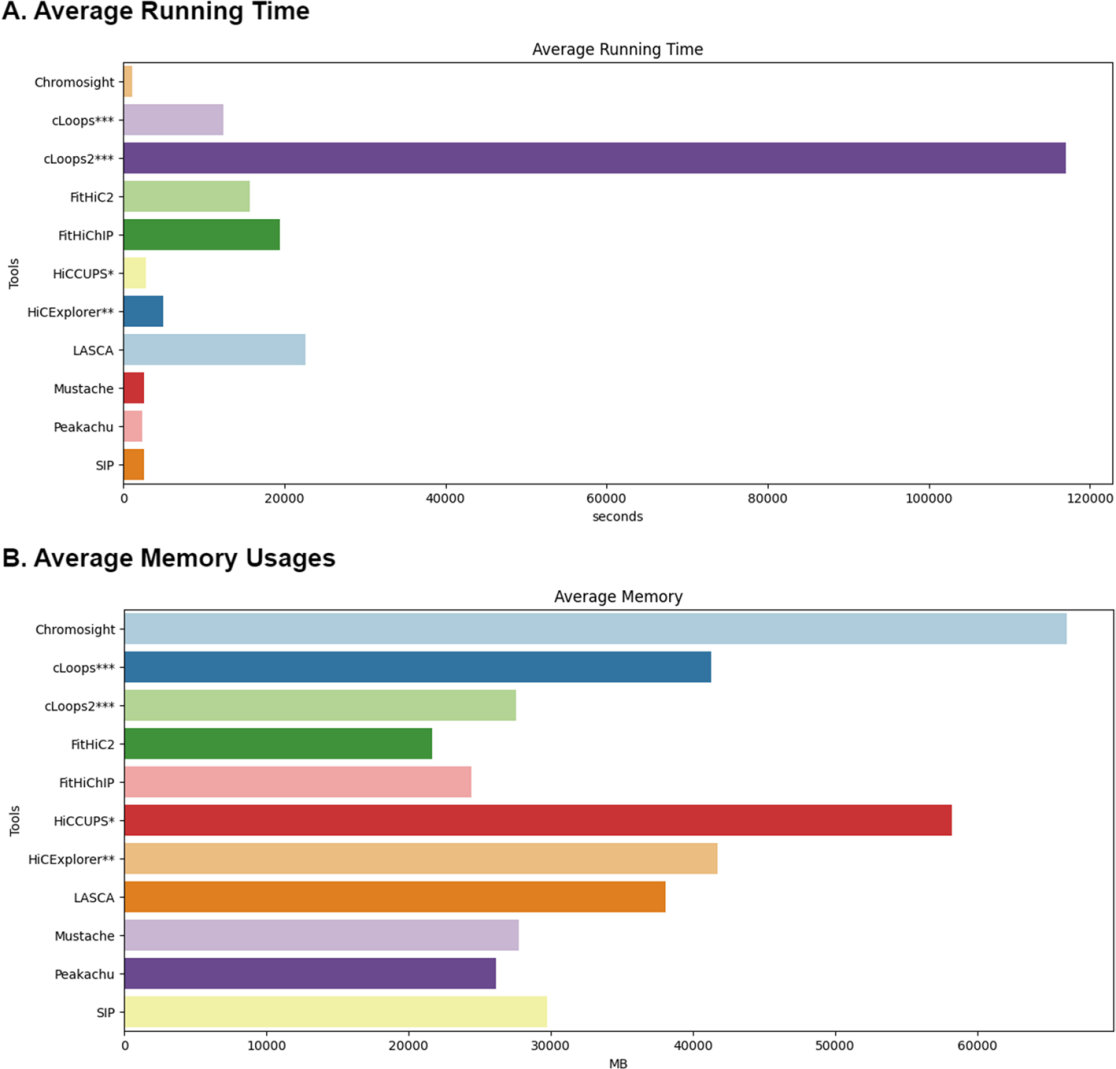
**A** Average running time and **B** Memory usages taken by all loop caller tools. Chromosight took the least amount of time and highest amount of memory. cLoops2 took the highest amount of time and FitHiC2 took the least amount of memory. HiCCUPS* and HiCExplorer** didn’t produce results at low resolutions and cLoops*** and cLoops2*** do not have a resolution parameter

**Fig. 13:**
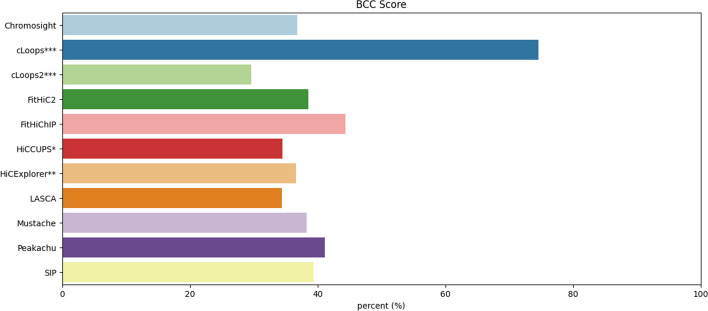
Performance of all the algorithms across the Biological, Computational and Consistency score metrics. The $BCC_{score}$ representation shows the weighted aggregated performance score for all the loop callers. Every score is calculated according to the description provided in the Methods section for $BCC_{score}$ calculation, and scaled to 100 for percentage representation, since the scores are the range 0–1. The scores represent the domain performance and $BCC_{score}$ represents the overall aggregated performance of every individual tool. HiCCUPS* and HiCExplorer** didn’t produce results at low resolutions and cLoops*** and cLoops2*** do not have a resolution parameter

Recent advancements in 3C-based sequence technology, as highlighted by Han et al. [17], have significantly expanded genome analysis capabilities. Loop prediction stands out as a pivotal aspect due to its relevance to various biological factors, including histone protein markers, intra and inter-chromosomal contacts, CTCF, and TAD regions. Over the past few years, a plethora of loop prediction tools has emerged, demonstrating proficiency across diverse biological aspects and datasets. In this study, we scrutinized 22 loop-calling tools, categorizing them into five distinct groups. Out of these, we successfully ran 11 tools using the same dataset and environment. Our benchmarking involved a comprehensive evaluation of biological features, encompassing the recovery results of CTCF, H3K27ac, and RNAPII, as well as considerations of running time, memory usages, computational robustness, and consistency. Each tool’s default parameters were considered within the same dataset to ensure a fair alignment. Every tool has its unique capabilities; hence, we assigned a percentage score of every tool according to their performance during our analysis (Fig. 13). The assessment covers three distinct categories:

- *Biological features* This includes the recovery of CTCF, H3K27ac, and RNAPII. The tools were evaluated based on how well they captured these biological features (Recovery Efficiency Metric). Combining these recoveries provide an overall assessment of the biological robustness of the tools. cLoops exhibited notable results in CTCF, H3K27ac and RNAPII recovery (Table 3). Chromosight and FitHiChIP also recorded significant results.
- *Consistency* This is evaluated using sequencing depth analysis. Tools are assessed for how consistently they perform across different sequencing depth datasets. We considered CTCF, H3K27ac and RNAPII recovery efficiency rate across high-sequencing depths (5 KB and 10 KB) in comparison to low-sequencing depths (100 KB and 250 KB) data. cLoops showed highest consistency, and cLoops2, FitHiC2, HiCExplorer, LASCA and Mustache demonstrated comparable consistency (Fig. 11 and Table 2).
- *Computational efficiency* This category involves two key metrics, the memory usages and running time. The running time analysis revealed that Chromosight, Mustache, Peakachu and SIP performed exceptionally well (Fig. 12A). The memory consumption analysis illustrated that FitHiC2, FitHiChIP and Peakachu performed exceptionally well (Fig. 12B). Combining the memory usages and running time, we introduced a computational robustness metric, where Peakachu demonstrated prominence. Except cLoops, cLoops2, HiCCUPS and Chromosight (Table 4), all other tools yielded commendable results in the computational category.

We used the $BCC_{score}$ to measure the overall performance of the tools. The $BCC_{score}$ calculates the weighted average among the categories and provides an overall performance assessment covering biological, computational, and consistency metrics. Based on our analysis, cLoops, FitHiChIP and Peakachu stood out as the most significant tools (Fig. 13). Table 5 provides a summary of the top-performing tools across various categories. In our analytical framework, we employ a three-tiered scoring system to categorize tools based on their performance, with three stars denoting excellence, two stars for good performance, and one star for fair performance. To arrive at this assignments in the context of this study, we meticulously organized the eleven methods from the highest performing to the least, subsequently assigning three stars to methods occupying positions 1–3, two stars to those in positions 4–7, and one star to methods in positions 8–11. The table includes running time as a separate metric to highlight the most efficient tools. Additionally, we benchmarked the tools parameters based on their simplicity and flexibility, noting variations in tool requirements. Some tools supports muilti-threads, normalization, multi-resolution and individual chromosome analysis. Tools that demonstrated flexibility with a variety of parameters received higher star ratings. Memory usage was not recorded due to varying tool configurations.

**Table 3:** Biological score averaging CTCF, H3K27ac and RNAPII recovery efficiency rate

Tools	CTCF (%)	H3K27ac (%)	RNAPII (%)	Biological score (%)
Chromosight	66.0440723	80.81	54.12	66.99
cLoops***	100	100.00	100.00	100.00
cLoops2***	8.311488926	1.88	0.00	3.40
FitHiC2	0	0.00	0.70	0.23
FitHiChIP	28.26098576	17.81	19.30	21.79
HiCCUPS*	18.46754736	16.26	7.33	14.02
HiCExplorer**	10.76000424	9.68	1.62	7.35
LASCA	4.244814356	3.00	2.48	3.24
Mustache	4.687381023	5.15	0.15	3.33
Peakachu	28.60921051	6.65	5.26	13.51
SIP	21.96560705	14.75	2.88	13.20

HiCCUPS* and HiCExplorer** didn’t produce results at low resolutions and cLoops*** and cLoops2*** do not have a resolution parameter

**Table 4:** Computational score averaging running time and memory usages

Tools	Running time (%)	Memory (%)	Computational score (%)
Chromosight	100.00	0.00	50.00
cLoops***	90.20	56.02	73.11
cLoops2***	0.00	86.77	43.39
FitHiC2	87.43	100.00	93.72
FitHiChIP	84.23	93.93	89.08
HiCCUPS*	98.57	18.01	58.29
HiCExplorer**	96.72	55.06	75.89
LASCA	81.45	63.26	72.36
Mustache	98.78	86.35	92.57
Peakachu	98.95	89.95	94.45
SIP	98.74	81.97	90.36

HiCCUPS* and HiCExplorer** didn’t produce results at low resolutions and cLoops*** and cLoops2*** do not have a resolution parameter

**Table 5:** Representation of every tool performance in different categories

Tools	Biological feature (CTCF, H3K27ac, RNAPII)	Consistency	Robustness in parameters	Computational efficiency: running time	Computational efficiency: memory
LASCA	*	**	*	*	**
cLoops***	***	***	**	**	*
cLoops2***	*	***	*	*	**
HiCExplorer**	**	**	***	**	*
FitHiC2	*	***	*	*	***
FitHiChIP	***	**	*	*	***
Peakachu	**	*	**	***	***
Mustache	*	**	***	***	**
Chromosight	***	*	*	***	*
SIP	**	*	**	**	**
HiCCUPS*	**	**	***	**	*

Star (*) symbol tells the performance category of every individual tools. Tools are categorized in terms of performance in three categories: Excellent (***), Good (**), and Fair (*). HiCCUPS* and HiCExplorer** didn’t produce results at low resolutions and cLoops*** and cLoops2*** do not have a resolution parameter

## Conclusion

Many algorithms for loop and peak analysis have been developed and proposed in recent decades; and each with its unique strength. These tools have been applied to various datasets, including ChIA-PET, Hi-TrAC, and Hi-C. Throughout our analysis, we successfully executed 11 out of the 22 methods that we examined. We found that the number of loop counts differs between tools, even when the data and resolution are the same. They also differ in same chromosome at different resolution. Also, the biological feature extraction performance evaluation showed that different tools hold distinct biological features that are not symmetric (Figs. 5, 6, 7, 9). Hence, there are variability in the types of biological features emphasized or recognized by different tools. Furthermore, certain tools computationally exhibited a greater degree of loop detection overlap across primary, replicate, and normalized dataset for a specific chromosome at a particular resolution compared to others (Fig. 4). While we have emphasized in this study that more loops do not necessarily indicate correctness, we proceeded with a biological evaluation to assess the consistency across sequencing depths (Figs. 10, 11). Our evaluation revealed notable consistency for certain tools, underscoring their reliability in capturing structural features within genomic data. Tools such as cLoops, and FitHiC2 exhibited higher percentages in the consistency evaluation, suggesting stronger reproducibility in their predictions. This observation reinforces the notion that these tools possess a higher degree of reliability in consistently capturing genomic structural features, as evidenced by our biological assessment. To provide an overall understanding of the performance of the different algorithms, we introduced the $BCC_{score}$ to serve as a quantitative measure covering the biological, computational, and consistency metrics. Using this metric, (a) we identified the top three algorithms, which exhibited a significant enrichment of biological features such as CTCF, H3K27ac, and RNAPII, showcasing their biological effectiveness. (b) Additionally, we determined the most consistent tool across sequencing depths, highlighting its reproducible performance in capturing biologically relevant information. (c) Furthermore, we pinpointed the most computationally effective method, considering factors like memory usage and running times.

Overall, this study stands as a novel comprehensive examination of Hi-C loop calling algorithms, offering a meticulous benchmarking assessment across various metrics. This analysis not only contributes valuable insights for the refinement of existing algorithms but also serves as a practical guide for future algorithm development and a useful resource for prospective users. In the ongoing development of new Hi-C loop calling algorithms, it is imperative to prioritize addressing issues related to data format, reproducibility, biological features, and bias. While acknowledging that performance is contingent on implementation, specific goals, and various factors, placing emphasis on these critical aspects can significantly enhance the robustness and reliability of outcomes. The diverse set of metrics employed in our benchmarking process ensures a nuanced understanding of algorithmic performance under various conditions. Hence, providing a groundwork and valuable resource for future development in genomic research.

## Methods

Many tools and techniques have been developed for loop and peak detection. These algorithms have used different methods and approaches in their implementations based on the underlying objectives and hypotheses. Here, we categorized the tools into five distinct categories (Table 6) according to their base algorithm and we briefly describe them. All the tools are described briefly following their category in the Additional file 1: Document.

**Table 6:** Tools categories by methodology

#	Category	Tool
A	Clustering based	i. LOOPbit [23]
		ii. LASCA [24]
		iii. cLoops [25]
		iv. cLoops2 [26]
		v. HiCCUPS [48]
B	Probability-based	i. HiCExplorer [27]
		ii. HiC-ACT [28]
		iii. FitHiC [29]
		iv. FitHiC2 [30]
		v. FitHiChIP [31]
		vi. GOTHiC [32]
		vii. HiC-DC [33]
		viii. ZipHiC [34]
		ix. NeoLoopFinder [35]
		x. HMRF Bayesian caller [36]
C	Classification based	i. FIREcaller [37]
		ii. Peakachu [38]
D	Computer vision based	i. Mustache [40]
		ii. Chromosight [42]
		iii. SIP & SIPMeta [41]
		iv. DeepLoop [49]
E	Pile-up procedure based	i. Coolpup.py [39]

All the tools are divided into five distinct categories according to their implementation method

### Clustering based

Clustering algorithms such as DBSCAN [50, 51], derived cDBSCAN and HDBSCAN [52] have been used as the central algorithm in the development of some loop and peak detection algorithms such as cLoops [25]. DBSCAN algorithm does not consider the spatial organization of input data nor biased with noise data. DBSCAN performs clustering using $\alpha$, a radius from where it will decide its core and border points, and $\Delta$, a threshold value representing a minimum point in a cluster; otherwise, they would be considered noise. It starts scanning considering a point and expands the area considering a radius, $\alpha$, and with this radius, all points are core points and considered to be in the same neighborhood, and if any points are not within this area, those are considered noise. This algorithm has a running time complexity of $O(n\log (n))$ [50] that depends on the distance calculation algorithm and could go up to $O(n^3)$ [51]. We described all the clustering-based loop prediction tools in the Additional file 1: Document (Sect. 1.1).

### Probability based

Another category that we have identified to which most of the loop and peak detection tools belong is the Probability-based category. Specifically, tools in this category apply the binomial distribution, Hidden Markov model (HMM), Cauchy distribution, and others to aid the loop and peak detection. HiCExplorer has many features with loop prediction and it uses binomial distribution, FitHiC uses statistical confidence estimation to calculate midrange intra-chromosomal contacts whereas FitHiC2 is the updated version of FitHiC. We briefly described all the tools in the Additional file 1: Document (Sect. 1.2) and in the following, we described different types of distribution algorithms.

Binomial distribution is a success or failure outcome function where the experiments iterate multiple times, and this is similar to the Bernoulli distribution. There are three preconditions for applying binomial distribution: i. observation or trials number is fixed, ii. observation or trials are independent and iii. success probability is the same for all the trials. Formally, we can state the binomial distribution as a function with a coefficient value and parameters, $t =$ total number of independent trials, $r =$ probability of success, $m(1-r) =$ probability of failure, and $$\left( {\begin{array}{c}t\\ z\end{array}}\right) =$$ binomial coefficient

1 $$\begin{aligned} bdist(z,t,r) = \left( {\begin{array}{c}t\\ z\end{array}}\right) \times r^z \times (1-r)^z \end{aligned}$$

The HMM is a generalized statistical modeling formula for linear problems such as sequence, time series, and computational biology [53]. Mathematically, we can apply the HMM as there is a hidden process $H_{n/t}$, and emission probability $P(S_{n/t} \in B | H_n = h_n or H_t \in fB$ where $H_n$ is a Markov process, *B* is each Borel set, and *fB* is each family of Borel set. For discrete time stochastic processes, $n\ge 1$, and continuous-time stochastic processes, $t\le t_0$. It starts from an initial state and continues until the end state generating a sequence of states based on state probabilities. This state sequence is a Markov chain where every next state depends on the current state, observing the symbol sequence hiding the state sequence.

Cauchy distribution is a continuous probability distribution closely related to the Poisson kernel. Cauchy distribution is useful in many domains such as mechanical, electronic fields, and financial analysis [54]. We can describe Cauchy distribution as

2 $$\begin{aligned} f(\chi ) = \frac{1}{\pi \alpha (1+(\frac{\chi -\chi _0}{\alpha })^2)} \end{aligned}$$

where $\chi _0 =$ location parameter and $\alpha =$ scale parameter [54]. If $\chi _0 = 0$ and $\alpha = 1$, it is called standard Cauchy distribution.

### Classification based

The third group of loop and peak detection algorithms that we have identified is the classification-based tools. Classification is a supervised machine learning approach that is based on training a classifier or model on labeled examples. This accurately labels unlabeled and unknown datasets introduced to this classifier. Several classification algorithms have been introduced over the years such as Decision Tree, Naive Bayes, and K-Nearest Neighbor, and are used in various domains such as fraud detection and medical diagnostics [55, 56]. In bioinformatics, scientists are using classifiers to solve their problems such as cancer cells, and loop and peak detection [57, 58]. Peakchu is a random forest classification-based tool to predict loops which are described briefly along with other tools in Additional file 1: Document (Sect. 1.3).

### Computer vision based

Computer vision (CV) offers access to information such as labels, object structure, shape, and much more meaningful information by analyzing images, video frames, and signals. Over the decades, computer vision algorithms have been used to make notable impacts in image classification, object detection, and recognition in robotics and autonomous vehicles. With the advent of high-resolution microscopes, we have access to biological images that can fit into computer vision algorithms for output. To support this, many CV algorithms have been proposed [40–42] and there are certain tools for loop calling from Hi-C datasets using CV techniques such as Mustache [40], DeepLoop [49]. We describe Mustache, SIP and SIPMeta, Chromosight, and DeepLoop in the Additional file 1: Document (Sect. 1.4).

### Pile-up procedure based

Pile-up is a generalized procedure that averages a certain number of data from a given dataset such as averaging 3D points in a specific region from a 3D matrix. It describes the tendency of relation within multiple points/regions. It can be considered to be similar to the normalization technique and quantifies the averaged value with the expected one. We briefly stated Coolpup.py, a pile-up procedure-based loop detection tool in the Additional file 1: Document (Sect. 1.5).

### Data formats

Hi-C is a 3C-based sequence technique that facilitates high-resolution conformation capture for chromosome analysis [12, 59]. This data can be used to represent and understand genome-wide features in 3D space (e.g. chromatin interaction, genomic structure, TAD, chromatin loops). To efficiently represent the Hi-C data, researchers developed *.hic* [12], *.cool* [60], *.mcool* [60], and other representational formats. Hi-TrAC is another technique for genome-wide interaction profiling at a high resolution [9]. We represented all the input and output formats used in loop and peak calling tools in Table 7. The *.cool* format represents Hi-C data in three columns (bin, chromosome, and pixel) and index [60]. The *.mcool* format is a different representation on the *.cool* format having multiple-resolution data. The .hic is a highly compressed binary file for fast random access containing multiple resolution contact matrix [12]. The *.bed* and *.bedpe* are developed to represent genomic data. The *.bed* (Browser Extensible Data) format contains a maximum of 12 columns (chrom, chromStart, chromEnd, name, score, strand, thickStart, thickEnd, itemRgb, blockCount, blockSizes, and blockStarts) where the first three are required [61]. Another format is *.bedpe* (containing chrom1, start1, end1, chrom2, start2, end2, name, score, strand1, strand2, and user-defined fields) was introduced to represent interchromosomal features for variation analysis of the chromosome structure [62]. The *.sam* is a sequence alignment or a map format developed by Li et al. [63]. It is a tab-separated text format having an optional header section and alignment section. The alignment section has 11 fields (QNAME, FLAG, RNAME, POS, MAPQ, CIGAR, RNEXT, PNEXT, TLEN, SEQ, QUAL) and the @ symbol separates the header section from the alignment section. The *.bam* (binary alignment map) is the binary representation of the *.sam* format [63]. The *.hdf5* (hierarchical data format version 5) is an open-source data format that supports large, complex, and heterogeneous data in a single file and acts like a file system [64]. The *.h5* is developed based on the *.hdf5* container. It has a specific structure describing intervals, matrix, distance count, nan_bins, and correction_factors. The *.rds* (Ray Dream Studio) is a 3D object file extension that is serializable and compressible into a smaller size. The *.bedGraph* is a track format that can hold continuous-valued data such as chromosome name, start, end, and data value [61]. It is similar to the wiggle format and suitable for transnational and probability score data. The *.clpy* is the Coolpup.py defined custom data format for storing pileup results from the method pipeline [39].

**Table 7:** Loop caller algorithms overview (input, output, resolution)

#	Tool	Year	Sequence data	Input format	Input resolution	Output format
1	FitHiC [29]	2014	Hi-C, ChIA-PET	.txt		.txt, .gz
2	HMRF Bayesian caller [36]	2015	Hi-C	.txt	$\le 10$ KB	.hdf5, .txt
3	HiC-DC [33]	2017	Hi-C	.bam	$\le 5$ KB	.rds
4	GOTHiC [32]	2017	Hi-C	.bam, .sam	$\le 1Mb$	.gz
5	cLoops [25]	2019	ChIA-PET, Hi-C, HiChIP, TrAC-looping	.bedpe	$\le 5$ KB	.txt, .pdf
6	FitHiChIP [31]	2019	HiChIP, Hi-C	.txt, .gz, .sizes	$\le 5$ KB	.bed
7	FitHiC2 [30]	2020	Hi-C	.txt	$\le 40$ KB	.txt
8	FIREcaller [37]	2020	Hi-C	.gz, .hic, .cool	40 KB	.txt
9	Peakachu [38]	2020	Hi-C, Micro-C, ChIA-PET, HiChIP, PLAC-Seq, Capture Hi-C	.cool, .hic, .bedpe	10 KB	.bedpe
10	Coolpup.py [39]	2020	Hi-C	.bed, .cool, .mcool, .hic, .tsv	$\le 10$ KB	.clpy
11	Mustache [40]	2020	Hi-C, Micro-C	.txt, .hic, .cool, .mcool	$\le 10$ KB	.tsv
12	SIP & SIPMeta [41]	2020	Hi-C	.hic, .mcool, file	$\le 5$ KB	file
13	Chromosight [42]	2020	Hi-C, ChIA-PET, DNA SPRITE, HiChIP, Micro-C	.cool	$\le 10$ KB	.tsv
14	LASCA [24]	2021	Hi-C, ChIP-seq	.cool	$\le 10$ KB	.bedpe
15	cLoops2 [26]	2021	ChIC-seq, Hi-TrAC/TrAC-looping	.bedpe	$\le 1$ KB	.txt
16	HiC-ACT [28]	2021	Hi-C	.txt	$\le 10$ KB	file
17	NeoLoopFinder [35]	2021	Hi-C	.mcool	10 KB	.bedGraph
18	LOOPbit [23]	2022	Hi-C	.tsv	5 KB	.tsv
19	HiCExplorer [27]	2022	Hi-C	.bam, .sam	$\le 10$ KB	.h5
20	ZipHiC [34]	2022	Hi-C	.csv	$\le 10$ KB	data frame
21	HiCCUPS [48]	2014	Hi-C	.hic	$\le 5$ KB	text
22	DeepLoop [49]	2022	Hi-C	heatmap	$\le 5$ KB	heatmap

Each column denotes the information about the algorithm in order: the tool name, the year released, the 3C-based sequences data it accepts, the input data file format, the accepted input data resolution, and the output data file format. All the tools have different input and output formats, sequence data, and recommended input resolutions. It is worth noting that often many of the tools accept 3C-based data with resolutions lower than the ones stated in the table. The reported resolution for each tool is based on what was used by the authors in their manuscripts

### Analysis methods

#### Overlap

Overlap defines the common loops between different loop prediction tools’ results. Here, we used https://github.com/ay-lab/FitHiChIP/tree/master/UtilScript to draw the overlap between primary, replicate, and normalized data for a specific chromosome at a specific resolution. We used 50 window sizes to determine the overlaps. This produces results in two ways, (1) comparing with a reference loop file, and (2) producing a master interaction file from the provided files merging them all together. We used the master interaction file generated from our loop files. First, it generates master interaction files from the loop files storing all the loop information and then sorting them. It receives up to 5 interaction files to draw the diagram. Next, it finds the overlap indices between the merged file and the input files and determines the unique overlap indices from the overlap indices.

#### Recovery

We computed CTCF, H3K27ac, and RNAPII recovery using different loop prediction results. This recovery reports the biological consistency of a tool. The main procedure for recovery analysis is almost same as overlap analysis. Recovery analysis requires two input files i) a reference file to be matched, and ii) a loop file with q-value column. It sorts the input file with q-value and then finds the overlap indices between the loop file and the reference file. It first defines the overlap between files and then only keeps the unique overlap indices to get the overlap statistics. It uses a window size to calculate the overlap and we used 50 window size in our analysis. Then it calculates a recovery rate in every thousand count with the reference rows. We can write it as

3 $$\begin{aligned} f = \frac{l}{T_{ref}} \end{aligned}$$

where $f =$ recovery rate in every thousand, $T_{ref} =$ number of records in reference file, and $l =$ length of overlap. To compute this recovery, we used https://github.com/ay-lab/Utilities/tree/main/Recovery_Plot_FitHiChIP script in our manuscript.

#### Recovery efficiency metric

This metric computes the performance per input, specifically focusing on recovery rate, and subsequently normalizes it based on the number of loops. This approach enables the evaluation of each loop calling algorithm independently, ensuring a fair assessment that accounts for the varying number of detected loops. The normalization step ensures that the analysis remains unbiased, preventing tools from disproportionately influencing the results by introducing an excessive number of loops.

4 $$\begin{aligned} REM = \frac{f}{LC} \end{aligned}$$

$REM =$ Recovery Efficiency Metric, $f =$ recovery rate, and $LC =$ number of loops.

#### Peak

We used https://github.com/XiaoTaoWang/HiCPeaks to generate the peak plots. Here, we used 20 M to 25 M regions to observe the peaks from the loop file. First, it generates the heatmap using the contact matrix file at the given specific regions. After that, it parses the loop file to determine the positions of loops. It creates a loop table with chromosome numbers and, the start and end positions of loops. Using this table, mark the positions in the heatmap to indicate the loops.

#### APA

To determine the APA score we used https://github.com/XiaoTaoWang/HiCPeaks. First, it determines peak regions from the loop file. After that, with these positions, the interaction matrix file, and the provided window size, it generates an APA submatrix. It takes each value of a square region according to the window size centering the peak position and divides each value with the mean value of those regions generating a submatrix at the end. We write it

5 $$\begin{aligned} M_{APA} = \frac{V_{ij}}{V_{mean}} \end{aligned}$$

where $M_{APA} =$ APA submatrix, $V_{ij} =$ square region from a peak position according to the window size, *w* (we used window size, $w=5$), $V_{mean} =$ mean value of the square region $V_{ij}$, and $i = (i-w, i+w)$ and $j = (j-w, j+w)$. Then from the submatrix, it creates a mean value list for every row of the submatrix to remove the outliers and determine the percentils. Next, it determines the average value from the submatrix and calculates the lower positions matrix using the average values up to the limit of corner size (we used 3). Finally, it calculates the APA score by dividing the average value within the window by the lower position mean value. We write it

6 $$\begin{aligned} S = \frac{A_{avg}}{l_{mean}} \end{aligned}$$

where, $S =$ APA score, $A_{avg} =$ Average APA values, and $l_{mean} =$ lower position mean value.

#### Consistency score

To determine the consistency score, we used sequencing depth values of each tool. First, we calculated the average of CTCF, H3K27ac and RNAPII *REM* values at high resolution, $High_{avg}$, (5 KB and 10 KB) and at low resolution, $Low_{avg}$, (100 KB and 250 KB). Second, with this calculated $Low_{avg}$ and $High_{avg}$, we compute the Consistency score as follow:

7 $$\begin{aligned} Con_{score} = |Low_{avg} - High_{avg}| \end{aligned}$$

It is noteworthy that for methods lacking $Low_{avg}$ value, we directly utilized the singular $High_{avg}$ value. Such tools have been identified in our analysis.

#### BCC score

To determine the robustness of the tools, we categorize our analysis in three category (Biological, Consistency and Computational) and introduced $BCC_{score}$ to compute overall score. $BCC_{score}$ calculates the weighted average score among all the features where users can assign their weights according to their usecase to find the robustness. It is a flexible score function where user can include more categories according to their analysis. We stated $BCC_{score}$ as

8 $$\begin{aligned} BCC_{score} = \frac{Bio_s\times W_{bio} + Con_s\times W_{con} + Com_s\times W_{com}}{W_{bio} + W_{con} + W_{com}} \end{aligned}$$

where $Bio_s =$ biological feature score, $W_{bio} =$ weight for $Bio_s$, $Con_s =$ consistency score, $W_{con} =$ weight for $Con_s$, $Com_s =$ computational score, and $W_{com} =$ weight for $Com_s$. In our analysis, we used CTCF, H3K27ac and RNAPII as biological feature score (Eq. 3) and assigned $W_{bio} = 2$ because the biological correctness of a predicted loop is more valuable, at least twice more valuable, and relevant for downstream analysis. We anticipate that the users can modify this weight, as needed, in future analysis to signify how important they rate biological correctness among several other features they include or incorporate into the $BCC_{score}$. We computed the consistency score using (Eq. 7); and computed the computational score using the average of the normalized running time and memory consumption scores with $W_{con} = W_{com} = 1$. The $BCC_{score}$ is computed by normalizing all category scores through Min-Max normalization. This transformation ensures that the minimum value becomes 0, the maximum becomes 1, and all other values are expressed as decimals between 0 and 1. Consequently, the $BCC_{score}$ yields a value between 0 and 1, where higher values indicate better performance.

### Supplementary information

**Additional file 1.** Supplemental Document provides brief methodology of each tools, Supplemental Tables provide the raw results encountered during our analysis and Supplemental Figures provide additional plots of our analysis.

## Data Availability

The Hi-C contact maps of GSE63525 GM12878 were downloaded from NCBI GEO. We used GSM1872886 as CTCF reference, GSE101498 for H3K27ac reference, and GSM1872887 as RNAPII reference file for biological feature analysis. These files are available in NCBI GEO. We used ChIP-seq signal data from the USCS Genome Browser which is available on HiGlass server. We used HiGlass server’s preloaded CTCF motif orientation file and gene annotation file during our analysis. All scripts and programs used to benchmark these loop calling tools are available at https://github.com/OluwadareLab/Comprehensive_Loop-Caller_Benchmark.
