# Supplementary material for: Comparative study on chromatin loop callers using Hi-C data reveals their effectiveness
Source: BMC Bioinformatics. 2024 Mar 21;25:123. doi: 10.1186/s12859-024-05713-w (PMC10958853; doi:10.1186/s12859-024-05713-w)
Supplement: Supplementary file 1 — Additional file 1. Supplemental Document provides brief methodology of each tools, Supplemental Tables provide the raw results encountered during our analysis and Supplemental Figures provide additional plots of our analysis. [file 12859_2024_5713_MOESM1_ESM.pdf]

Comparative study on chromatin loop callers using Hi-C data reveals their effectiveness

H. M. A. Mohit Chowdhury[1], Terrance Boult[1], Oluwatosin Oluwadare[1,2*]

[1]Department of Computer Science, University of Colorado at Colorado Springs, 1420 Austin Bluffs Pkwy, Colorado Springs, 80918, CO, USA.
[2]Department of Biomedical Informatics, University of Colorado Anschutz Medical Campus, Aurora, CO, USA.

*Corresponding author(s). E-mail(s): ooluwada@uccs.edu;
Contributing authors: hchowdhu@uccs.edu; tboult@uccs.edu;

1 Supplemental Document

1.1 Clustering-Based Tools

1.1.1 LOOPbit

Galan et al.[1] developed LOOPbit for classifying CTCF-CTCF pairs. LOOPbit uses HDBSCAN and CNN algorithms in its pipeline and CNN helps to predict loop localization in the Hi-C interaction matrix. They used ChIP-seq and Hi-C data for their experiment and tested the reproducibility with Jaccard Index and calculated biologically relevant benchmarks. LOOPbit prepares its data using MEME, FIMO, and TADbit tools[2–4]. It performs compartmentalization with TADbit tools and uses APA[5] steps for generating the sub-matrices. It defines non-overlapping peaks and performs CTCF peak aggregation with *Meta-Waffle*. After this step, LOOPbit preforms analysis, deconvolution and classifies submatrices into micro-clusters or neurons with a Self-Organizing Feature Map (SOFM)[6] process. It decides the optimal parameters for SOFM considering three evaluation matrices: i. classification submatrices percentage, ii. neuron variability, and iii. compartment segregation score. LOOPbit finds the CTCF-CTCF interaction patterns with the SOFM neuron coordinates, performs clustering with Uniform Manifold Approximation and Projection(UMAP), and then applies the HDBSCAN algorithm for further clustering. It merges all the classes

into the four classes of promoter, enhancer, repressed polycomb, and heterochromatin. Next, LOOPbit sections off the genome into $5KB$ bins and accordingly classifies them based on overlap $> 50bp$. One or more categories could have one bin. Finally, LOOPbit classifies these interaction bins as not expected, promoter-enhancer loop, and heterochromatin-heterochromatin.

1.1.2 LASCA

Luzhin et al.[7] introduced the loop and significant contact annotation pipeline (LASCA) for identifying the loop and enhancer-promoter interactions. LASCA is a Weibull distribution-based modeling. It provides users the ability to tune the parameters and steps according to their choice. LASCA follows three basic processes in its entire life cycle. First, each diagonal corrected Hi-C matrix goes through Weibull distribution-based statistical background model. After that, LASCA calculates P-values of each pixel representing probability, and that leads to tracing model pixels in the same or higher intensity and calculating Q-values performing FDR correction in P-values. This Q-value may be corrected again, and user can set the threshold value for the Q-value with a default at 0.1. Next, LASCA clusters all the significant groups having a minimum of three pixels in each cluster by DBSCAN. It assigns pixels in neighbors by calculating the maximum Euclidean distance to be 1 between two pixels related to a particular cluster. The user specifies the cluster size, and it defines each cluster center; these center coordinates are loop coordinates. Finally, the identified loops may go through various filters such as signal over background enrichment, signal intensity, signal decay, and signal enrichment over random signals.

1.1.3 cLoops

Most of the loop calling tools are confined to single data types, depend on contact matrix resolution, or require expensive hardware. Cao et al.[8] proposed cLoops to overcome these issues. They used the cDBSCAN algorithm for loop calling in 2D space and achieved $O(n)$ running time in the most ideal situation. They enhanced the DBSCAN algorithm for loop calling by adding an indexing technique to lessen noise and improve neighbor search. Like the original DBSCAN algorithm, the cDBSCAN algorithm uses two inputs (radius and threshold) with the same meaning. In addition, it marks minimum 2D genomic coordinates, indexes each coordinate, and assigns each point to a square. Then, cDBSCAN determines the noise squares within two steps: i. finding the potential noise indexes, and ii. finding the already detected noise indexes for cross-checking. For noise identification, they used the K-Nearest Neighbor idea and introduced an indexing technique that reduces the search space. The cDBSCAN performs clustering the same as the original DBSCAN algorithm with $3l \times 3l$ square for neighbor search where $l =$ square side length. The main cLoops is a two-step loop calling algorithm and takes mapped PET data formatted in *.bedpe*. It uses the cDBSCAN for loop and peak calling, and the result could be used to find significant combinations and loop boundaries. The cLoops compares the candidate's loops' PETs number with

the permuted local backgrounds (PLBs) for determining their significance.

$$Hypergeometric\ Test = 1 - \sum_{n=0}^{P_{i,j}-1} \frac{\binom{Tr_j}{n}\binom{Tp-Tr_j}{Tr_i-n}}{\binom{Tp}{Tr_i}} \tag{1}$$

where P = linking anchor PETs, Tr = specific region total PETs and Tp = total PETs

$$Poisson\ Test = 1 - \sum_{n=0}^{P_{i,j}-1} \frac{\eta^n \epsilon^{-\eta}}{n!},\ \eta = \sum_{i,j} \frac{P_{i,j}}{\rho_{i,j}} \tag{2}$$

where ρ = permutation region number

$$Binomial\ Test = 1 - \sum_{n=0}^{P_{i,j}-1} \binom{Tp}{n} \psi_{i,j}^n (1 - \psi_{i,j})^{Tp-n} \tag{3}$$

where ψ = single PETs link observation possibility. cLoops uses a hypergeometric test, Poisson test, and binomial test for determining statistical significance of candidate loops to increase the precision.

1.1.4 cLoops2

cLoops2[9] is an updated version of cLoops[8]. It has extra functionalities that can be run separately such as outputting uniquely high-quality mapped PETs from the FASTQ file. Cao et al.[9] improved the DBSCAN algorithm for cLoops2 and named it blockDBSCAN, which starts with marking the smallest and biggest coordinates of PETs. It projects the shorter distance peaks in a 2D diagonal line and long interaction loops in space distance, removes the noisy points, and automatically calculates the cluster numbers. The blockDBSCAN classifies clusters into two groups: candidate peaks and candidate loops. It saves cLoops-specific input for analysis and can be reverted to the PETs-specific data. Here, for peak calling, Cao et al.[9] used blockDBSCAN that performs with a combination of different radii and minimum points in a cluster with $PETs > 1KB$. cLoops2 calculates the statistical significance in the nearby or same region using the Poisson test for all candidate peaks. It describes a candidate's peaks as overlapping with significant Poisson P-value having the highest RPKM value. In the end, cLoops2 rectifies all the Poisson P-values with Bonferroni correction. For calculating the significant peak, Cao et al.[9] took $P < 0.01$ by default. It calculates precision, sensitivity, and F1 scores for analyzing the result. For loop calls, cLoops2 uses blockDBSCAN with different radii and minimum points in a cluster. The blockDBSCAN provides candidates loops after performing numerous clustering rounds, and cLoops2 uses hypergeometric and Poisson tests like as cLoops[8] and an updated binomial test.

1.1.5 HiCCUPS

HiCCUPS [5] is a tool uses clustering to analyze every pixel in a Hi-C contact matrix and compare it to the pixels around it. It calculates the higher enriched pixels and

ensure that this higher enrichment is not from a large structural features. It calculates this enrichment compared with the lower left corner and this specific enrichment should be $\geq 50\%$ from the lower left corner enrichment. HiCCUPS also validate this enrichment with multiple hypothesis testing such as FDR $< 10\%$ and comparing with four neighborhood pixels- i) lower left, ii) left and right, iii) above and below and iv) a donut. This enrichment results in a regions of 5-20 contiguous interactions.

1.2 Probability-Based Tools

1.2.1 HiCExplorer

Wolff et al.[10] introduced HiCExplorer for loop detection in Hi-C data with a high loop detection percentage using low memory and multicore CPU architecture. HiCExplorer reduces the search space for achieving high throughput and has a good ability to distinguish true interaction and noise. They stated that HiCExplorer can detect almost all the true interactions considering relative genomic distance and noise regions using chromosome 1 on GM12878[10]. At first, HiCExplorer normalizes the the interaction height of every genomic distance by calculating observed over the expected matrix. For expected matrix calculation, the algorithm considers: i. non-zero contacts, ii. all contacts, and iii. different occurring ligation events which are similar to HOMER[11] normalization. Then HiCExplorer applies continuous negative binomial distribution, $\chi_{\bigtriangledown} \sim CNBD_{\bigtriangledown}(m_{\bigtriangledown}, n_{\bigtriangledown})\ \forall\ \bigtriangledown = |x - y|$ for candidate selection from the normalized data where $\bigtriangledown$ is the genomic distance. They modified the discrete negative binomial distribution function configuring with a modified gamma function[12, 13] for making it continuous. It calculates P-values with binomial density function and accepts candidates having a P-value less than a threshold considering only observed versus expected values. The algorithm considers the whole neighborhood of a^2 area, pools all candidates from a neighborhood, and divides them into peak and background region. After that, HiCExplorer again divides this neighborhood into vertical and horizontal regions similar to HiCCUPS technique[5]. The peak and neighborhood square size is defined by their peak width and window size. If a candidate satisfying $\bar{P} > \bar{B}$ condition where $\bar{P}$ is mean peak and $\bar{B}$ is mean background, they will be accepted as a loop.

1.2.2 FitHiC

Ay et al.[14] described FitHiC in 2014 by combining the random polymer looping effect and technical biases. FitHiC calculates statistical confidence in midrange intra-chromosomal contacts and requires contact count, number of distinct inter-chromosomal locus pairs, and total number of observed contact counts for this calculation. It considers null probability $= \frac{1}{\sigma}$, where σ = number of distinct inter-chromosomal locus pairs to calculate the probability by i. creating a specified equal occupancy bin by dividing the locus pairs, ii. calculating average contact count per locus pair, prior contact probability, and the average interaction distance over all locus pairs, and iii. applying a univariate spline to the derived points. From this point, FitHiC calculates the P-values of each bin and inter-chromosomal contact. It synthesizes the derived P-values to get a signal ranking for the observed contacts set. Finally, FitHiC corrects the combined P-values with the Benjamini Hochberg procedure and

produces a Q-value. This Q-value defines the statistical confidence of contacts and reveals the target loops.

1.2.3 FitHiC2

FitHiC2 is a versatile tool for analyzing intra-chromosome, inter-chromosome, and all-region interaction from Hi-C data. FitHiC2 is not limited to a minimum sequencing depth or any contact-maps resolution. It is capable of computing the statistical confidence of Hi-C datasets with random polymer looping effects and potential technical biases. Kaul et al.[15] introduced this improved version of FitHiC for detecting chromatin contacts without any parametric assumption. First, FitHiC2 reads user-defined ranged non-zero interaction counts and builds a genomic distance index and contact counts. It records specified ranges and places the contact counts into equal occupancy bins. Next, FitHiC2 reads the fragments file for computing equal occupancy bin by enumerating all possible fragment pairs for non-fixed-size data or looping through the possible fragment pairs for fixed-size data. This ultimately results in an average contact count and average distance. Then, FitHiC2 reads and stores bias value files according to their availability. After that, the algorithm applies null model univariate cubic spline function for each bin to the derived average distance over average contact count. It ensures that this spline is non-increasing by applying antitonic regression. Finally, FitHiC2 calculates the P-value. For this P-value, it reads each entry from the contact count file, calculates the prior contact probability and multiplies this with the bias value, and finally fits this corrected P-value into a binomial distribution. It outputs these derived P-values and corresponding Q-values by appending them to the contact counts file. If a user defines a specific number of pass parameters, then an extra spline fit process is performed into the null models.

1.2.4 HiC-ACT

Lagler et al.[16] created HiC-ACT with the aim to improve the chromatin interaction result by postprocessing the results from other methods (e.g., FitHiC2). These methods should consider interaction as independent to be postprocessed by HiC-ACT. For all sequencing depths, HiC-ACT shows a greater improvement in maintaining sensitivity and insignificant loss in precision. It uses aggregated Cauchy combination test (ACT)[17] and uses non-negative weights P-values from linear combination transformation. Under null hypothesis, Liu and Xie[17] proved that ACT is a standard Cauchy distribution, and using Cauchy distribution, test statistic and P-value could be transformed by each other. For test statistic, it conditions the smoothing window using the HiCRep[18] method considering the resolution and uses Gaussian Kernel weight function. First, HiC-ACT gathers results from a standard peak caller without spatial dependency and requires identifiers of bin pair and P-values. Next, the algorithm sets the distance value depending on the resolution. Then it selects interest pairs considering a threshold greater than the P-value between two bins. Finally, HiC-ACT determines all pairs and calculates weights, test statistics, and P-value for derived bin pairs.

1.2.5 FitHiChIP

Bhattacharyya et al.[19] introduced the FitHiChIP method in 2019 for loop detection. FitHiChIP models non-uniform coverage and genomic distance scaling at the same time and has reproducibility. It calculates the contact probability within two steps: i. monotonic spline fitting[14] for modeling the contact probability decay with increasing genomic distance and ii. applying a regression on observed contact and the bias values. First, FitHiChIP sorts all the locus pairs and applies an equal occupancy binning across the total contacts. It finds the sum of contact counts and average contacts per locus pair. Next, the algorithm calculates a prior contact probability using these average contacts and finds the average interaction distance for all possible pairs. Finally, FitHiChIP uses a univariate spline filtering for expected over prior contact probability. For this spline fitting and equal occupancy binning, FitHiChIP applies a background model (e.g., peak to all locus pairs, peak-to-peak loops). It determines statistical significance without bias regression and with bias regression. The algorithm calculates the contact probability with binomial distribution[14] without applying the bias regression and uses the prior contact probability directly from the spline fit. From this procedure, FitHiChIP results in P-values and derives Q-values with the Benjamini–Hochberg procedure[20]. If it considers the bias (e.g., coverage, ICE), it applies bias regression in equal occupancy bin for getting bias values. It either applies HiChIP coverage to the mean coverage ratio or a matrix balancing method such as ICE[21] for bias values. FitHiChIP uses a linear regression model for all equal occupancy bins, calculates regression coefficients, and uses these in a smoothing spline. Then it determines expected contact counts and expected contact probability. Finally, it calculates the statistical significance with the binomial distribution.

1.2.6 GOTHiC

Mifsud et al.[22] introduced GOTHiC in 2017 considering known and unknown biases such as density of restriction sites, cleavage efficiency, mappability and believed these biases had an independent effect in each pair. GOTHiC uses a binomial probabilistic model for achieving precision across the loop detection procedure. It determines the probability of given read pairs using random ligation and observed over expected ratio. First, within a specific distance (default $10KB$) and in the same fragment, GOTHiC applies filtering read pairs for removing self-legations, dangling ends, re-legations, and incomplete digestion products. Next, it calculates the spurious read pair probability using two-locus relative coverage and read pairs fraction. Then the algorithm derives probability of read pairs with binomial cumulative density and results in P-value. Finally, GOTHiC results in Q-value applying Benjamini-Hochberg multiple-testing and defines statistically significant interactions.

1.2.7 HiC-DC

Carty et al.[23] introduced HiC-DC in 2017. They used hurdle regression (zero truncated negative binomial regression)[24] for Hi-C contact count resulting in a null or background model. HiC-DC can handle systematic biases such as GC content and mappability. The algorithm takes Hi-C intra-chromosomal interactions contact map

as input where every bin has its associated covariates vector. First, HiC-DC takes a random variable that follows a zero-truncated negative binomial distribution. Next, it defines the regression model with negative binomial distribution having a dispersion parameter and negative binomial mean parameter. It considers GC content and mappability features while calculating negative binomial mean parameters and this is a log-linear function. Then, HiC-DC calculates a third-order B-spline by establishing a relation between contact significance and genomic distance. After this, it refits the model for increasing statistical power after removing the bin counts greater than 97.5% null distribution percentile. Finally, HiC-DC calculates the P-value for each bin and then retrieves the adjusted P-value with the Benjamini-Hochberg procedure. It also performs known genomic and epigenomic labelling. For labelling, it performs IDR for getting reproducible peaks of each dataset and creates a peaks catalog by merging the GRanges objects. HiC-DC uses this catalog to label the bins as promoter, exonic, intronic, and distal intergenic. While labelling, it retrieves the GRanges object from the catalog.

1.2.8 ZipHiC

ZipHiC is a HMM and Approximation Bayesian Computation (ABC)[25] based algorithm for identifying interaction in 2D space. Osuntoki et al.[26] introduced this algorithm in 2022. ZipHiC uses a mixture model with K-components mixture density. Here, zero-inflated Poisson (ZIP) distribution is the first component and Poisson distribution the second. The algorithm describes this mixture model with a latent variable, L_{mn}. They used three components in their experiment, but it can accept two or more components. They also assumed that noise follows ZIP distribution in most of the cases, and this noise could be corrected with a log function. ZipHiC uses the hidden Markov random field (HMRF) based on HMM for hidden components and implements Potts model[27] for spatially dependent data as its prior. It models the latent variables as a 2D HMRF, and this latent variable depends on the neighbors' status. It defines the neighboring as an influence sum and uses a partition function as normalization constant. For this normalization constant, ZipHiC uses the ABC model. It implements the Empirical Bayes approach and conventional Bayesian approach, and those are based on posterior distribution proportional to the product of the prior and likelihood.

1.2.9 NeoLoopFinder

In 2021, Wang et al.[28] published the NeoLoopFinder for detecting chromosome loops induced with structural variations (SVs) along with interchromosomal translocations, large deletions, and inversions. NeoLoopFinder uses a modified matrix balancing technique and can handle signal distortion, mappability, GC content, and restriction fragment size. It can draw heatmaps, genes, epigenomic loops and tracks. It takes the Hi-C contact matrix and SVs, and produces genome-wide CNV profile and segments, linked SV events chain, corrected Hi-C matrix, chromatin loops and enhancer-hijacking events. They developed HMM-based CNV segmentation module with a better performance. NeoLoopFinder starts with CNV profiles and segments inference in the Hi-C contact matrix, and goes through matrix balancing process. It uses HiNT[29] and

HiCnv[30] for CNV profile computation. In the next step, sub-matrices are flipped or rotated according to breakpoints type and orientation. They initiated a graph-based algorithm for correctly combining the order of each SV that mitigates the complexity of SVs. They developed a linear regression model for balancing signals and bringing them into a similar range caused by the allelic effect. After this, NeoLoopFinder identifies loops, and starts with integrating Peakachu[31] and includes a pre-trained model. It trains this model considering resolutions, window sizes, and in-situ and dilution protocols.

1.2.10 HMRF Bayesian caller

Zheng Xu et al.[32] introduced this algorithm in 2015 with a basis of finding intra-domain interactions. They showed this algorithm has great power to find peaks accurately along with reproducibility and statistical power. This algorithm uses Hi-C generated contact frequency matrix between fragments pairs where they considered observed $= O_{mn}$ and expected $= E_{mn}$ contact frequencies between m and n fragment, and $1 \leq m < n \leq T$ and $T =$ total number of fragments. It has a binary indicator variable, X_{mn} and this has two values. Value 1 represents a peak and -1 represents a non-peak. Zheng Xu et al. conjectured that observed contact frequencies use a negative binomial distribution, $O_{mn} \sim NBD(M_{mn}, \alpha)$ where α is over-dispersion parameter, M_{mn} is mean of O_{mn} with a variance $M_{mn} + M_{mn}^2/\alpha$. They also believed that there is an effect of the status of X_{mn}, and this is derived with a log function of O_{mn} and E_{mn} where they used FitHiC or ICE[21] methods. This algorithm uses the HMRF model for identifying local spatial dependency and finds out all 2D peaks. It stretches the HMRF model from 1D space to 2D space maintaining the concept of the original HMM or Bayesian hidden Ising model. It follows Ising prior[33] model for binary indicator variable, X_{mn} which depends on four neighbor fragment pairs, $\{(i-1,j),(i+1,j),(i,j-1),(i,j+1)\}$. This algorithm measures the cluster level among binary variables for inverse temperature parameter[34], and this parameter's large value indicates a tightly clustered configuration of X_{mn}, and zero values indicate independent uniform prior. If there is no cluster, this algorithm calibrates the inverse temperature parameter close to zero without losing any power. This algorithm uses the Metropolis-Hastings algorithm for deriving all the parameters except the inverse temperature. It uses the Bayesian approach[35] for posterior distribution-based parameter inference. First, this algorithm specifies the prior and re-parameterizes by doing inversion of over-dispersion parameters, which provides convenience and computational efficiency. This algorithm employs weak prior with large variance by default, and there is little impact of priors in the final peak calls. It combines the binomial distribution mixture with conditional independence assumption and introduces the posterior probability function.

1.3 Classification-Based Tools

1.3.1 FIREcaller

In 2020, Crowley et al.[36] introduced FIREcaller to find the frequently interacting regions (FIREs) from Hi-C contact matrix. This is a classifier tool developed with

R and provides continuous FIRE scores, dichotomous FIREs, and super-FIREs. The input matrix is prepared by dividing the genome into non-overlapping consecutive b-sized bins[37]. At first, in each bin, FIREcaller determines the total local[37] cis-interactions number and local threshold value, and is mostly dominated by contact domain exert influences. This algorithm provides an option to set user-specific cis-interacting region's upper bound. Next, bins go through a filtering process that creates some systematic bias (e.g., GC content, mappability). It removes 0 GC content, mappability, and effective fragment length along with $> 25\%$ neighborhood bins, $< 90\%$ mappability, and overlapping bins. After that, FIREcaller performs within-sample normalization using HiCNormCis[37] that uses Poisson regression process to synthesize the systematic bias. It allows negative binomial regression model for bias removal and accepts normalized data as input. If this algorithm finds multiple datasets, it performs quantile normalization across samples of the normalized cis-interactions. Later, FIREcaller computes the Z-score from normalized cis-interactions, and P-values using the standard normal distribution and classify the bins as FIREs having P-value > 0.05. It can identify super-FIREs by adding all $-\ln(P - values)$ and ranks least to most interactive FIREs inspired by ROSE[38]. It identifies different FIREs according to fold change > 2 and P-value < 0.05 and visualizes FIREs and super-FIREs.

1.3.2 Peakachu

In 2020, Salameh et al.[31] published supervised learning-based loop detection tool, Peakachu. It is a random forest classification algorithm. According to Salameh et al., Peakachu achieved good results in their analysis and required less computational power. This algorithm gives the facility of tuning the pixel probability value that generates the higher or lower loop count. It requires $10KB$ binned Hi-C matrix and positive training set interaction list as input. It applies an 11×11 window for collecting interaction from the Hi-C matrix and discards those training lists whose Hi-C value is $< 10\%$. Peakachu derives the positive classes and defines negative classes by collecting nonzero centers and windows with random coordinates. It decomposes each sample into $2n + 1$ feature vector, where $n =$ sample feature space radius and appends P2LL with each feature vector. It uses the *sci-kit* tool and fits training datasets in random forests. It divides these datasets into separate test and training datasets for avoiding over-fitting. After this, Peakachu derives the optimal hyperparameters and uses Matthew's Correlation Coefficient for picking the best model. It calculates a score of each feature vector and applies a greedy algorithm. This greedy algorithm first defines the anchor regions according to P-value and applies the DBSCAN algorithm. After running DBSCAN within any two connected regions, Peakachu counts the candidate pixels and finds peaks.

1.4 Computer Vision-Based Tools

1.4.1 Mustache

In the real world, it is difficult to determine an object without its scaler position. Ardakany et al.[39] introduced Mustache tools to overcome this problem. They presumed chromatin loops could be found in blob-shaped object interaction regions and

developed this tool followed by a Scale-space framework with multiple parameters such as genomic region, CTCF-binding, size, and regulatory elements. Mustache tries to identify regions of interactions with high statistical significance. It applies a local Z-normalization technique and re-scales interactions with a logarithm function. As a result, this algorithm gets a scale-space representation of normalized contact maps. Mustache convolves normalized contact map with Gaussian to get scale-space representation, which ultimately produces smoothed contact map and then computes difference-of-Gaussians (DoGs). This tool employs two space-space octaves and computes P-value for each pixel with Lapasian distribution on each DoGs scales. After calculating DoGs, Mustache looks for local maxima in 3D space; these maxima are considered candidate loops, and others are discarded. Then, candidate loops go through different types of filters to get high confidence and locally enriched loops. Finally, Mustache i. removes candidate loops that do not belong to at least two consecutive scales, ii. discovers connected components using 8-connectivity and removes those candidate loops in sparse regions, and iii. discards candidates having contact counts $< 2\times$ expected counts and applies Benjamin-Hochberg[20] for P-value correction.

1.4.2 SIP & SIPMeta

Rowley et al.[40] developed the Significant Interaction Peak caller (SIP) and SIPMeta for identifying and characterizing loops. These two tools are platform independent and have efficiency in terms of time and memory consumption. They are not biased with noise and sequence depth. The SIP method reclaims raw Hi-C signals from the *.hic* (Juicer[41]) file, user-defined resolution, and normalization technique. It uses the sliding window technique for genome analysis, and user defines resolution and matrix size, which leads to the size of the window. It creates images using observed-expected value and applies a distance-normalization formula for calculating the central loop value. This mainly uses an image processing algorithm for candidate loop listing and applies filters to this list. First, SIP applies Gaussian blurring for making the signals smooth and then applies contrast enhancement[42] method such as White top-hat. Lastly, SIP applies a minimization and maximization filter[42] and homogenizes the background. These processes provide corrected images, and with these images, this algorithm creates a candidate loop list with the maximum detection algorithm offered by ImageJ[42]. These loops go through several filters before image processing (e.g., eliminating pixels having insufficient data, removal of pixels that do not have an increasing interaction). SIP filters the candidates' loops considering various parameters (e.g., KR value, Poisson CDF function, PA score). SIPMeta first infers bin size from loop file or creates image using the *.bedpe* file from the SIP procedure or user can specify the *.hic* file. After this step, SIPMeta tests all signals within a specified distance, calculates the APA[5] score, and produces an average matrix. With this matrix, SIPMeta generates square and bullseye plots using *bullseye.py*. In bullseye plots, SIPMeta has a ring segment representing $4 \times N$ bins where N is Manhattan distance and these rings' Z-score is calculated separately along with ADA score having Z-score > 1.

1.4.3 Chromosight

Matthey et al.[43] introduced Chromosight to detect all types of patterns in various genome-wide contact maps such as hairpin-like configuration in bacteria and yeast. They developed Chromosight based on CV having higher sensitivity in synthesis data and working well with data from different protocols without prior training data. Before going to the detection algorithm, it uses the ICE[21] algorithm for balancing the whole matrix, and this accounts for the associated biases. Then Chromosight divides each pixel with the mean of its diagonal and calculates observed over expected contact ratio. It discards the contacts that are above the user-defined threshold distance. Next, Chromosight calculates the Pearson coefficients with a Convolution algorithm. This algorithm convolves the template over the contact map and calculates the correlation coefficients. Finally, Chromosight separates the high correlation foci by i. discarding the correlation coefficient images less than a correlation coefficient threshold value, ii. generating adjacency graph with the remaining points, iii. labelling the nonzero pixels as contiguous foci with the Connected Component Labelling (CCL)[44], and with greater or equal to two pixels focus are kept, iv. detecting the patterns having the highest coefficient pixel and filters the overlapped or closed patterns, and v. sorting out candidate local maxima and filters out neighboring candidates.

1.4.4 DeepLoop

DeepLoop [45] is a deep learning based multidimensional tool which can predict chromatin interaction using low depth Hi-C heatmap. It can correct bias data and enables loop resolution. They introduced LoopDenoise, a convolutional autoencoder architecture having five convolutional layer. LoopDenoise has two encoding layer with, two decoding layer and one final layer having eight 13×13, eight 2×2 and one 13×13 filters respectively. They used the MaxPooling layer to pull up the main features and applied ReLU activation function. DeepLoop accepts the HiCorr heatmap as input and pass it to the LoopDenoise autoencoder architecture. DeepLoop applied in different Hi-C data sets such as micro-C and have a comparable convergence and could discover loops that are escaped for X-inactivation.

1.5 Pile-up Procedure-Based Tools

1.5.1 Coolpup.py

Sequencing depth is a major limitation of 3D genome organization analysis. Coolpup.py uses the pile-up procedure to mitigate this limitation. Flyamer et al.[46] introduced this tool available as a Python package. Coolpup.py removes technical biases or uninteresting biological signals. It has an isolated CLI for visualization which makes it an adaptable tool. First, Coolpup.py initializes a sparse matrix from input, defines regions, and tracks the number of summed-up regions during the process. It records the window coverage according to the specification and can rescale the window in the required shape. Next, Coolpup.py divides the total sum of the results by the total number of windows, applies coverage normalization, and removes the contact probability distance dependency according to specification. After this, it averages the

expected matrices for getting the normalized matrix or uses randomly shifted control regions. Finally, Coolpup.py divides this matrix with normalized matrix for distance effect removal.

2 Supplemental Tables

Table S1 Running time of every individual loop caller using primary GM12878 dataset.

Tools	5KB	10KB	100KB	250KB
Chromosight	387	174	2895	1018
cLoops	12260	12384	12551	12700
cLoops2	162217	153192	142331	10159
FitHiC2	36048	24498	1819	348
FitHiChIP	44030	30693	2334	516
HiCCUPS	5003	2365	968	0
HiCExplorer	8408	1430	0	0
LASCA	68620	20355	1035	425
Mustache	4349	2094	2552	1121
Peakachu	5484	3211	449	182
SIP	6404	3207	400	289

Table S2 Running time of every individual loop caller using primary GM12878.

Tools	10KB (Full)	5KB (Full)	10KB (chr1)	5KB (chr1)	10KB (chr6)	5KB (chr6)
LASCA			1014	3218.8	571.7	1722.7
HiCExplorer			1602	4415	1198	3503
FitHiC2			1072	1464	764	1015
FitHiChIP	30693	44030				
Peakachu			673	1195	501	892
Mustache			254	507	176	334
Chromosight	6440	7322				
SIP	353	358				
cloops			1510		1196	
cloops2			2338		1030	
HiCCUPS			192	380	128	235

Table S3 Running time of every individual loop caller using KR normalized GM12878 dataset.

Tools	10KB (Full)	5KB (Full)	10KB (chr1)	5KB (chr1)	10KB (chr6)	5KB (chr6)
LASCA			931	3393.5	583.7	1694.5
HiCExplorer			378	1741	318	1646
FitHiC2			2514	3619	1709	2423
FitHiChIP	32792	45553				
Peakachu			270	431	189	280
Mustache			238	533	195	347
Chromosight	160	333				
SIP	3515	6338				
HiCCUPS			196	393	128	239

Table S4 Running time of every individual loop caller using replicate GM12878 dataset.

Tools	10KB (Full)	5KB (Full)	10KB (chr1)	5KB (chr1)	10KB (chr6)	5KB (chr6)
LASCA			955.6	3408	583.6	1683.8
HiCExplorer			526	2589	338	1690
FitHiC2			2235	3011	2532	3158
FitHiChIP	26565	41551				
Peakachu			254	378	183	252
Mustache			190	438	123	270
Chromosight	162	325				
SIP	3755	6407				
cloops			80137		53667	
cloops2			196596		83716	
HiCCUPS			194	392	129	241

Table S5 Memory usages of every individual loop caller using primary GM12878 dataset.

Tools	5KB	10KB	100KB	250KB
Chromosight	54003.29	70644.33	70221.00	70221.00
cLoops	54428.33	45458.56	35713.53	29582.71
cLoops2	29721.98	28979.37	27360.96	24291.78
FitHiC2	22031.61	22543.88	20945.76	21241.33
FitHiChIP	23438.20	24898.30	22435.20	26816.63
HiCCUPS	59731.24	56224.00	58781.00	0.00
HiCExplorer	51596.29	31851.26	0.00	0.00
LASCA	55923.47	35017.87	30935.50	30399.75
Mustache	29676.12	32938.98	24922.34	23561.98
Peakachu	24676.96	24643.07	26592.50	28767.50
SIP	32137.80	31298.50	29208.14	26269.83

Table S6 Loop count of every individual loop caller using primary GM12878 dataset.

Tools	10KB (chr1)	5KB (chr1)	10KB (chr6)	5KB (chr6)
LASCA	1332	2801	962	1814
HiCExplorer	1970	2334	1284	1663
FitHiC2	63150	66440	46812	50260
FitHiChIP	3693	770	3175	952
Peakachu	2312	1953	1705	1633
Mustache	2018	3749	1570	2490
Chromosight	951	1426	614	866
SIP	249	1442	310	1040
cloops	154		35	
cloops2	76		62	
HiCCUPS	3890	3302	2367	1878

Table S7 Loop count of every individual loop caller using KR normalized GM12878 dataset.

Tools	10KB (chr1)	5KB (chr1)	10KB (chr6)	5KB (chr6)
LASCA	1503	3146	1077	2095
HiCExplorer	1471	1757	1071	1319
FitHiC2	63150	66440	46812	50260
FitHiChIP	3678	769	3165	951
Peakachu	7607	3867	2668	1027
Mustache	5844	2809	3900	1946
Chromosight	958	1414	625	888
SIP	315	747	413	589
HiCCUPS	3890	3302	2367	1878

Table S8 Loop count of every individual loop caller using replicate GM12878 dataset.

Tools	10KB (chr1)	5KB (chr1)	10KB (chr6)	5KB (chr6)
LASCA	1543	3249	1045	2197
HiCExplorer	1732	1923	1119	1344
FitHiC2	50638	67576	36962	53178
FitHiChIP	1864	225	1669	380
Peakachu	2638	905	855	281
Mustache	4805	2237	3393	1563
Chromosight	928	1184	607	788
SIP	361	608	421	403
cloops	1658		1140	
cloops2	2492		1696	
HiCCUPS	2930	1945	1586	952

Table S9 APA score using GM12878 primary data.

Tools	5KB (chr1)	10KB (chr1)	5KB (chr6)	10KB (chr6)
LASCA	1	1.2	0.953	1.21
HiCExplorer	2.87	2.28	2.98	2.27
FitHiC2	179	83.1	28.1	18.6
FitHiChIP	2.52	1.75	4.89	1.89
Peakachu	1.33	1.3	1.34	1.55
Mustache	1.4	1.56	1.46	1.57
Chromosight	1.11	0.904	1.13	0.91
SIP	3.4	3.59	3.7	3.33
cloops	2.73		5.12	
cloops2	3.61		3.98	
HiCCUPs	2.04	1.81	2.1	1.82

Table S10 APA score using GM12878 KR normalized data.

Tools	5KB (chr1)	10KB (chr1)	5KB (chr6)	10KB (chr6)
LASCA	1.05	1.13	1.1	1.3
HiCExplorer	3.45	1.72	1.67	1.27
FitHiC2	212	76.5	36.9	16.7
FitHiChIP	2.54	1.68	4.86	1.92
Peakachu	1.13	0.933	1.26	1.04
Mustache	1.45	1.39	1.52	1.44
Chromosight	1.18	0.903	1.19	0.902
SIP	2.7	3.03	2.72	2.77
HiCCUPs	2.08	1.78	2.12	1.86

Table S11 APA score using GM12878 replicate data.

Tools	5KB (chr1)	10KB (chr1)	5KB (chr6)	10KB (chr6)
LASCA	1.1	1.17	1.05	1.21
HiCExplorer	2.64	1.72	2.75	1.97
FitHiC2	174	88.6	67.8	23.4
FitHiChIP	2.8	1.91	9.43	2.34
Peakachu	1.24	1.05	1.28	1.15
Mustache	1.36	1.37	1.39	1.4
Chromosight	1.12	0.907	1.12	0.849
SIP	2.36	2.57	2.65	2.45
cloops	2.28		2.52	
cloops2	2.25		2.11	
HiCCUPs	2.08	1.72	2.19	1.82

3 Supplemental Figures

3.1 Loop Count

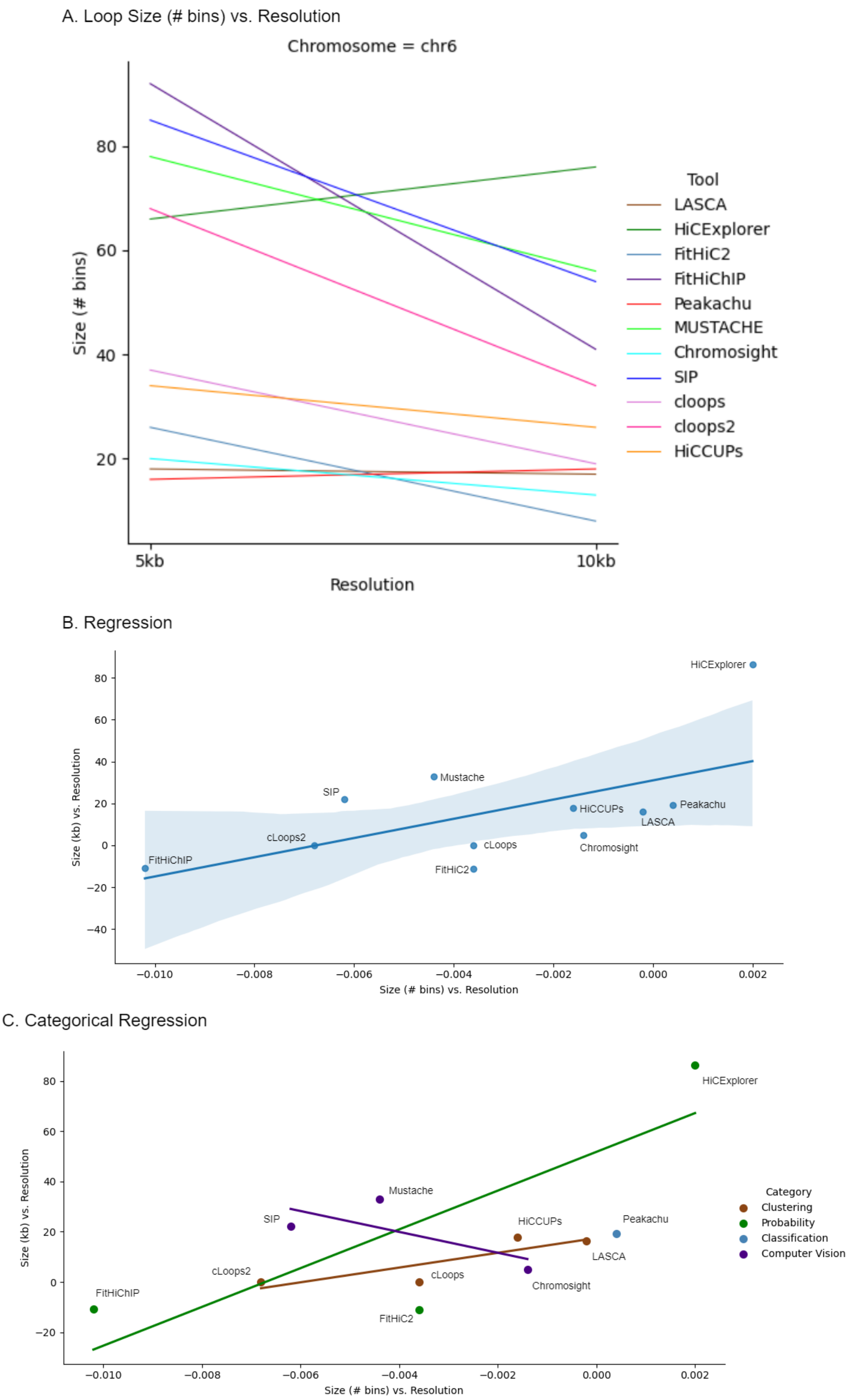

Figure S1 A. Illustration of chromatin loop size in terms of number of mean bins using primary, replicate and normalized GM12878 chromosome 6 at 5KB and 10KB. Regression plots of chromatin loop caller tools calculating the slope of resolution and loop size (KB) versus loop size (# bins) and resolution where B. overall regression and C. categorical regression plot.

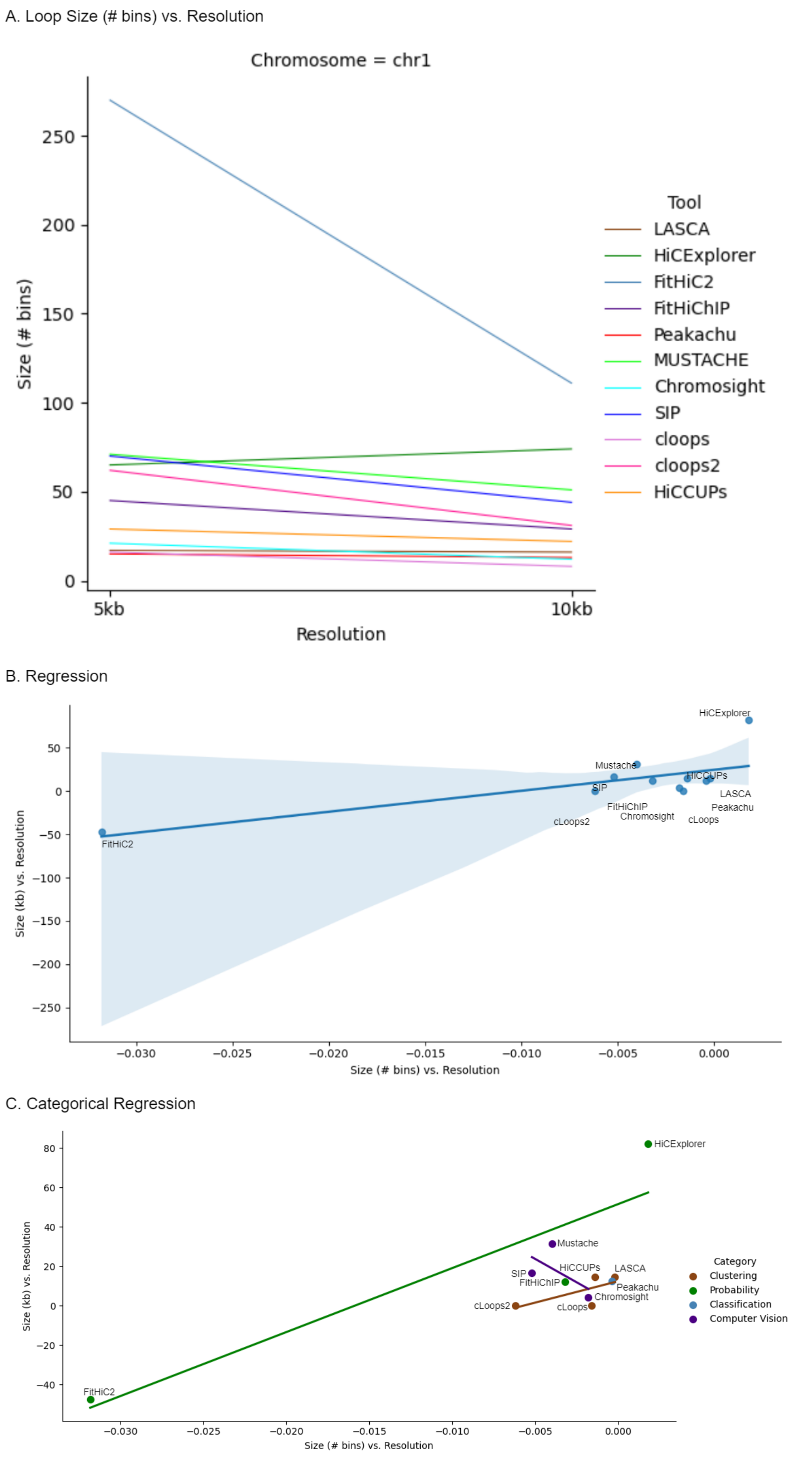

Figure S2 A. Illustration of chromatin loop size in terms of number of mean bins using primary, replicate and normalized GM12878 chromosome 1 at 5KB and 10KB. Regression plots of chromatin loop caller tools calculating the slope of resolution and loop size (KB) versus loop size (# bins) and resolution where B. overall regression and C. categorical regression plot.

3.2 Overlap Analysis

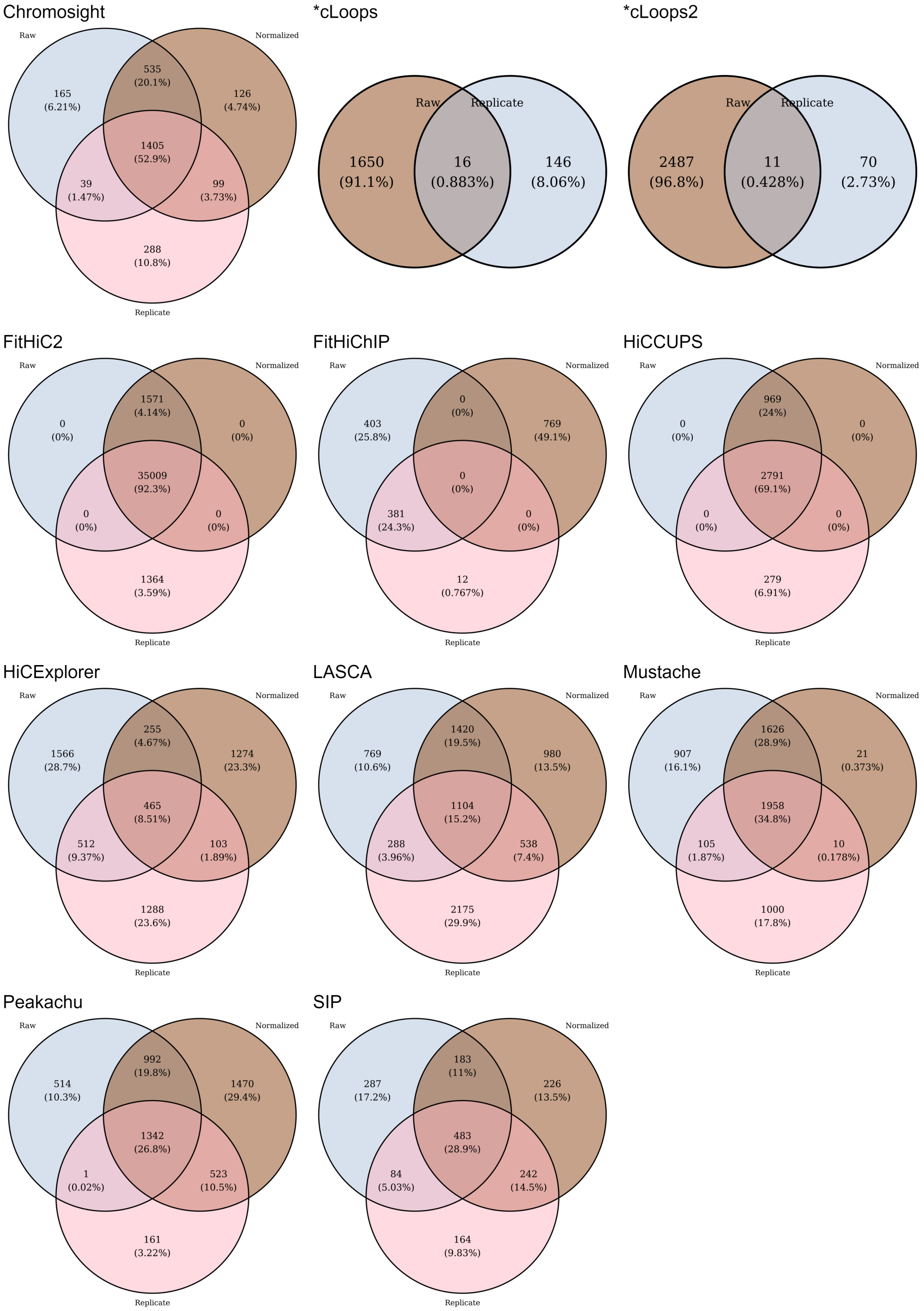

Figure S3 Overlap across three (primary, replicate and KR normalized) dataset of GM12878 using chromosome 1 at 5kb resolution. As FitHiC2 predicts a highest amount of loops, it overlap ratio is highest. Considering other tools, HiCCUPS scores 69.1%, Chromosight 52.9%, Mustache 34.8% and FitHiChIP does not overlap any loops. Other tools overlaps below 30% loops. cLoops and cLoops overlaps near 0% loops and HiCExplorer scores 8.51%.

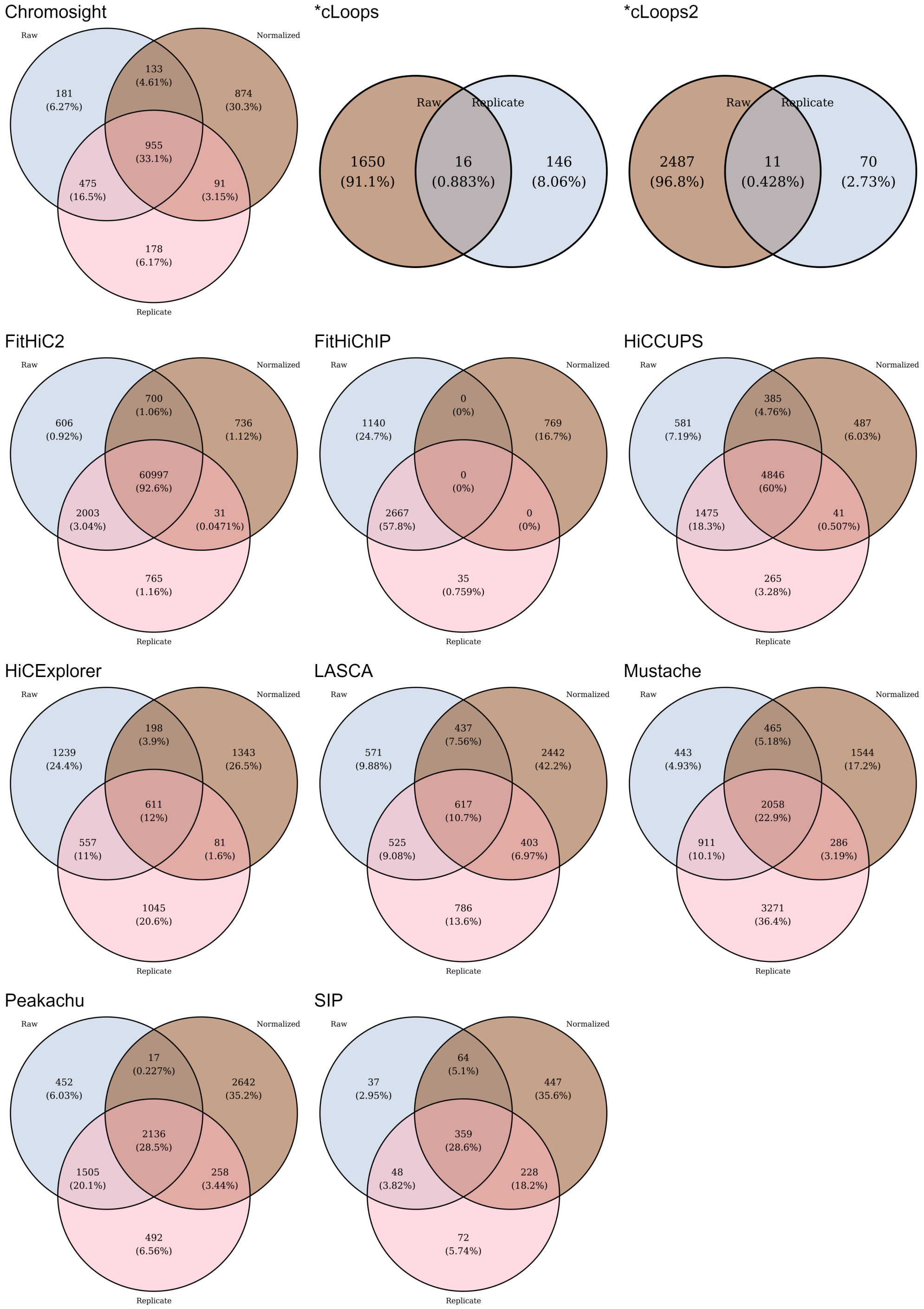

Figure S4 Overlap across three (primary, replicate and KR normalized) dataset of GM12878 using chromosome 1 at 10kb resolution. FitHiC2 overlaps 92.6% loops across three datasets. cLoops and cLoops2 scores near to zero and FitHiChIP does not overlap any loops. HiCExplorer, LASCA, Mustache, Peakachu and SIP overlap below 30% loops. HiCCUPS overlaps 60% loops and Chromosight scores around 33%.

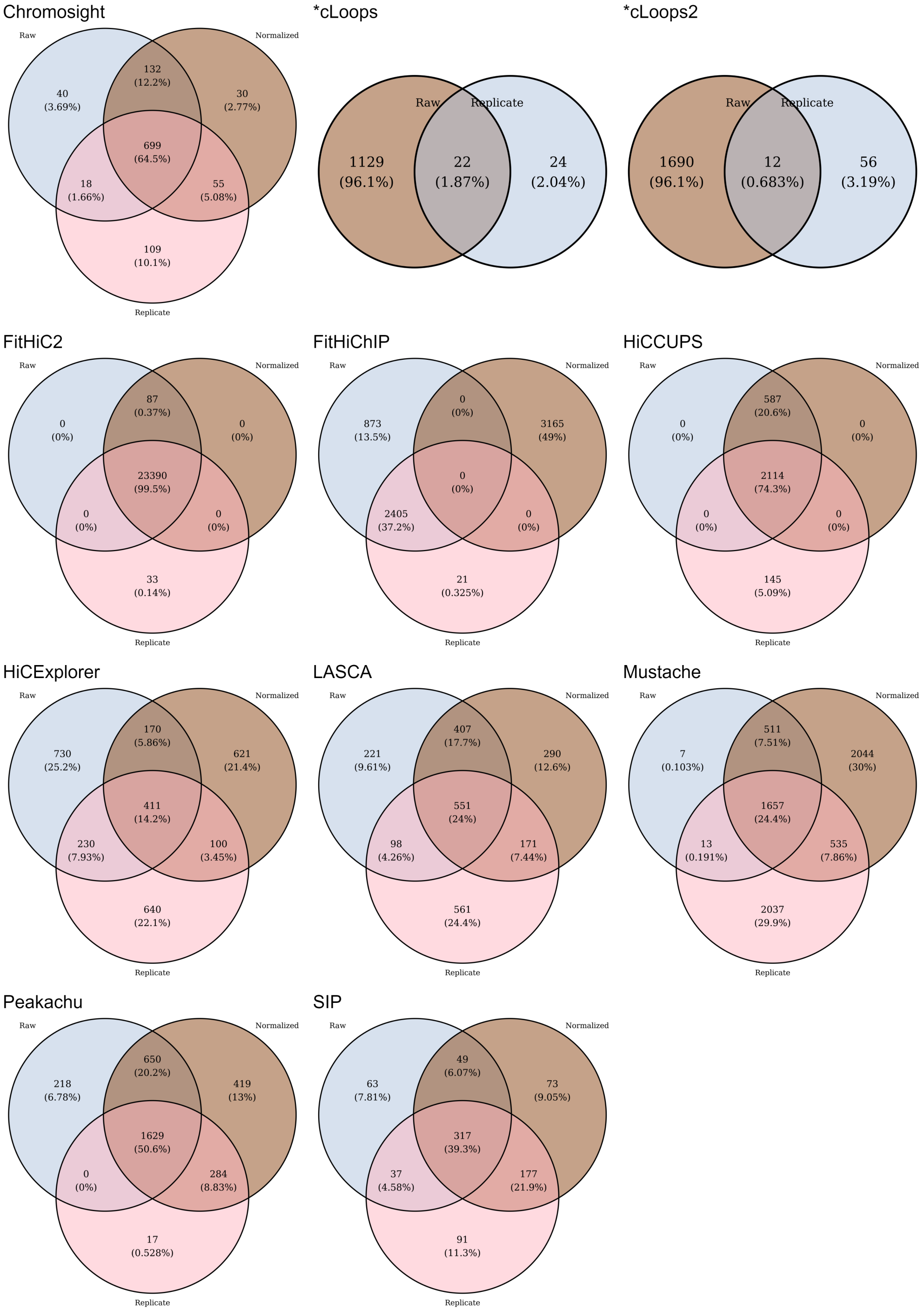

Figure S5 Overlap across three (primary, replicate and KR normalized) dataset of GM12878 using chromosome 6 at 10kb resolution. FitHiC2 overlaps allmost all the loops, and Chromosight overlaps 64.5%, Peakachu overlaps 50.6% and HiCCUPS overlaps 74.3% loops. SIP scores around 40%, and HiCExplorer, LASCA and Mustache overlaps below 30% loops. cLoops and cLoops2 overlaps around 1% loops across primary and replicate data.

3.3 Peak Analysis

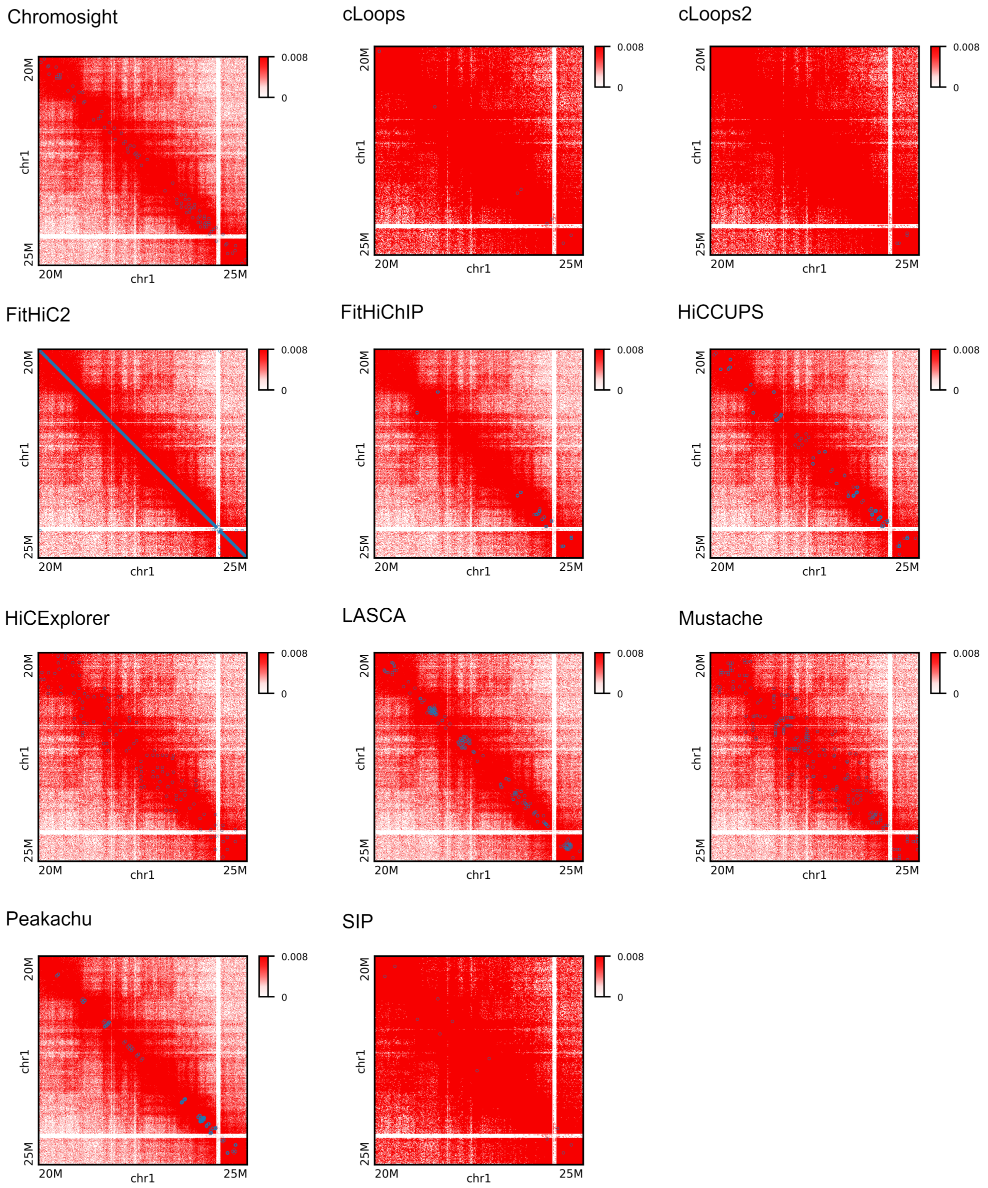

Figure S6 Peak plots for chromosome 1 at 5KB using primary GM12878 data from 20M to 25M genomic range. FitHiC2 marks peaks through the diagonal from left to right creating a diagonal strong straight line. Chromosight, HiCCUPS, HiCExplorer, LASCA, Mustache, and Peakachu mark significant number of the peaks in this region. cLoops, cLoops2, FitHiChIP, and SIP finds least amount of peaks in this region.

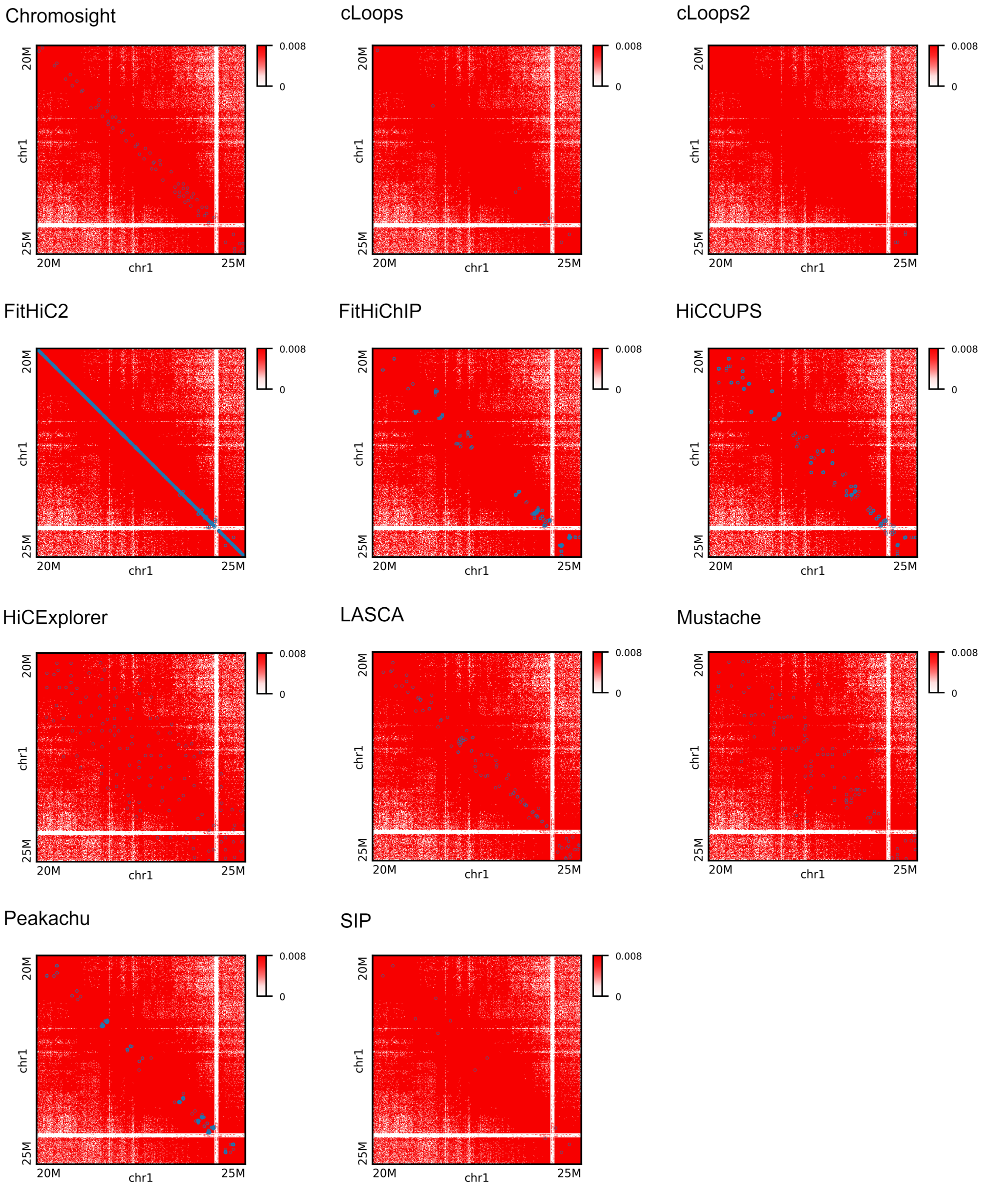

Figure S7 Peak plots for chromosome 1 at 10KB using primary GM12878 data from 20M to 25M genomic range. SIP, cLoops and cLoops2 finds the least amount of peaks whereas FitHiChIP, Chromosight, LASCA and Peakachu more dense compared to them. HiCExplorer and Mustache mark peaks covering a wide range of area, and HiCCUPS points significant amount of peaks. FitHiChIP marks more peaks compared to 5KB data. HiCExplorer marks peaks within a large diagonal region in the heatmap compared to 5KB data. FitHiC2 marks peaks through the diagonal from left to right creating a diagonal strong straight line.

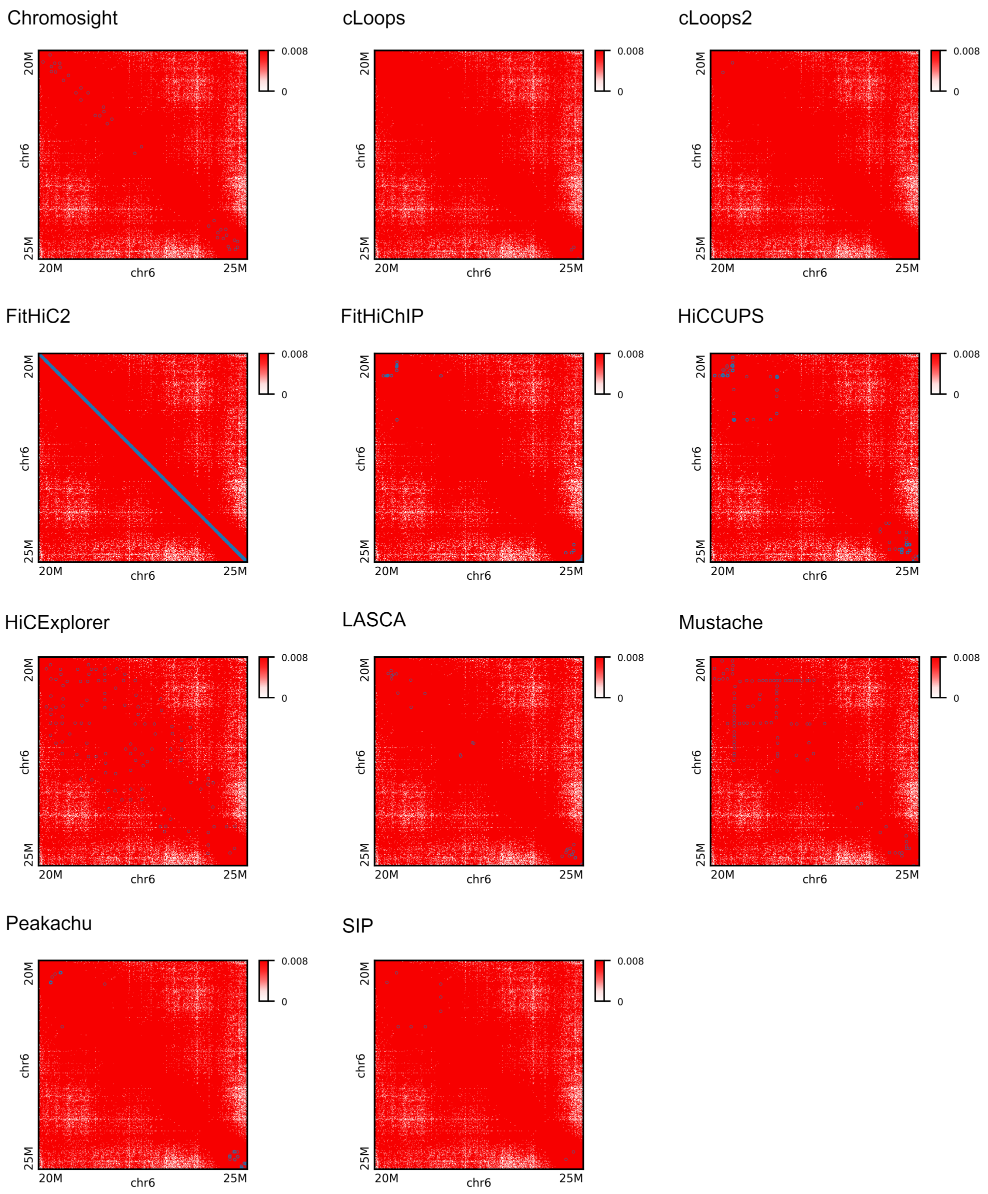

Figure S8 Peak plots for chromosome 6 at 10KB using primary GM12878 data from 20M to 25M genomic range. cLoops and cLoops2 marks almost zero amount of peaks. FitHiC2 marks peaks through the diagonal from left to right creating a diagonal strong straight line. HiCCUPS, Mustache and SIP mark peaks in the upper left diagonal corner. HiCExplorer and Mustache covers a wide area predicting peaks. HiCExplorer, Mustache, Chromosighr, and HiCCUPS mark more peaks compared to other tools at this region.

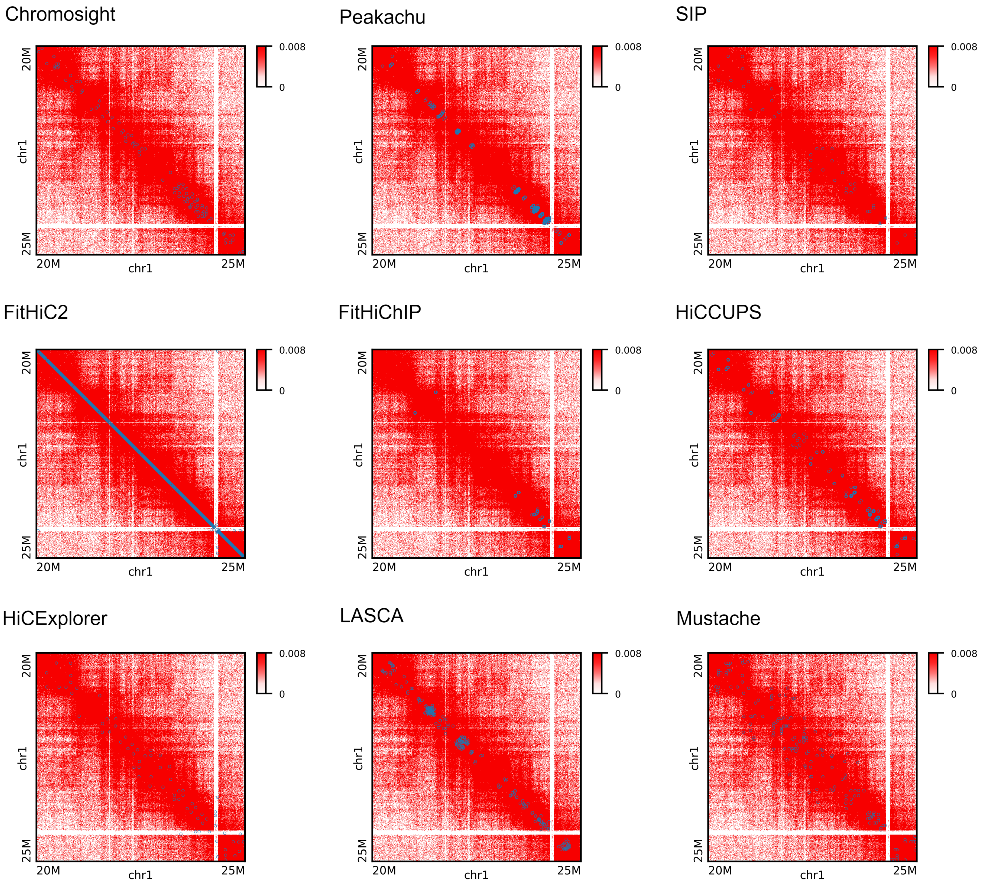

Figure S9 Peak plots for chromosome 1 at 5KB using KR normalized GM12878 data from 20M to 25M genomic range. FitHiChIP marks less peaks compared to other tools, and LASCA and Peakachu marks saturated loops at some points. FitHiC2 marks peaks through the diagonal from left to right creating a diagonal strong straight line. Mustache, LASCA, HiCCUPS, Peakachu, Chromosight and HiCExplorer marks a visible amount of peaks. Mustache marks peaks within a wide range of diameter from the left to right diagonal line.

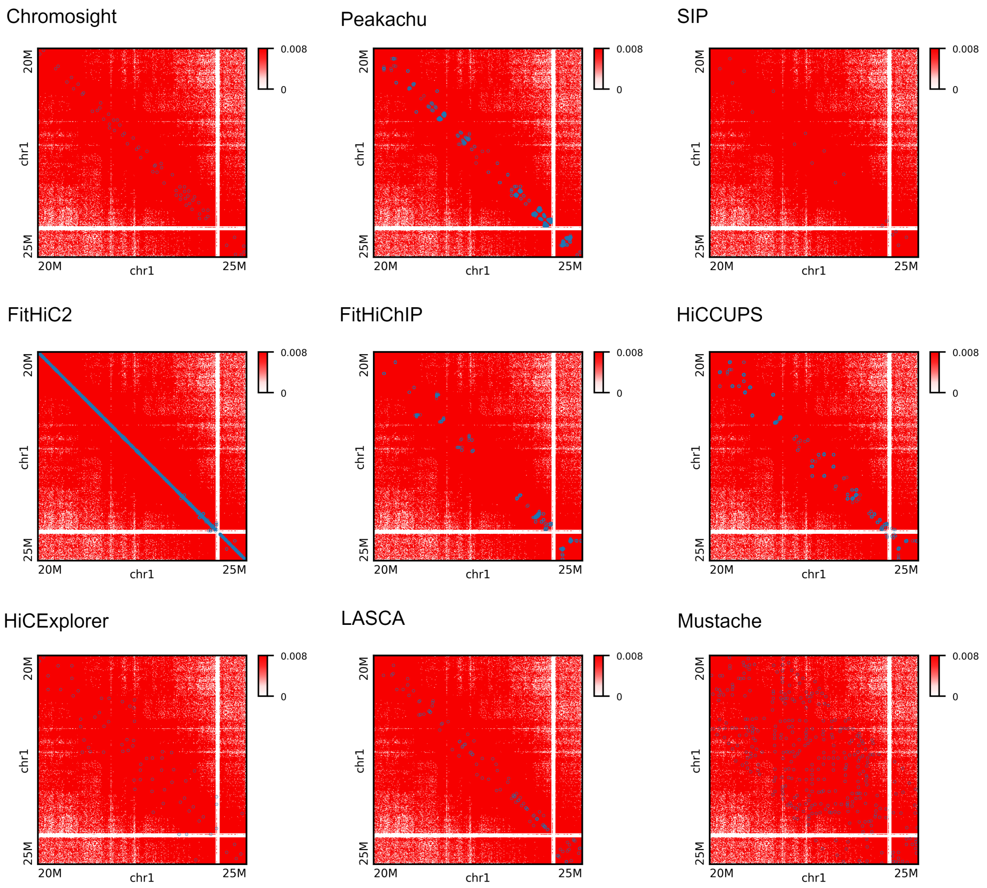

Figure S10 Peak plots for chromosome 1 at 10KB using KR normalized GM12878 data from 20M to 25M genomic range. Mustache marks peaks over a diagonal region covering most of the region. SIP marks less peaks, and Peakachu, FitHiChIP, HiCCUPS and LASCA create some saturated peaks points.

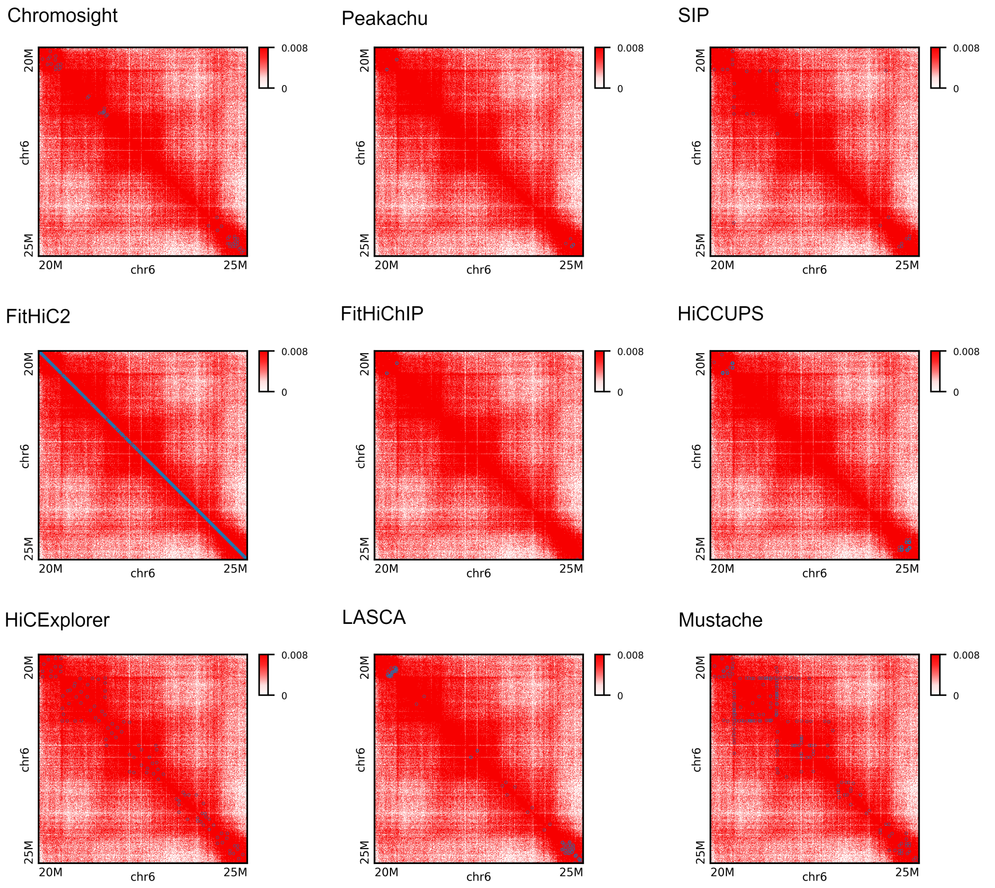

Figure S11 Peak plots for chromosome 6 at 5KB using KR normalized GM12878 data from 20M to 25M genomic range. Mustache and HiCExplorer marks more loops compared to other tools.

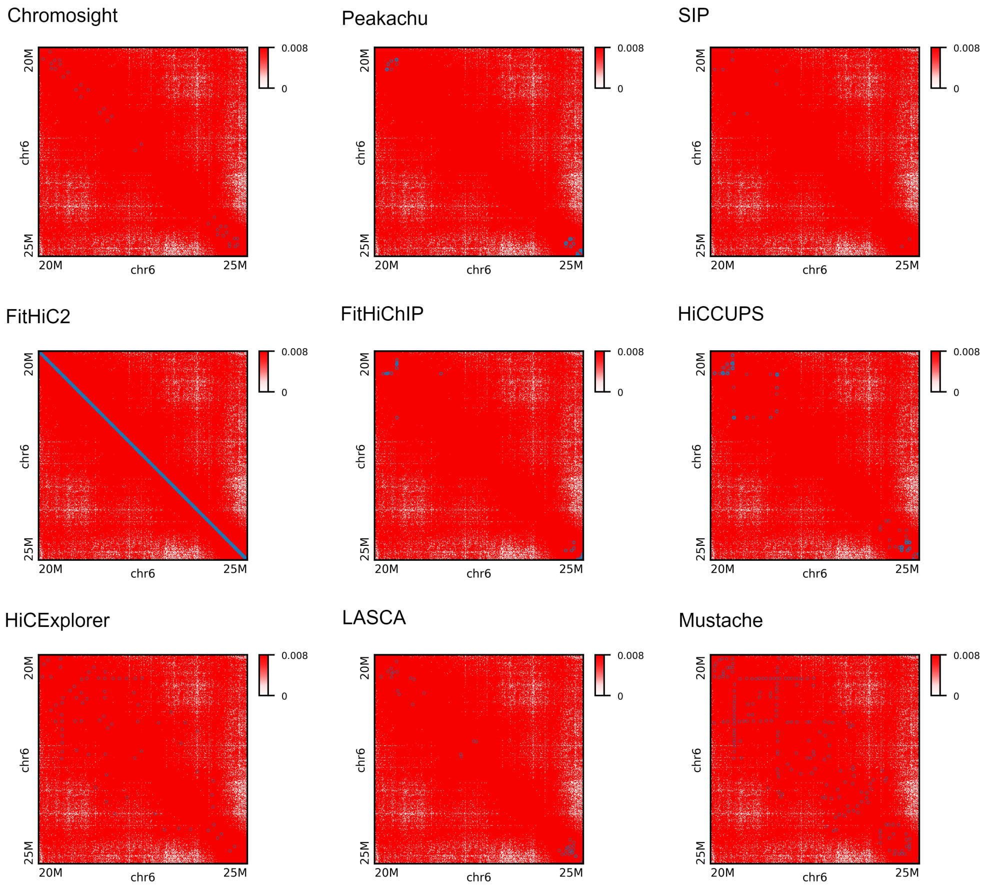

Figure S12 Peak plots for chromosome 6 at 10KB using KR normalized GM12878 data from 20M to 25M genomic range. Mustache continues the same pattern as 5KB with some less peaks marked where HiCExplorer covers a large diagonal area. HiCCUPS marks more loops compared to 5KB data.

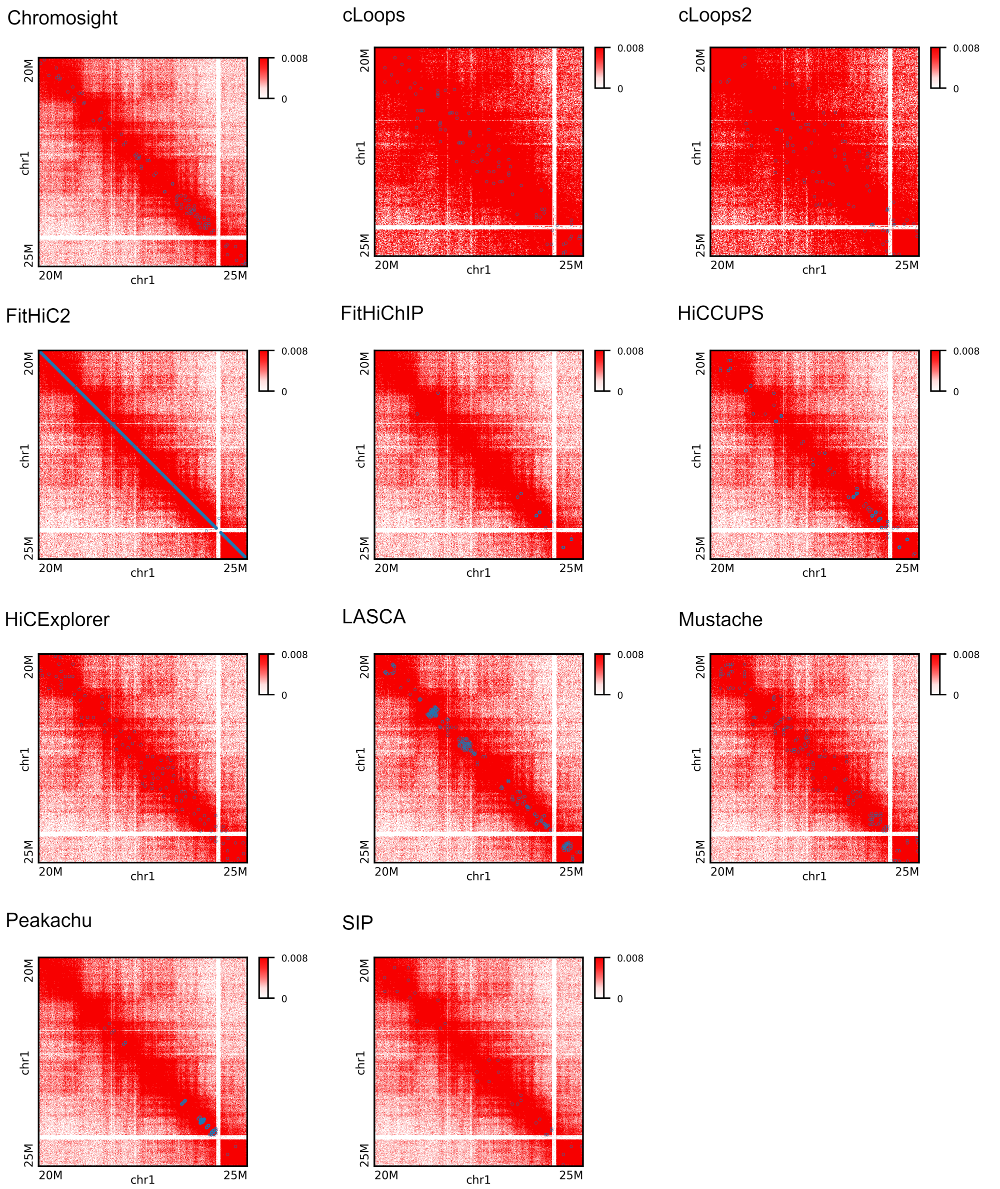

Figure S13 Peak plots for chromosome 1 at 5KB using replicate GM12878 data from 20M to 25M genomic range. FitHiChIP, Peakachu, and SIP mark the least amount of peaks at this region.

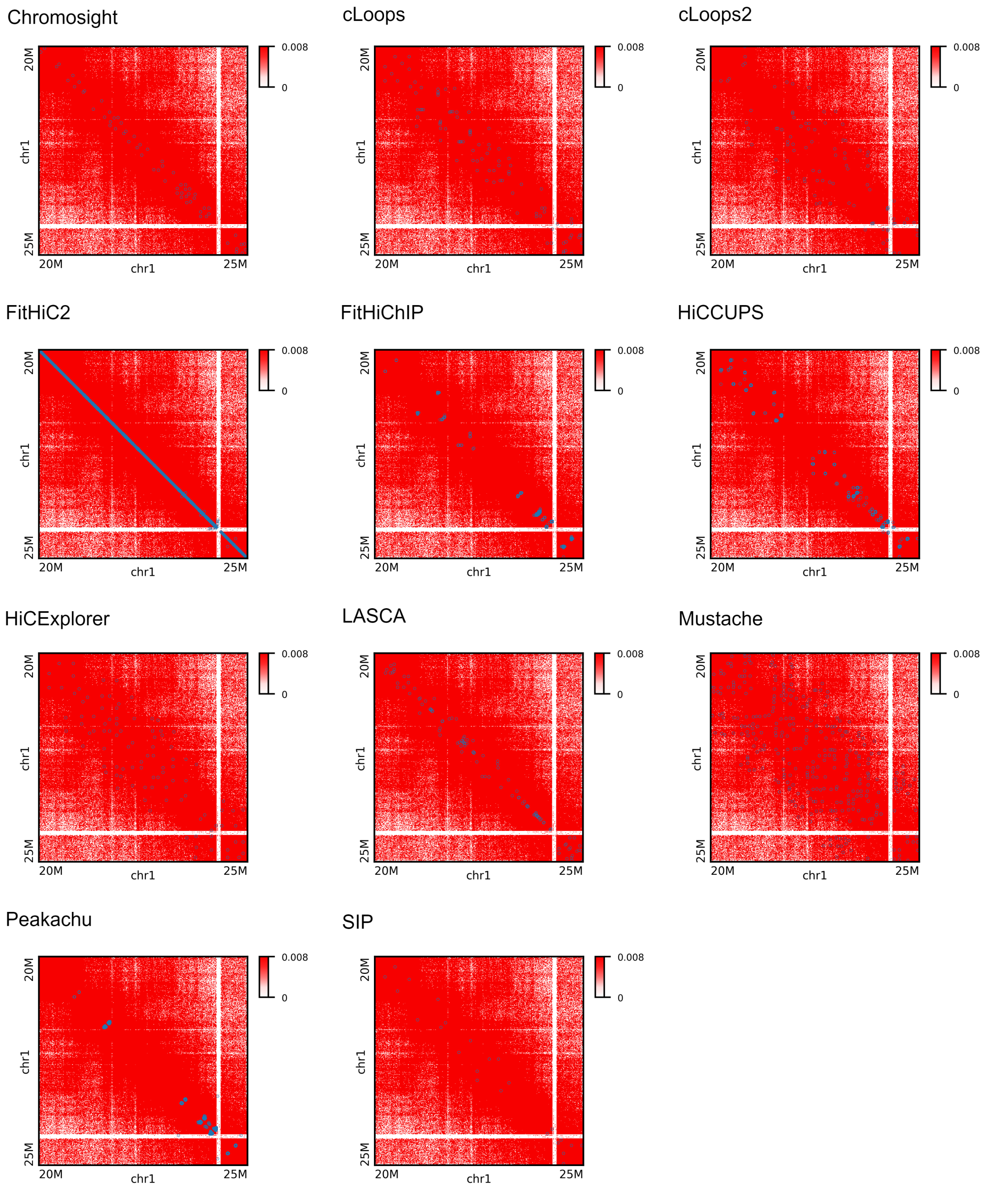

Figure S14 Peak plots for chromosome 1 at 10KB using replicate GM12878 data from 20M to 25M genomic range. Mustache and HiCExplorer covers a large area diagonally whrer as Chromosight, cLoops, and cLoops continue the same pattern as 5KB. FitHiChIP shows some saturated peaks at some points.

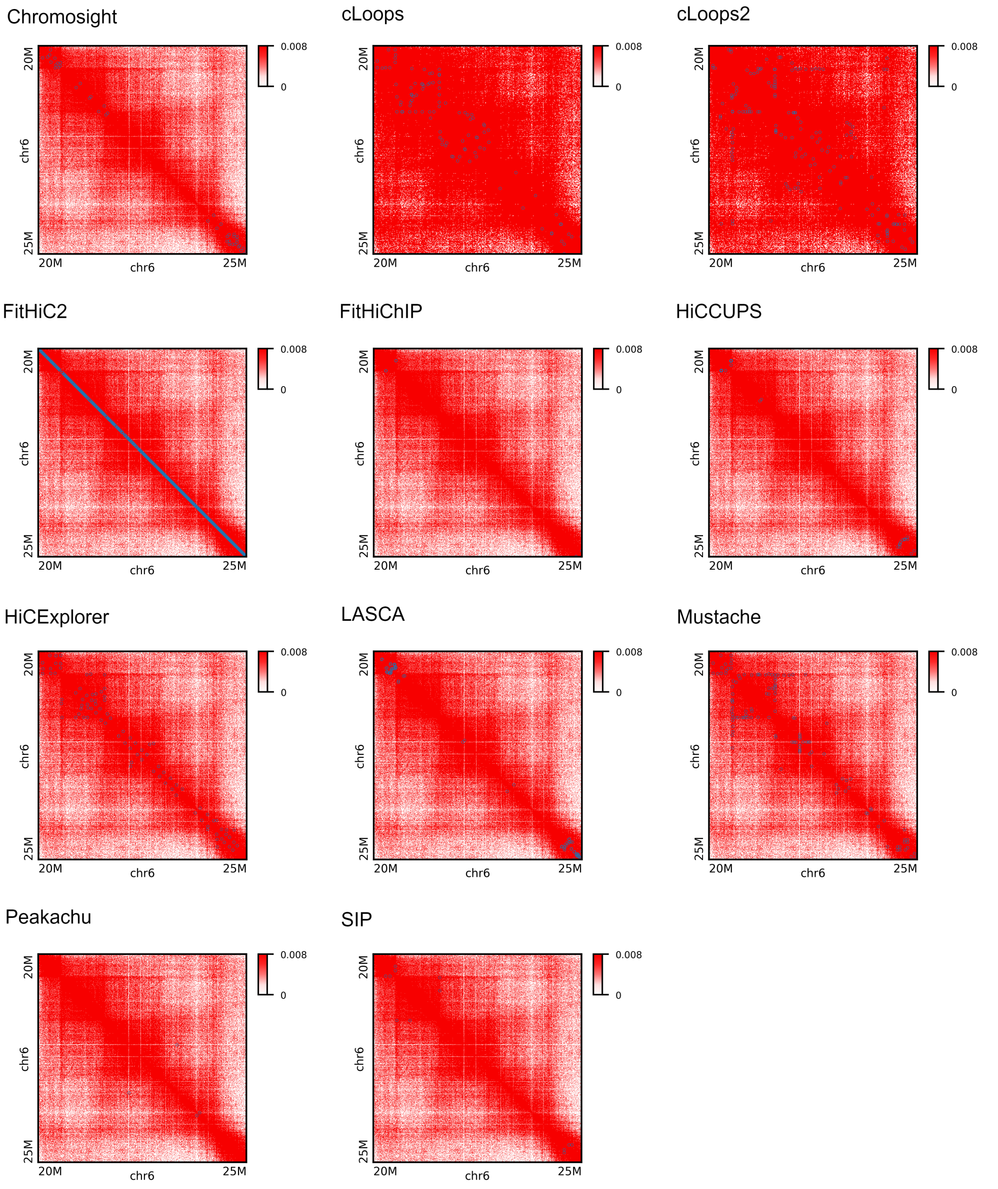

Figure S15 Peak plots for chromosome 6 at 5KB using replicate GM12878 data from 20M to 25M genomic range. cLoops, cLoops2, HiCExplorer, and Mustache marks most of the peaks at this regions. LASCA shows saturation points at corner sides.

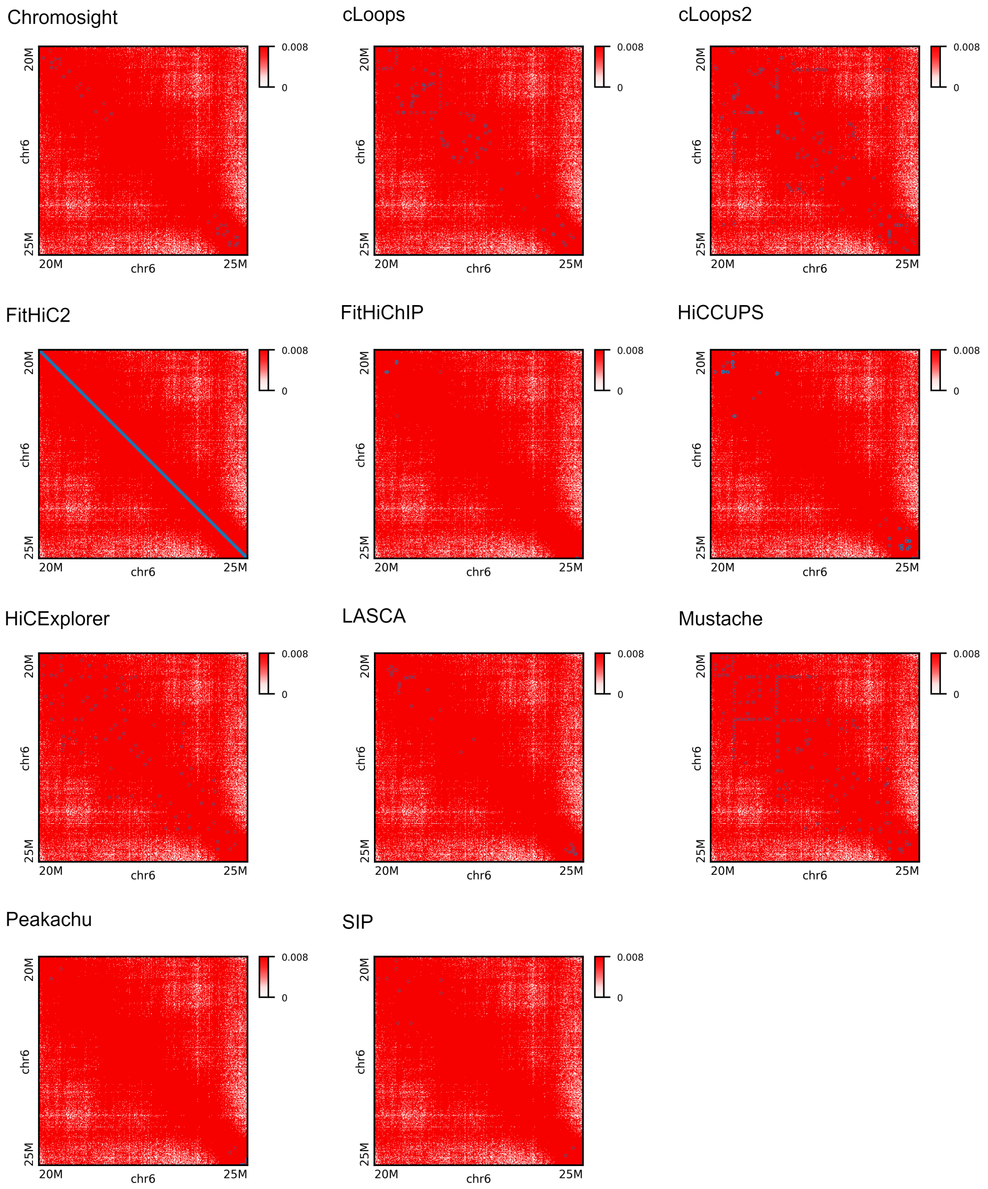

Figure S16 Peak plots for chromosome 6 at 10KB using replicate GM12878 data from 20M to 25M genomic range. HiCCUPS marks more peaks at 10KB compared to 5KB resolution data. HiCExplorer, Mustache, and cLoops2 cover a large area through the diagonal line. cLoops2 shows almost same peaks as in 5KB data.

3.4 APA Analysis

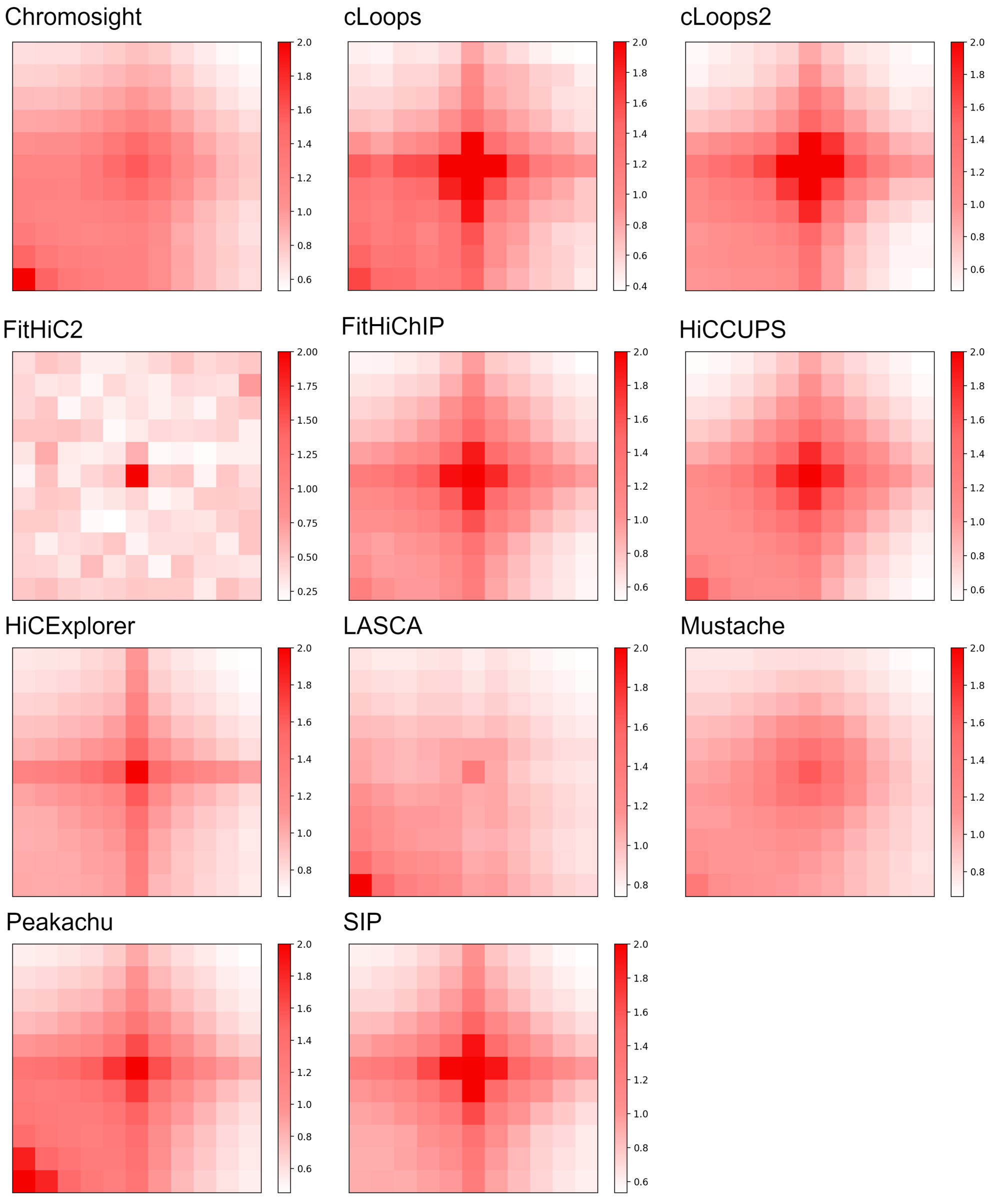

Figure S17 APA plot for chromosome 1 at 5KB using GM12878 primary data. FitHiC2 has the highest enrichment. cLoops, cLoops2, FitHiChIP, HiCCUPS, HiCExplorer, SIP have strong enrichment at the center. LASCA show strong enrichment at the lower left corner of the center.

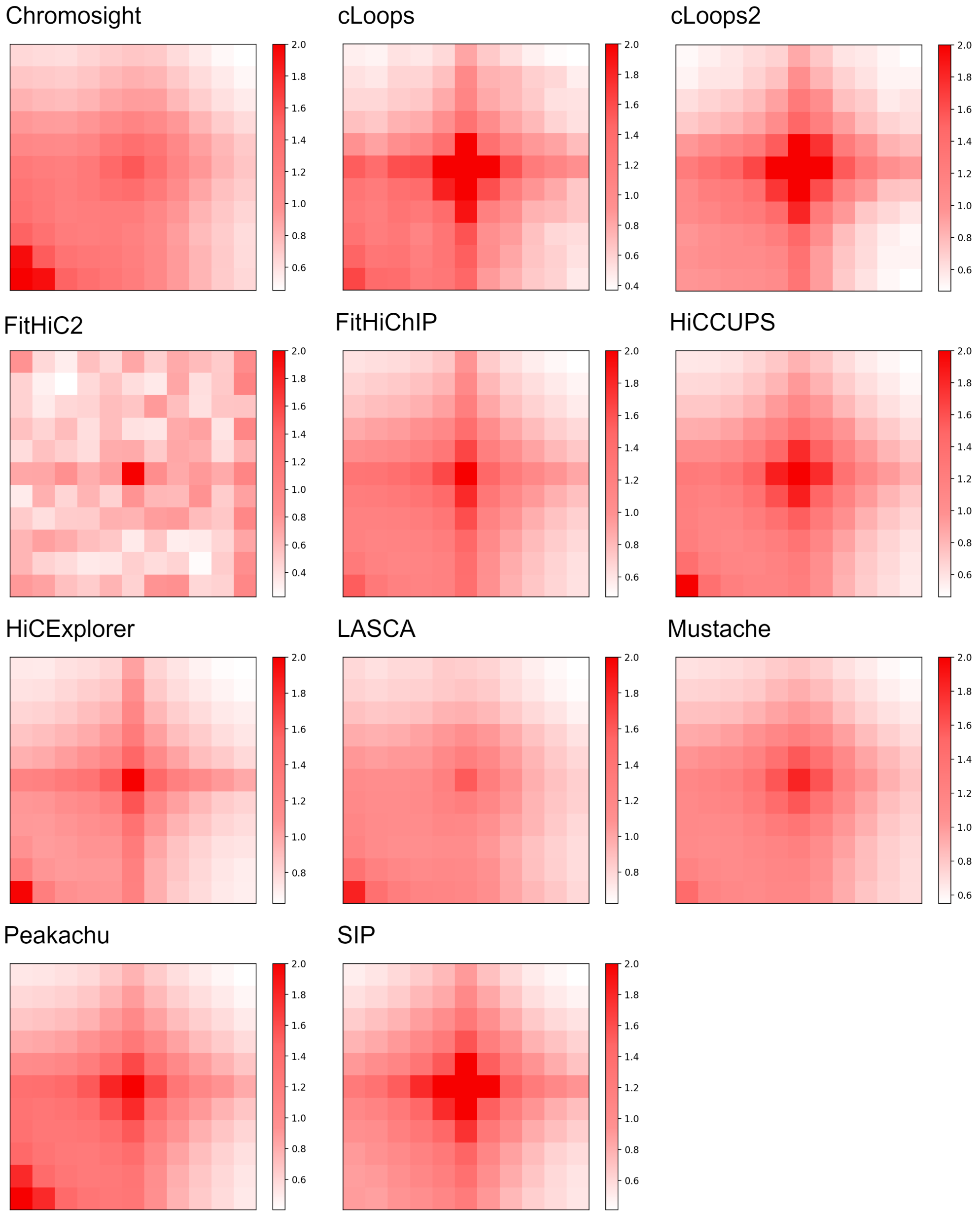

Figure S18 APA plot for chromosome 1 at 10KB using GM12878 primary data. FitHiC2 shows the highest APA score at the center, and cLoops, cLoops2, HiCExplorer, and SIP have strong enrichment at the center. Chromosight shows APA score less than 1 and other tools have enrichment greater than 1.

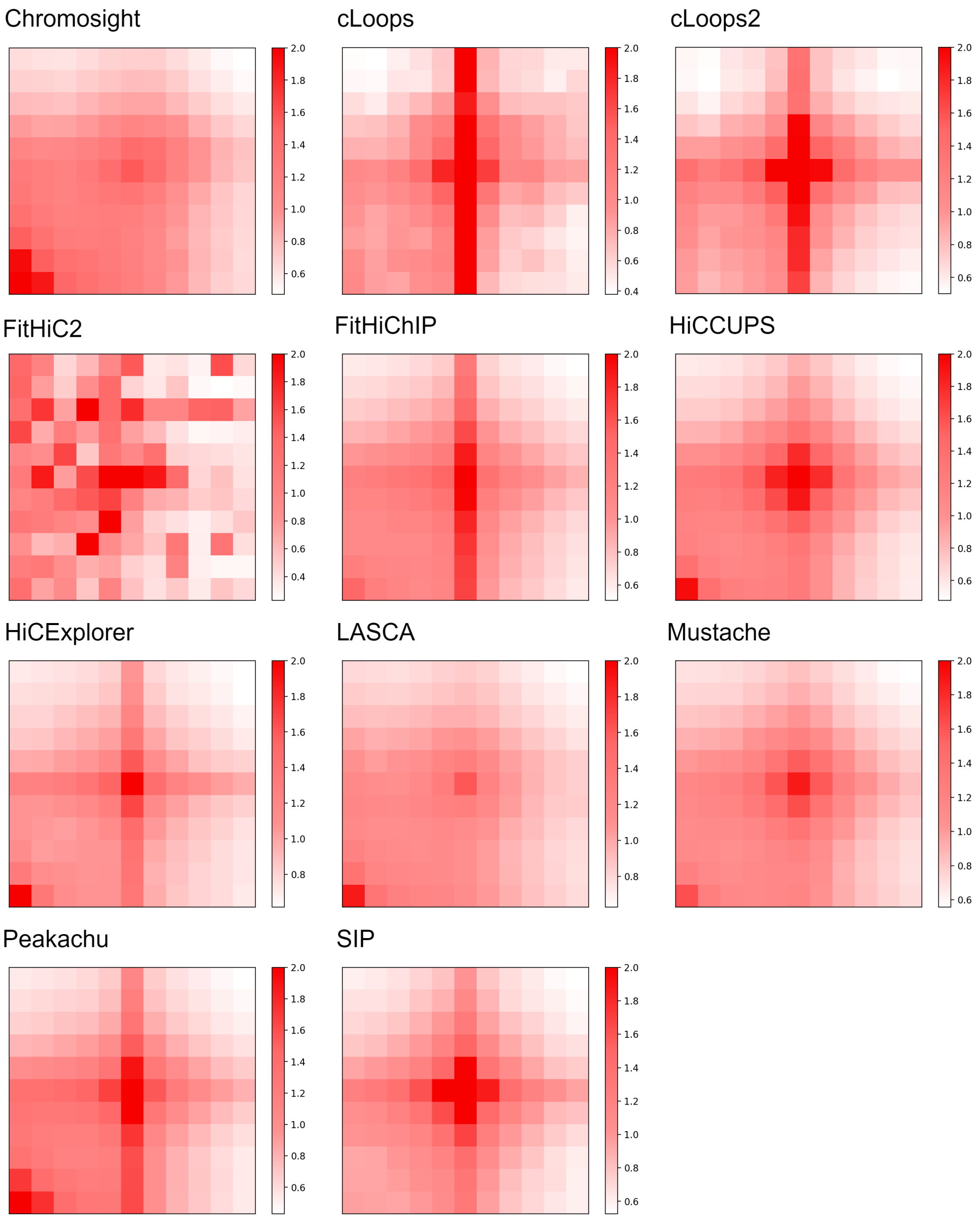

Figure S19 APA plot for chromosome 6 at 10KB using GM12878 primary data. cLoops, cLoops2, FitHiChIP, and Peakachu have strong enrichment vertically through the center. FitHiC2 shows highest enrichment in multiple focal points and LASCA produced lowest APA score. Chromosight, Peakachu, HiCCUPS, HiCExplorer, and LASCA show strong enrichment at the lower left corner of the center.

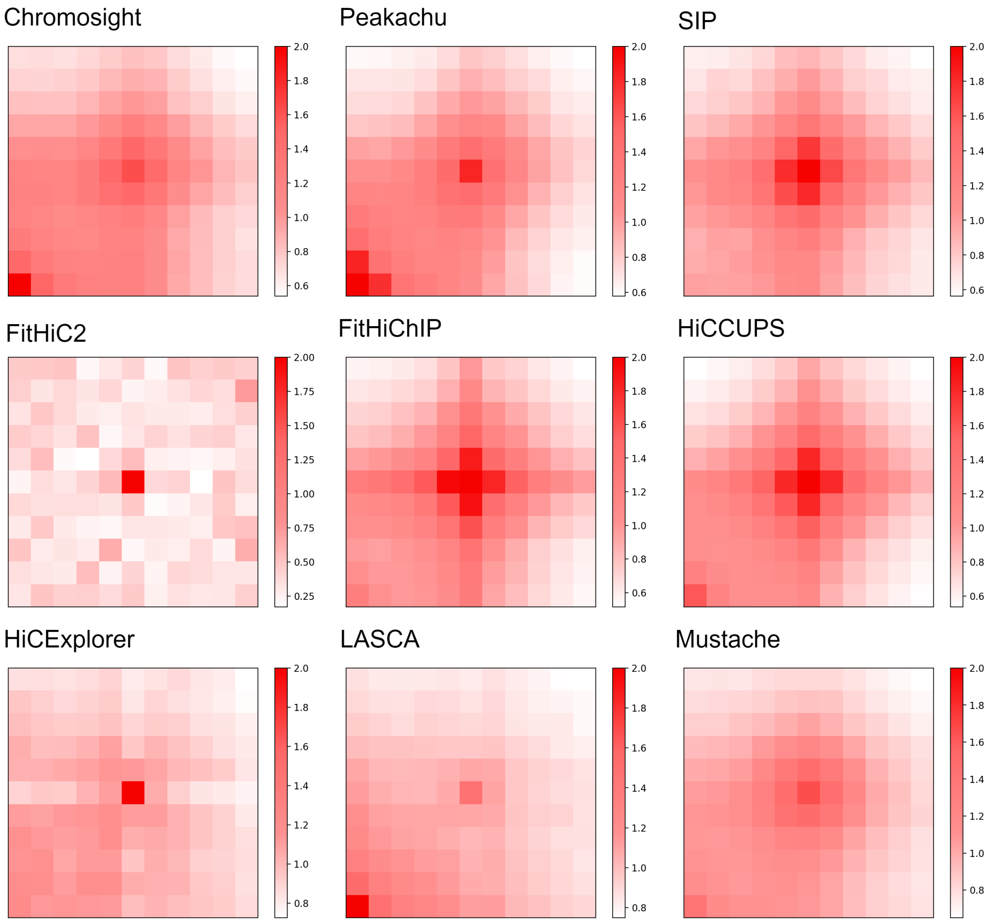

Figure S20 APA plot for chromosome 1 at 5KB using GM12878 KR normalized data. Though Chromosight, Peakachu, and LASCA have strong enrichment at the lower left corner, all the tools shows strong enrichment in the central focal point and score greater than 1.

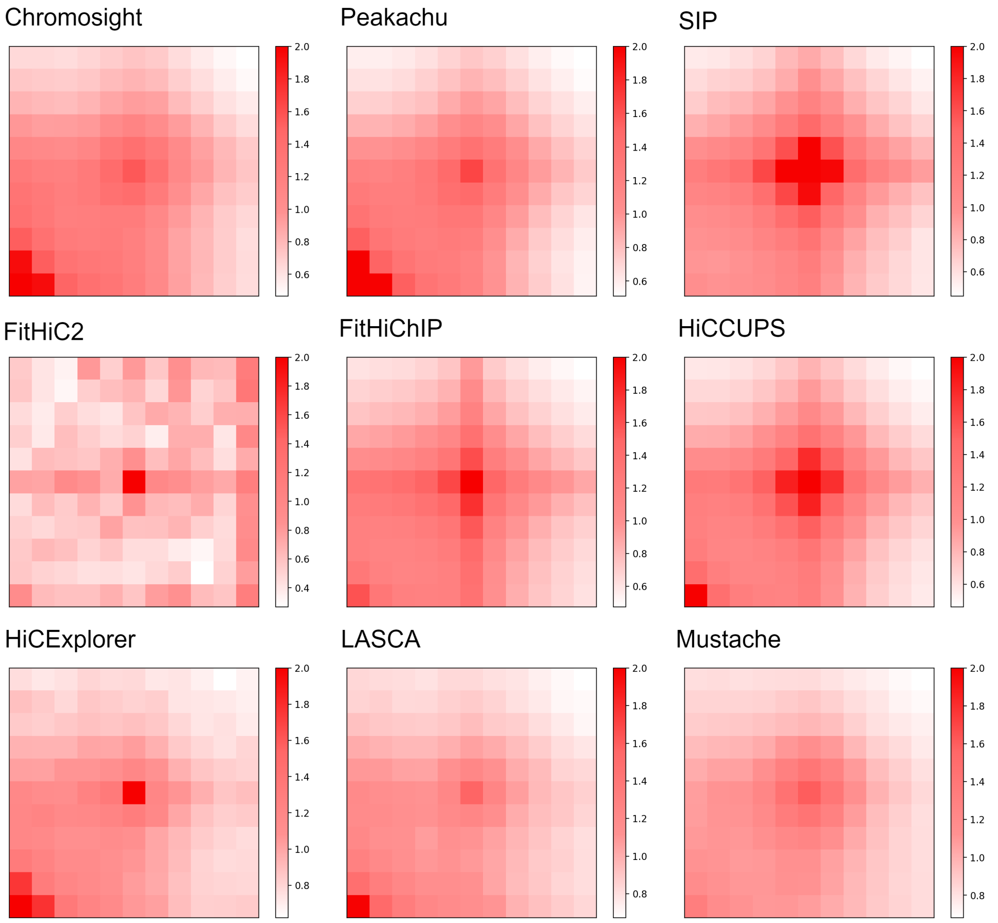

Figure S21 APA plot for chromosome 1 at 10KB using GM12878 KR normalized data. Here, all the tools have strong color in the center and dimmed symmetrically from the center except FitHiC2, and Peakachu and Chromosight score less than 1.

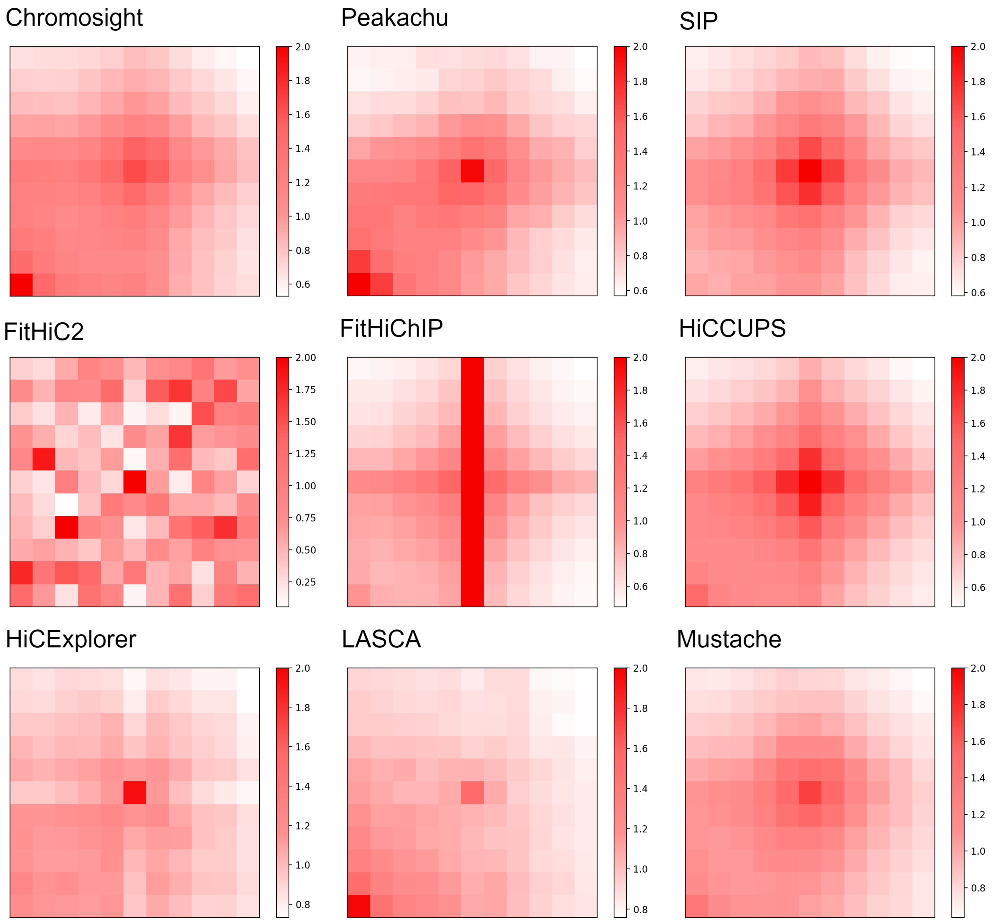

Figure S22 APA plot for chromosome 6 at 5KB using GM12878 KR normalized data. FitHiChIP (4.86) has a vertical strong color through the center and FitHiC2 shows asymmetric color with a high APA score.

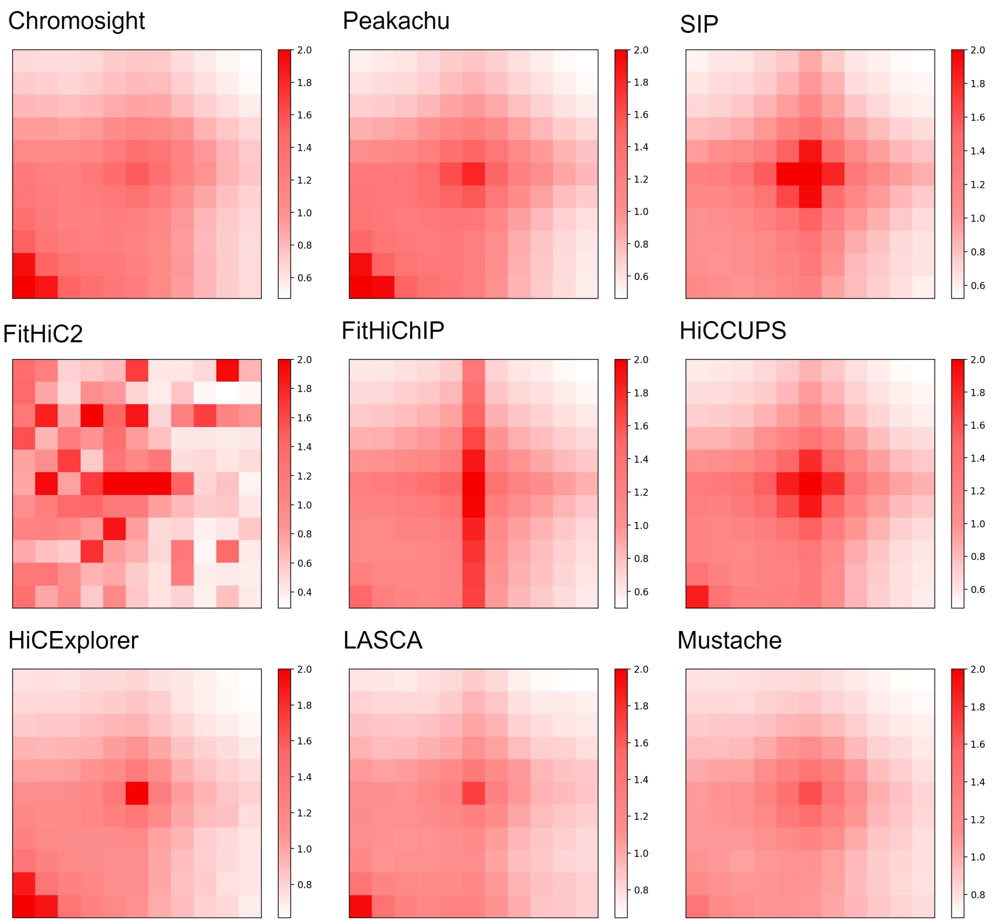

Figure S23 APA plot for chromosome 6 at 10KB using GM12878 KR normalized data. FitHiChIP shows the similar enrichment pattern as 5KB but a lower APA score. Chromosight, Peakachu, and HiCExplorer shows a similar color contrast at the lower left corner, and Chromosight scored less than 1.

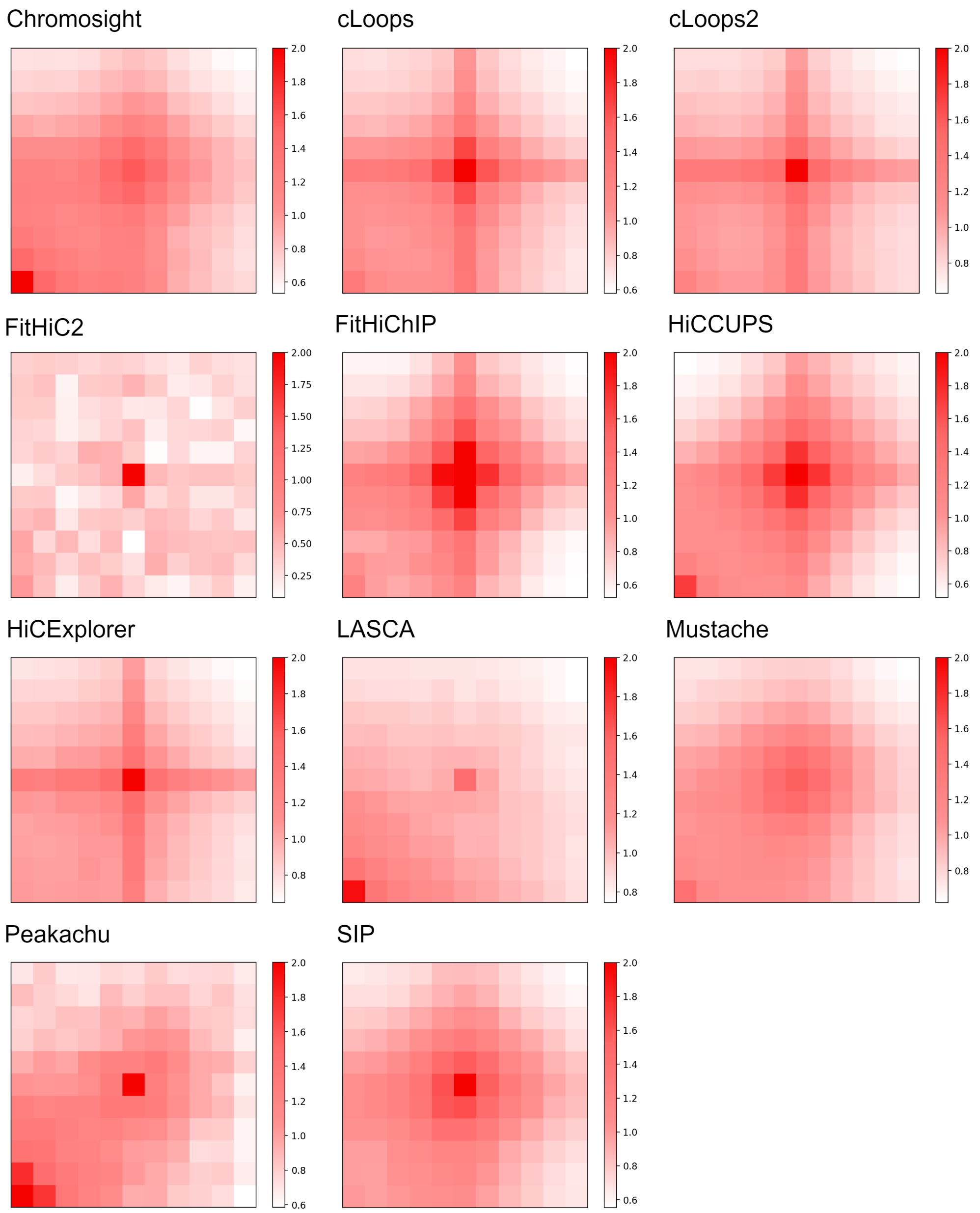

Figure S24 APA plot for chromosome 1 at 5KB using GM12878 replicate data. Peakachu, SIP, cLoops, cLoops2, HiCExplorer, FitHiChIP, HiCCUPS, FitHiC2, and LASCA have strong focal point in the center. Except FitHiC2 with the highest APA score, all of them have comparable enrichment around the center.

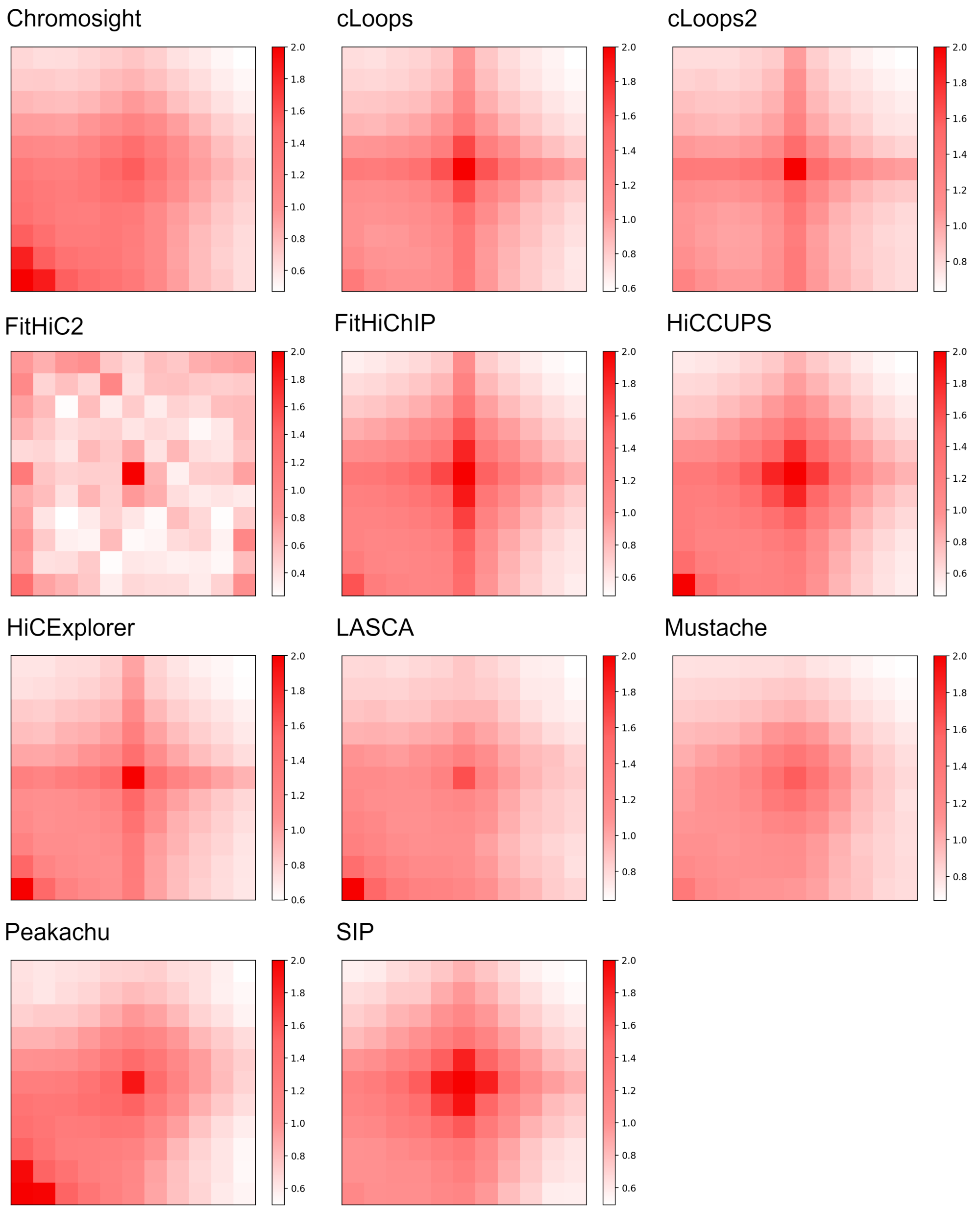

Figure S25 APA plot for chromosome 1 at 10KB using GM12878 replicate data. SIP and HiCCUPS show almost similar plot. Chromosight, HiCCUPS, HiCExplorer, LASCA, and Peakachu shows enrichment at the lower left corner with the center where Chromosight APA score is less than 1.

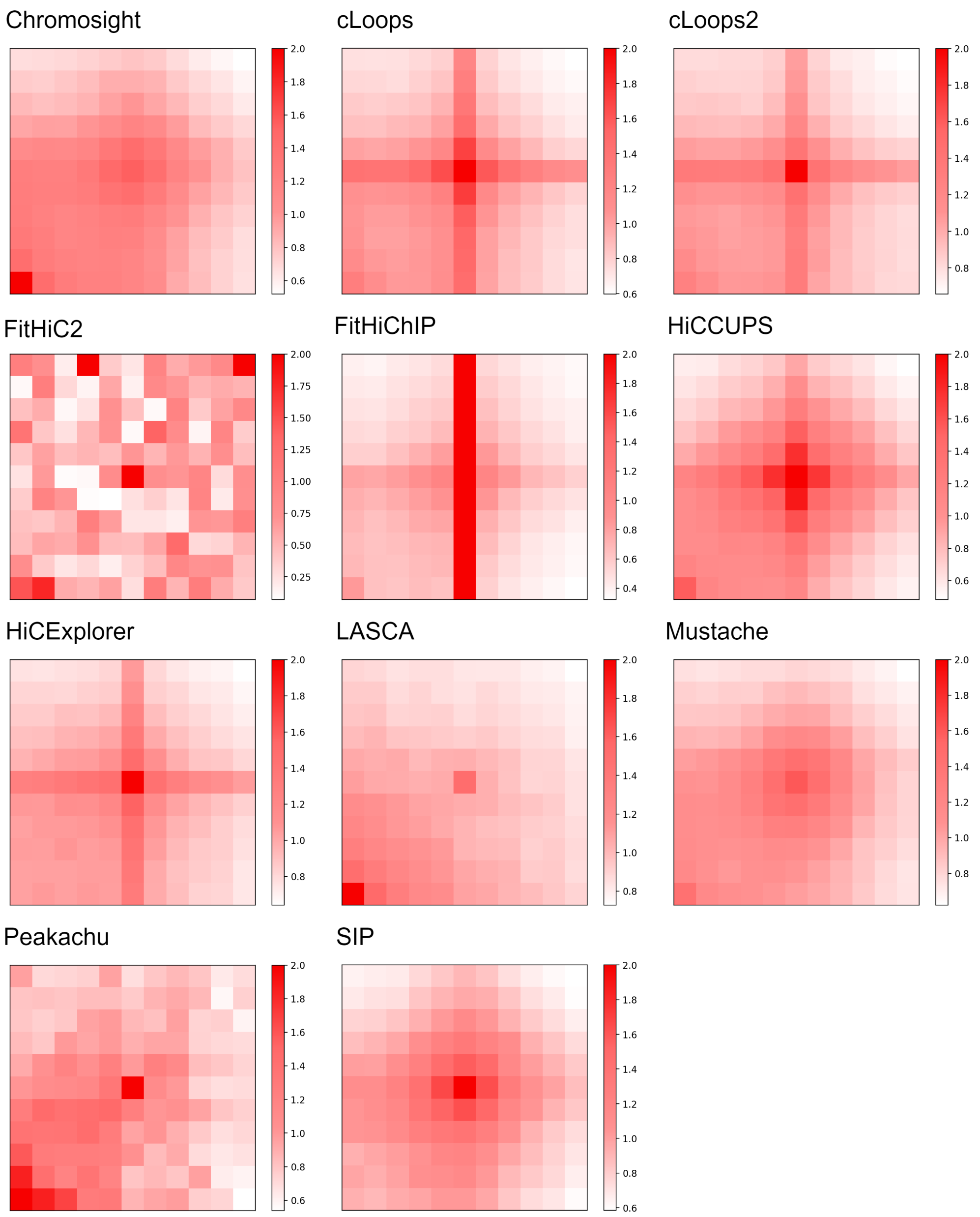

Figure S26 APA plot for chromosome 6 at 5KB using GM12878 replicate data. FitHiChIP has vertically strongest color through the center. FitHiC2 (67.8) and Peakachu (1.28) have almost similar pattern in their plots.

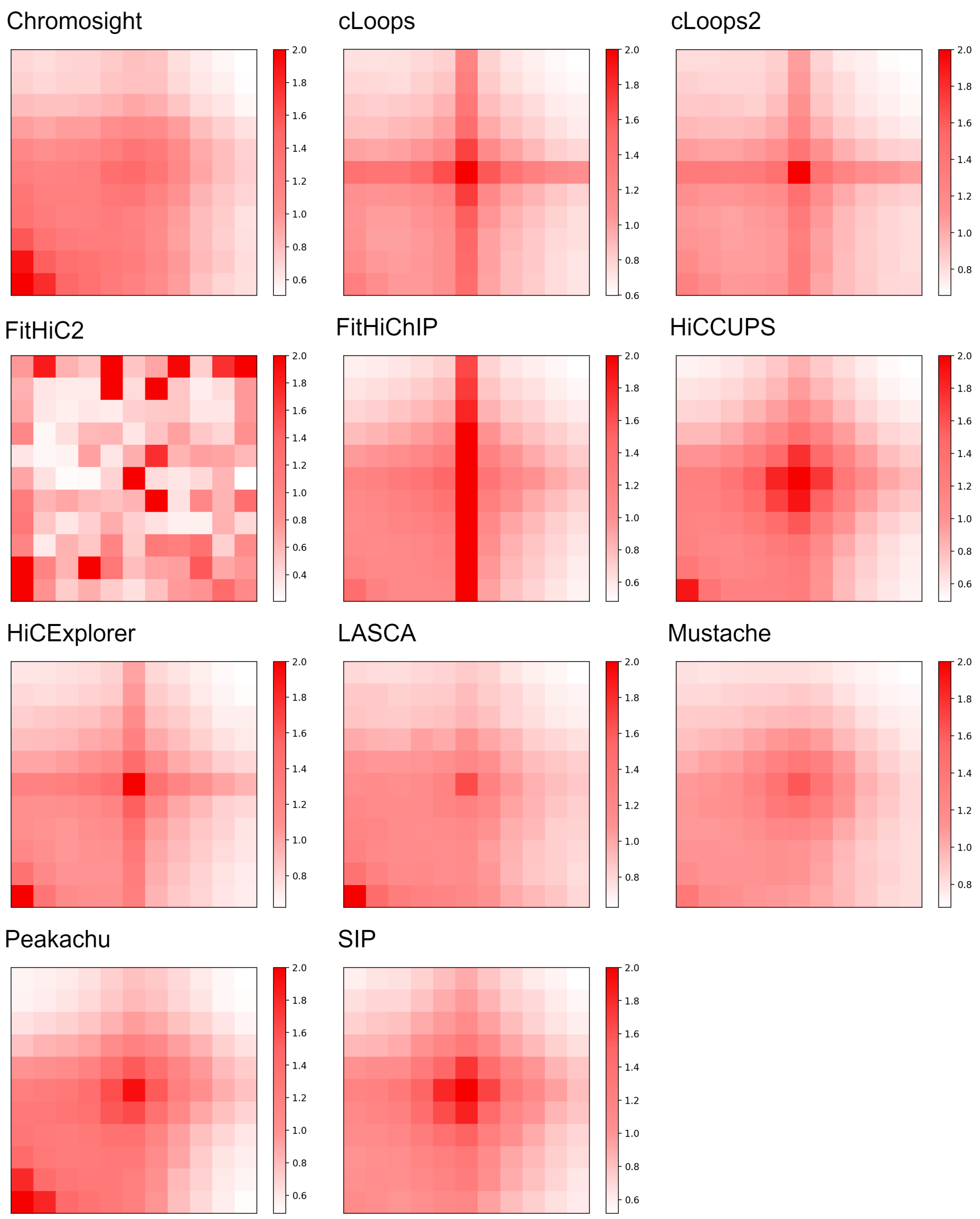

Figure S27 APA plot for chromosome 6 at 10KB using GM12878 replicate data. FitHiChIP continued the pattern from 5KB data and Peakachu has smoother heatmap compared to 5KB data. FitHiC2 scores 23.4 which is the highest and Chromosight (0.849) the lowest.

3.5 Recovery Analysis

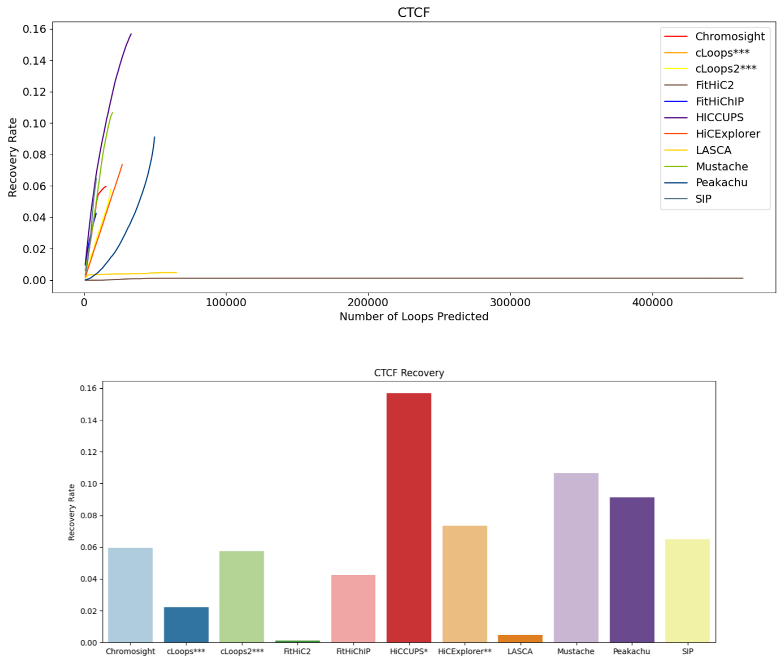

Figure S28 CTCF recovery rate at 5KB using primary GM12878.

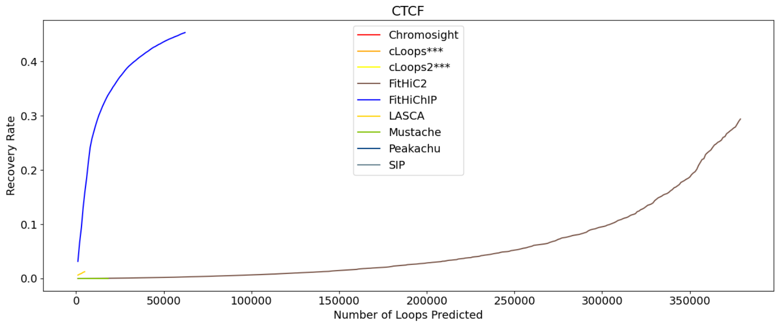

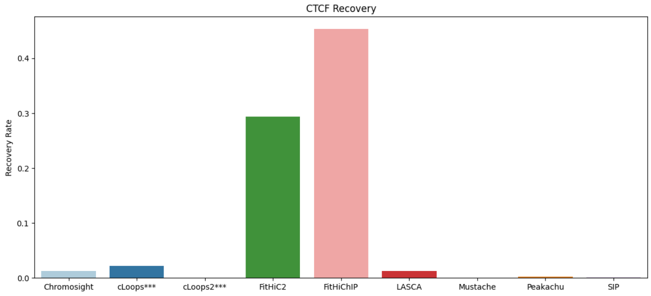

Figure S29 CTCF recovery rate at 100KB using primary GM12878.

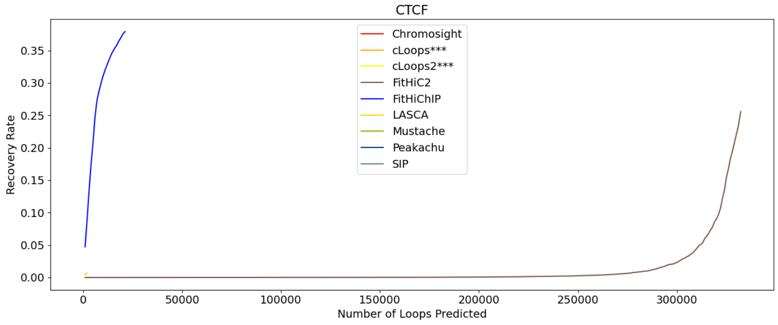

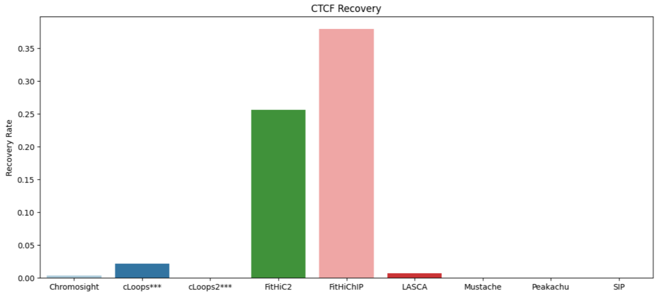

Figure S30 CTCF recovery rate at 250KB using primary GM12878.

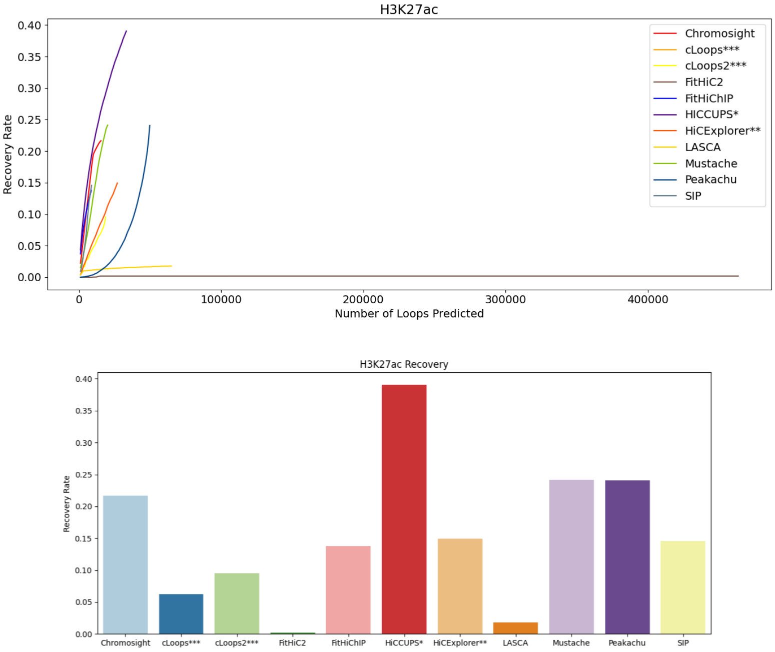

Figure S31 H3K27ac recovery rate at 5KB using primary GM12878.

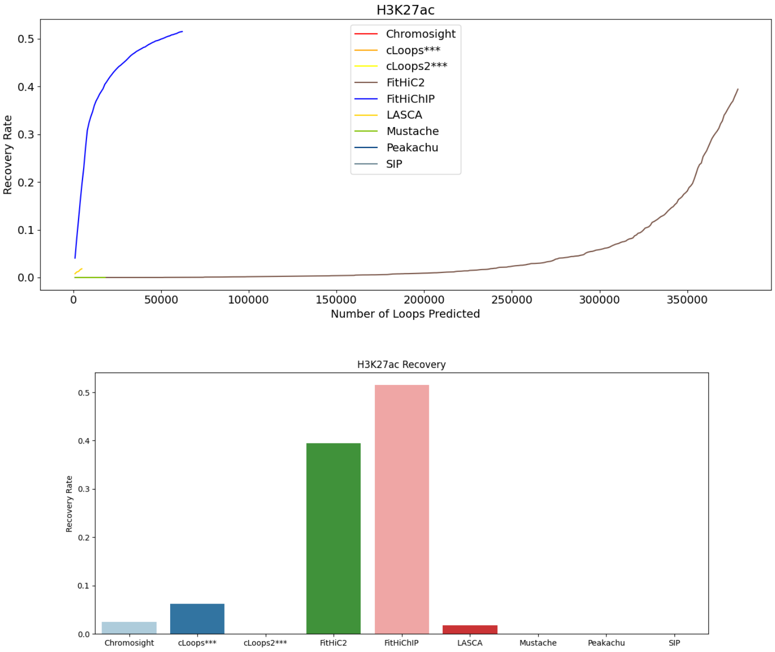

Figure S32 H3K27ac recovery rate at 100KB using primary GM12878.

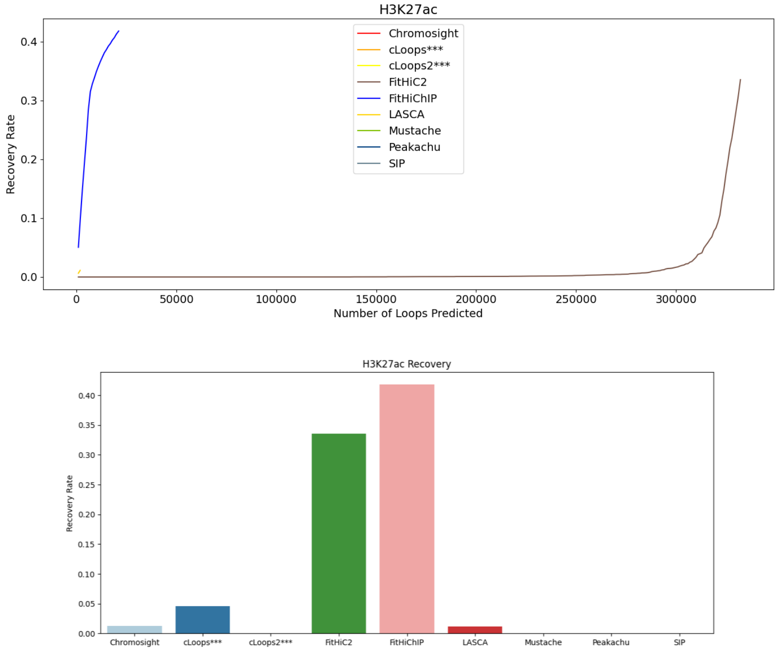

Figure S33 H3K27ac recovery rate at 250KB using primary GM12878.

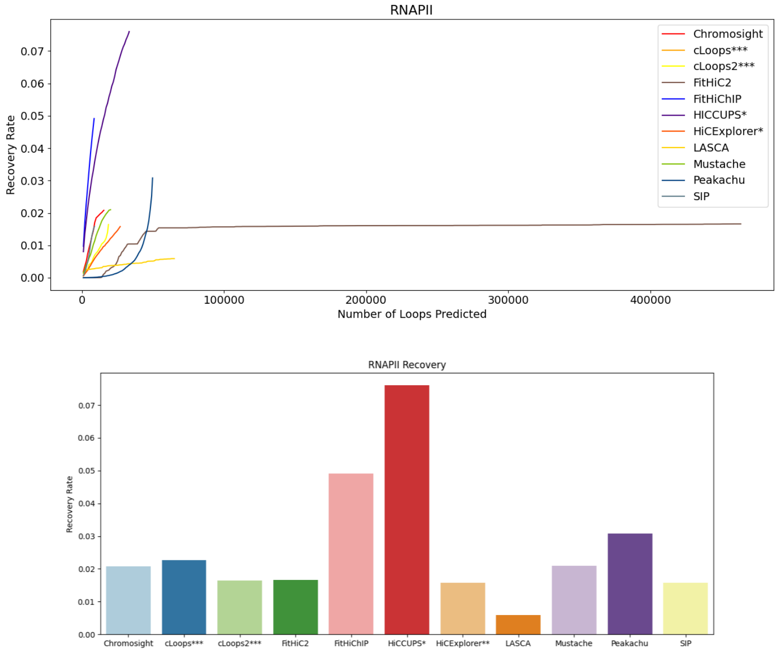

Figure S34 RNAPII recovery rate at 5KB using primary GM12878.

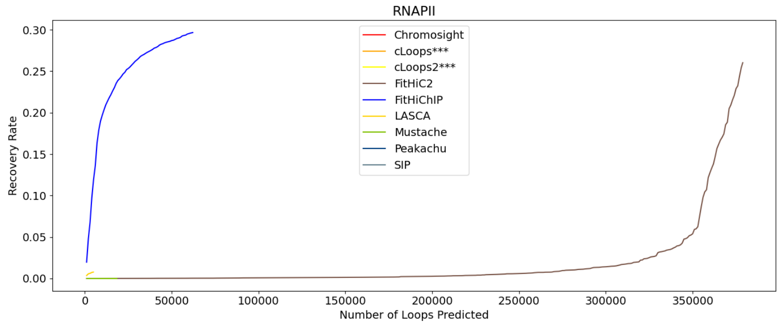

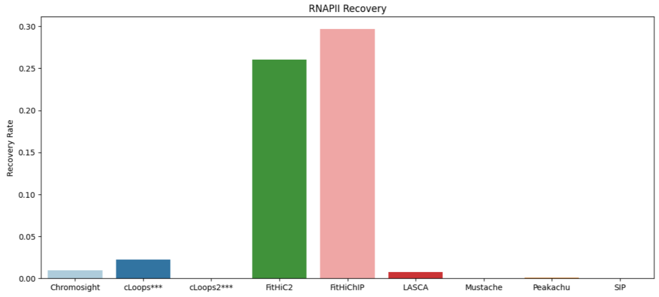

Figure S35 RNAPII recovery rate at 100KB using primary GM12878.

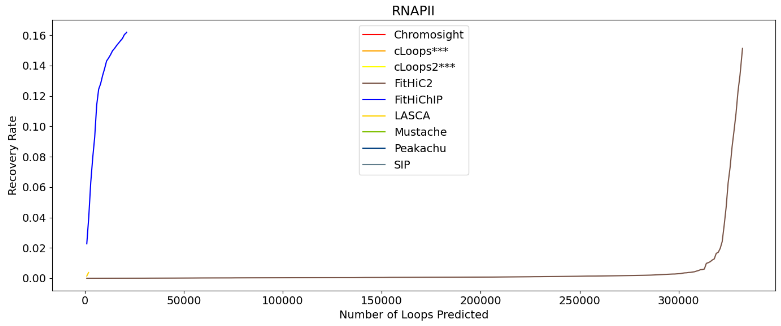

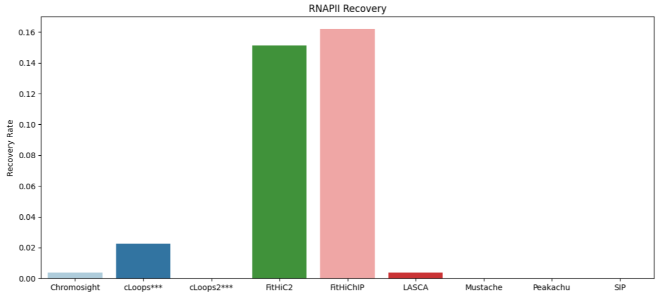

Figure S36 RNAPII recovery rate at 250KB using primary GM12878.

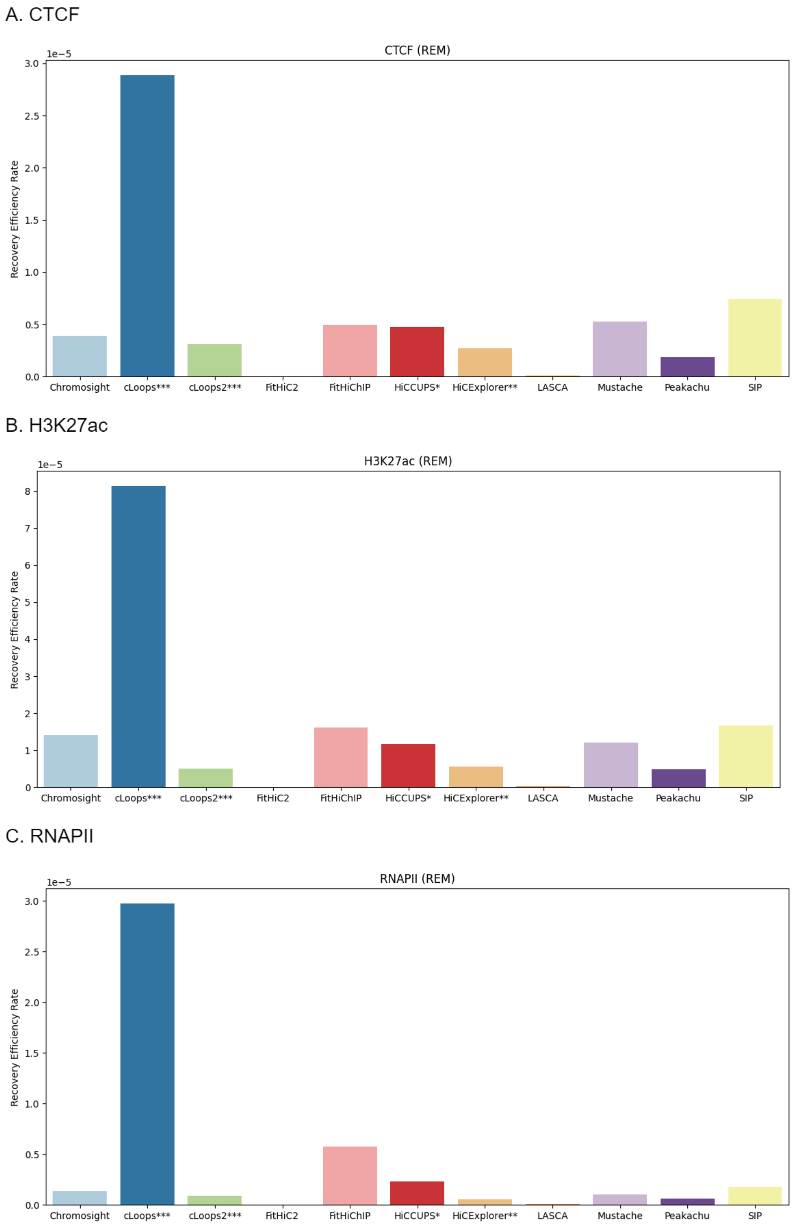

Figure S37 Recovery efficiency rate at 5KB using primary GM12878.

A. CTCF

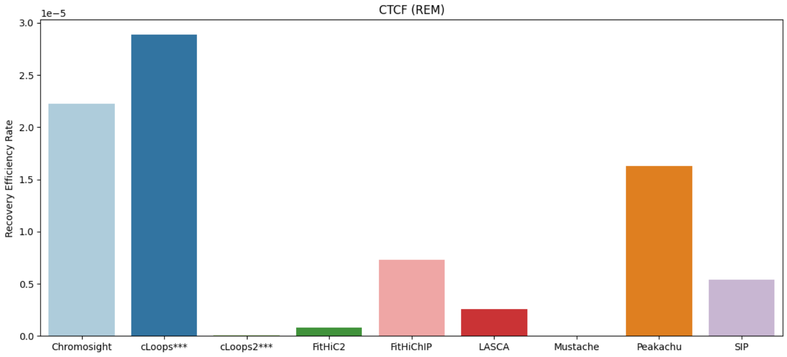

B. H3K27ac

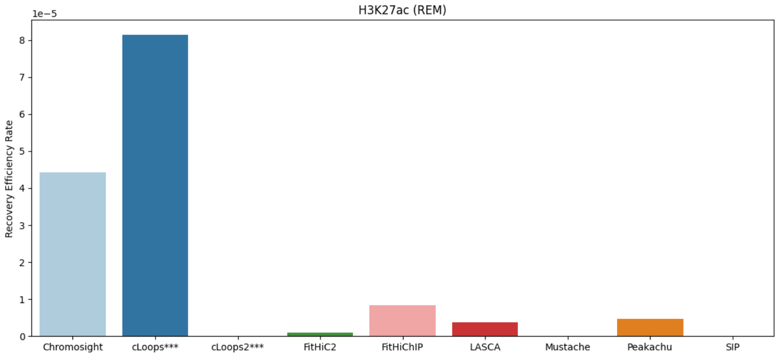

C. RNAPII

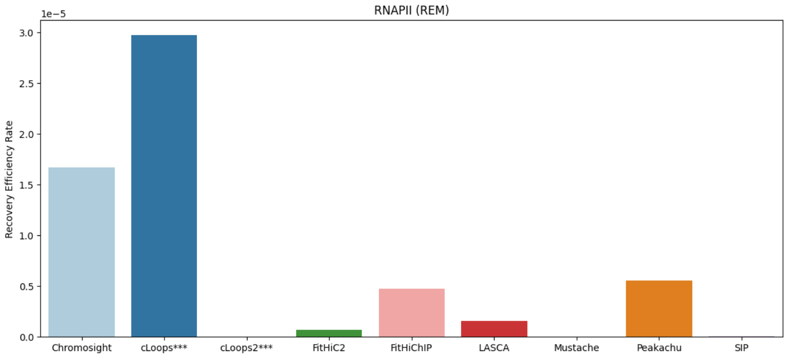

Figure S38 Recovery efficiency rate at 100KB using primary GM12878.

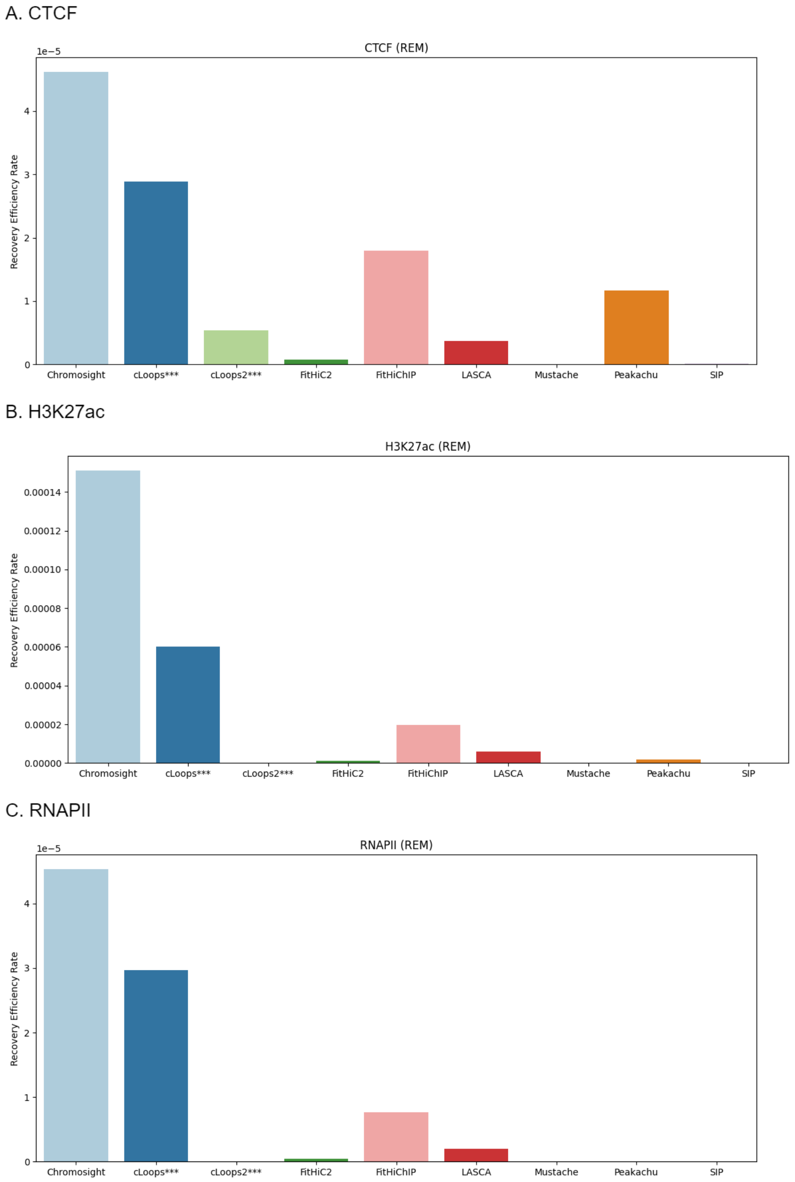

Figure S39 Recovery efficiency rate at 250KB using primary GM12878.

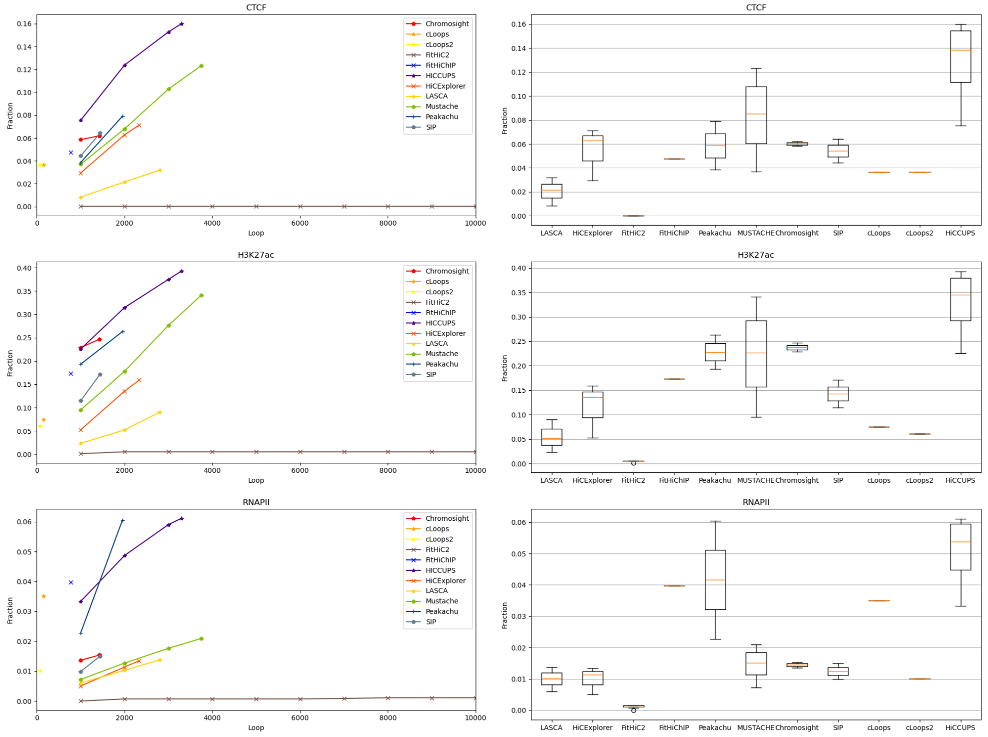

Figure S40 Recovery fraction of CTCF, H3K27ac, and RNAPII for chromosome 1 at 5KB using primary GM12878. FitHiC2 recovers a constant amount of CTCF, H3K27ac, and RNAPII and this is the lowest amount. Peakachu and HiCCUPs has the highest amount of recovery rate for RNAPII. For CTCT and H3K27ac, HiCCUPS holds the highest fraction of amount. On average, Mustache recovers a significant amount of loops. FitHiC2 shows an outlier data at the lower portion for H3K27ac3 and RNAPII.

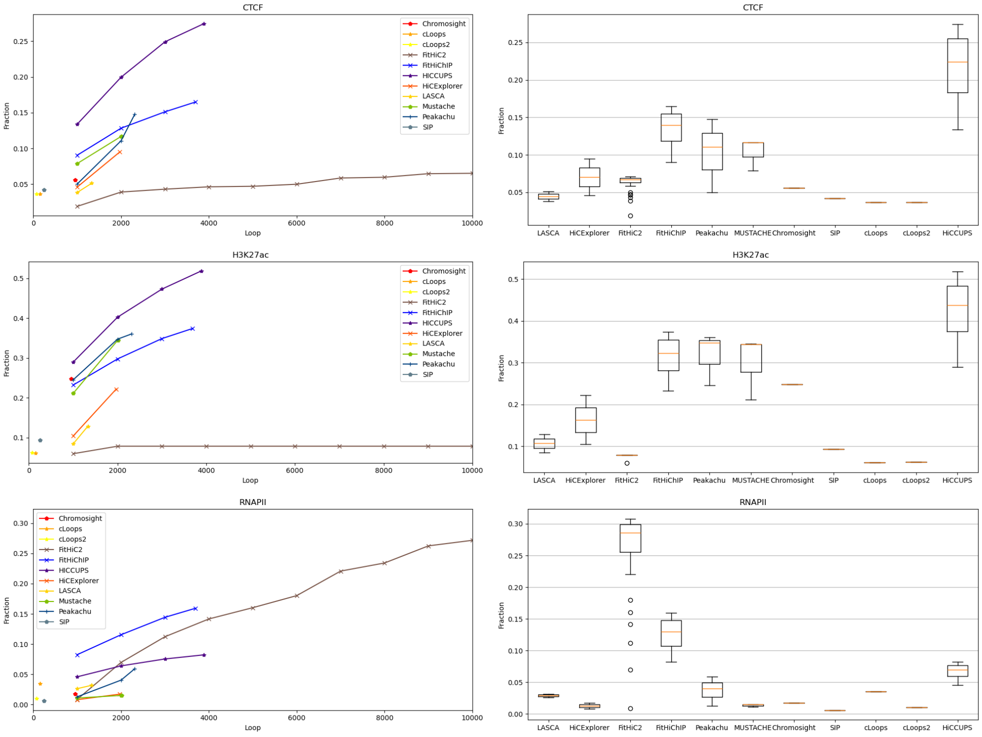

Figure S41 Recovery fraction of CTCF, H3K27ac, and RNAPII for chromosome 1 at 10KB using primary GM12878. Though FitHiC2 has a wide range of outliers, it shows an improvement in recovery rate for CTCF and RNAPII, and recovers the highest amount of RNAPII compared to other tools. HiCCUPS recovers most amount of CTCF and H3K27ac.

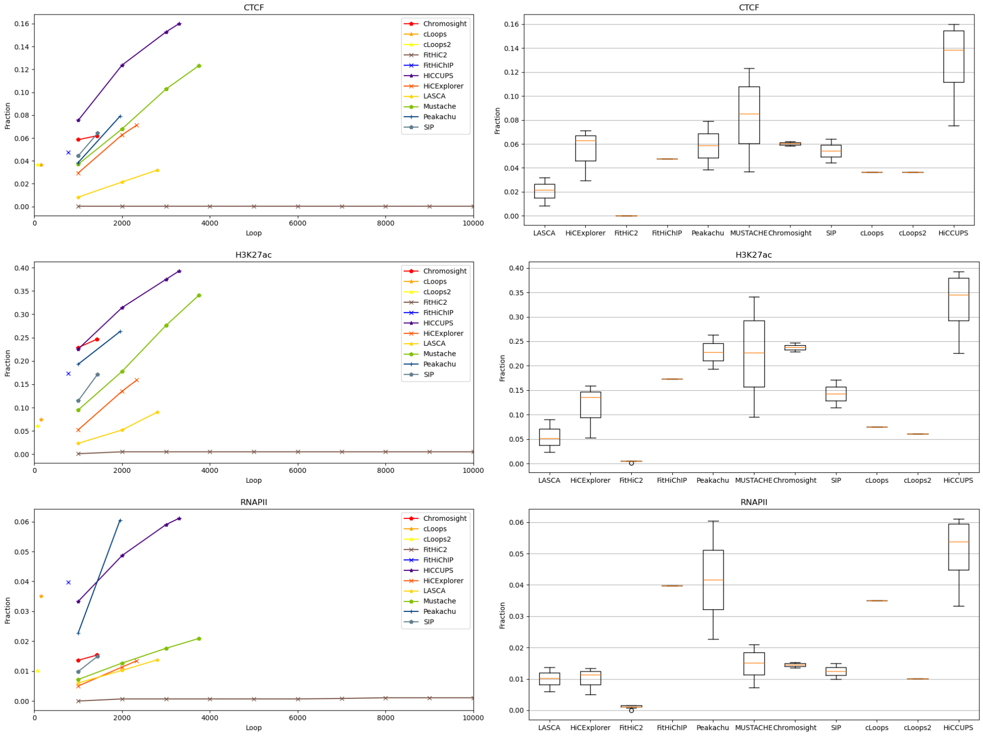

Figure S42 Recovery (CTCF, H3K27ac, and RNAPII) using chromosome 6 (primary GM12878) at 5KB with boxplot. HiCCUPS shows the highest fraction of recovery rate in three cases. FitHiC2 produces outliers for H3K27ac and RNAPII and has an improvement for CTCF. Mustache recovers a significant amount of fraction of H3K27ac, FitHiChIP shows a good fraction of recovery at CTCF and Peakachu recovers a good amount of fraction of RNAPII.

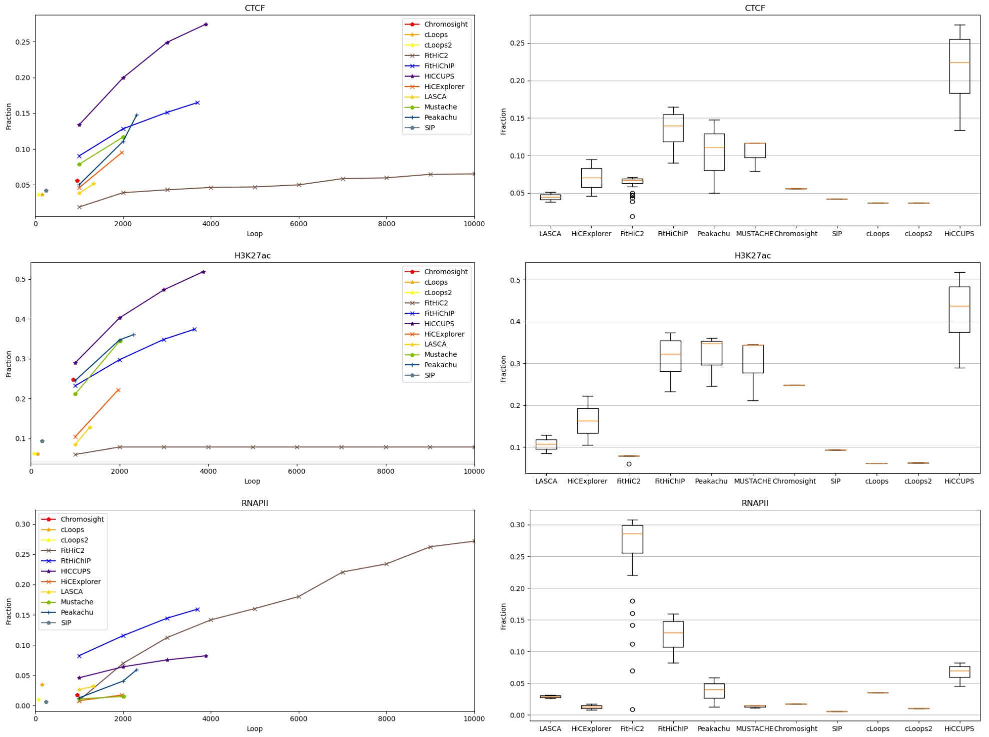

Figure S43 Recovery fraction of CTCF, H3K27ac, and RNAPII for chromosome 6 at 10KB using primary GM12878. HiCCUPS recovers shear amount of CTCF and H3K27ac compared to other tools. PFitHiC2 recovers highest fraction of RNAPII.

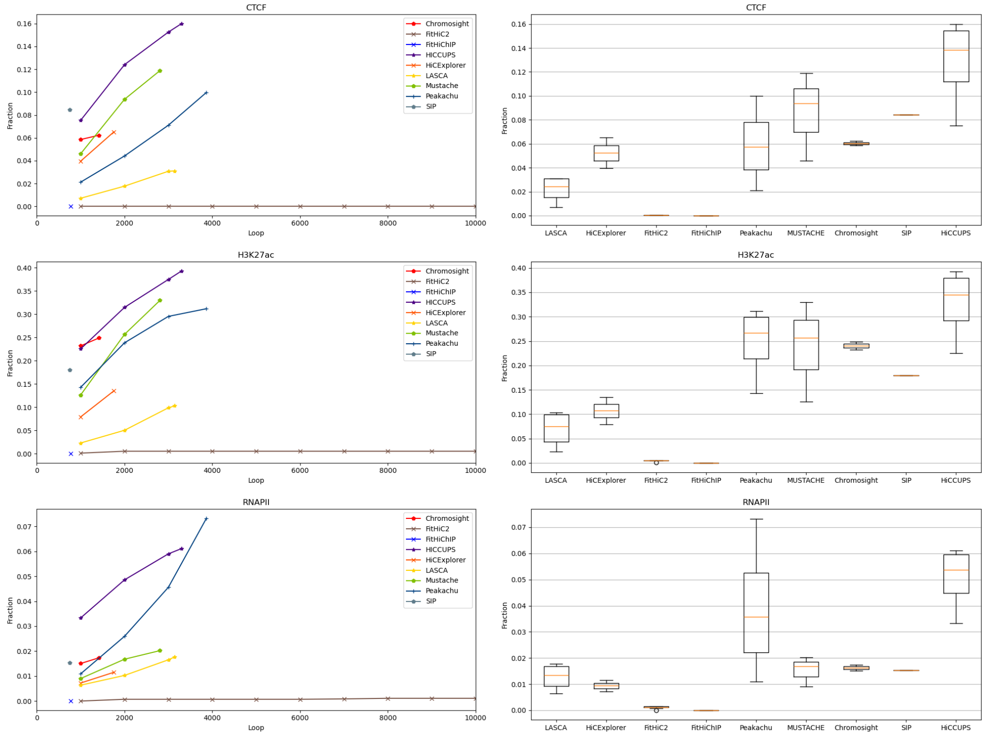

Figure S44 Recovery fraction of CTCF, H3K27ac, and RNAPII for chromosome 1 at 5KB using KR normalized GM12878. HiCCUPS recovers highest number of fraction CTCF and H3K27ac, and Peakachu recovers RNAPII. FitHiC2 remain constant which is almost near to 0. On average, Mustache recovers a good number of loops in these three category. FitHiC2 has outliers in H3K27ac and RNAPII.

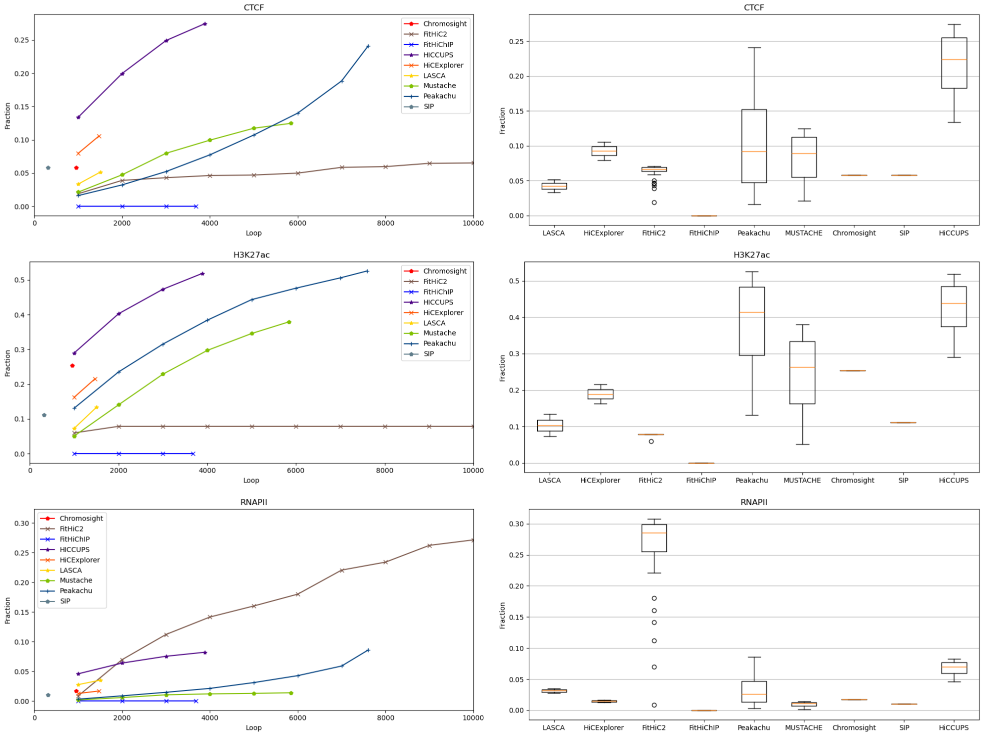

Figure S45 Recovery fraction of CTCF, H3K27ac, and RNAPII for chromosome 1 at 10KB using KR normalized GM12878. HiCCUPS and Peakachu recovers significant amount of CTCF and H3K27ac and Mustache recovers an average number of fraction. In contrast, RNAPII are mostly recovered by FitHiC2 with outliers in the three cases.

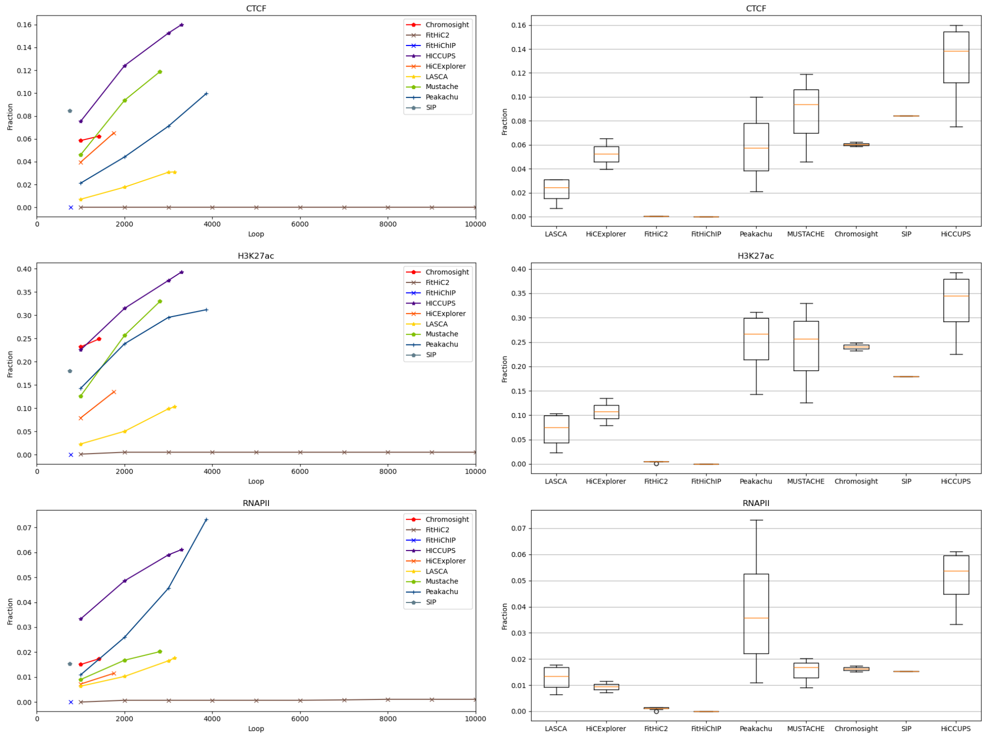

Figure S46 Recovery fraction of CTCF, H3K27ac, and RNAPII for chromosome 6 at 5KB using KR normalized GM12878. HiCCUPS and Mustache recovers shear amount of CTCF and H3K27ac compared to other tools. Peakachu and HiCCUPS recovers highest fraction of RNAPII. FitHiC2 shows a constant recovery rate with some outliers.

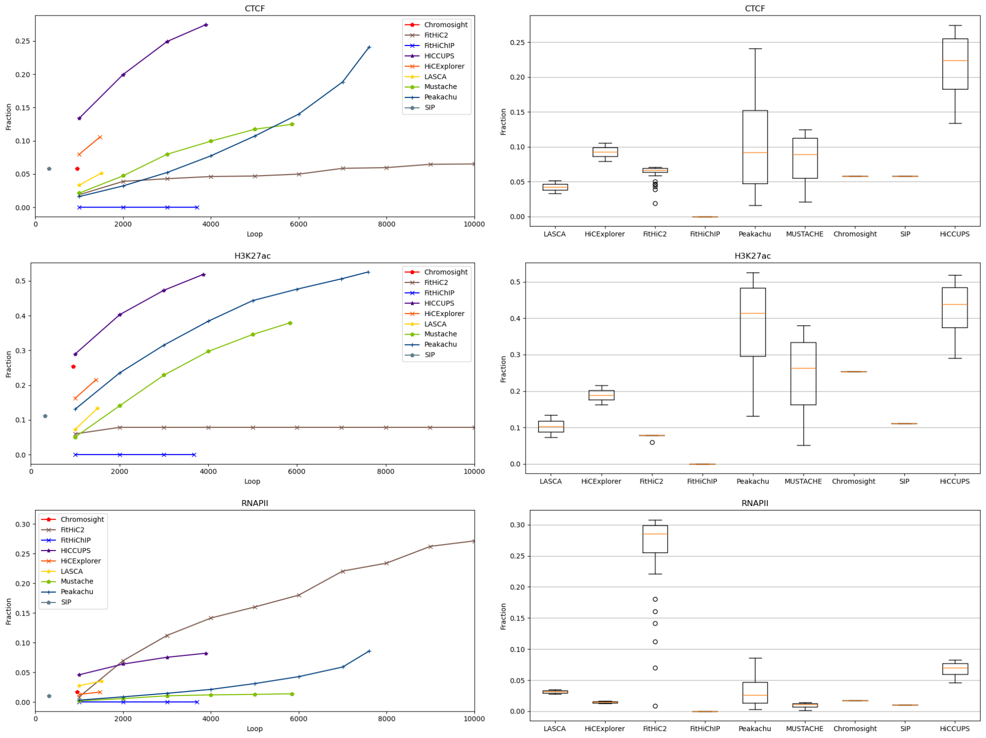

Figure S47 Recovery fraction of CTCF, H3K27ac, and RNAPII for chromosome 6 at 10KB using KR normalized GM12878. A large amount of CTCF and H3K27ac are recovered by HiCCUPS and Peakachu whereas Mustache recover an average number of loops among the other tools. FitHiChIP recovers near 0 loops. FitHiC2 recovers above 25% RNAPII and it has outliers in the three cases.

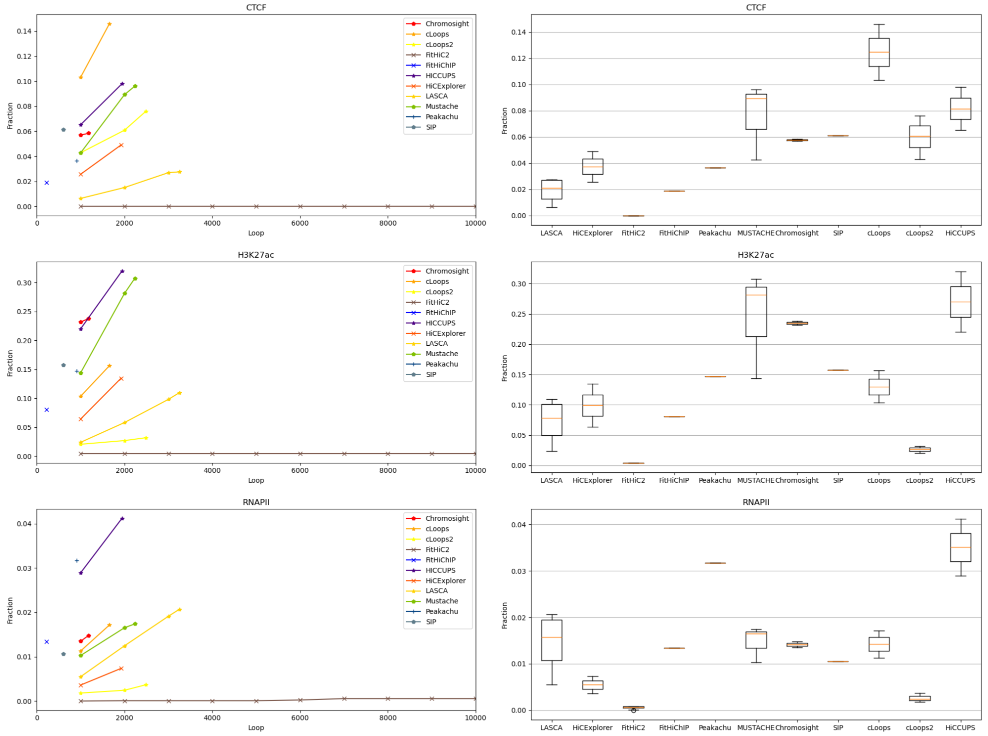

Figure S48 Recovery fraction of CTCF, H3K27ac, and RNAPII for chromosome 1 at 5KB using replicate GM12878. cLoops recovers a shear amount of CTCF, Mustache and HiCCUPS recovers near about 10% CTCF. Mustache and HiCCUPS recovers above 0.30 fraction of H3K27ac. Chromosight recovers near about 0.25 fraction of H3K27ac. RNAPII mostly rocovered by HiCCUPS. FitHiC2 shows a outlier point for RNAPII recovery.

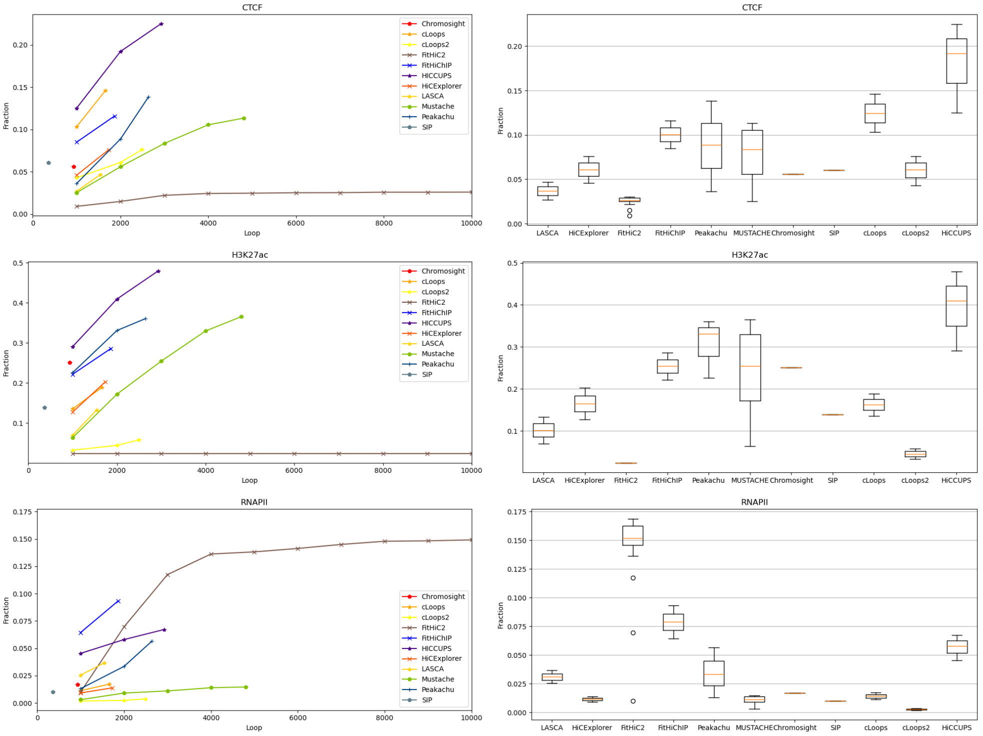

Figure S49 Recovery fraction of CTCF, H3K27ac, and RNAPII for chromosome 1 at 10KB using replicate GM12878. HiCCUPS and Peakachu recovers a large fractional amount of CTCF and H3K27ac and FitHiC2 recovers most RNAPII compared to other tools and has outlier points for CTCF and RNAPII recovery. Mustache holds the middle position for CTCF and H3K27ac, and HiCCUPS and FitHiChIP for RNAPII.

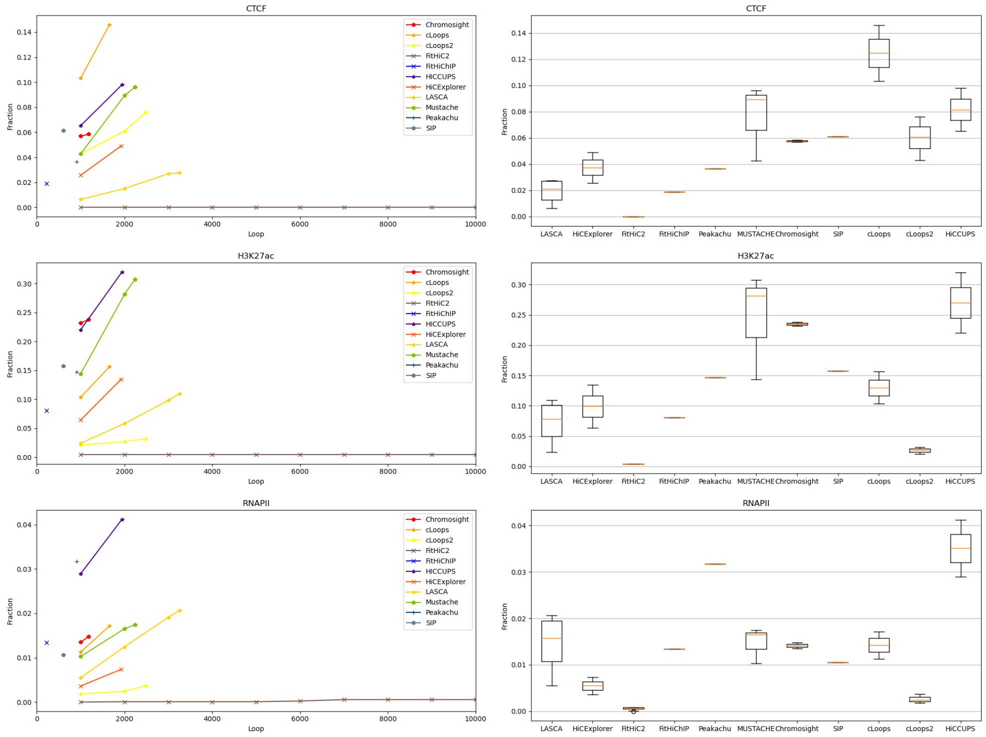

Figure S50 Recovery fraction of CTCF, H3K27ac, and RNAPII for chromosome 6 at 5KB using replicate GM12878. cLoops recovers above 0.14 fractional CTCF, HiCCUPS and Mustache recovers above 0.30 H3K27ac. HiCCUPS recovers above 0.04 fractional RNAPII. LASCA recovers average in terms of CTCF and RNAPII recovery. FitHiC2 shows an outlier point in RNAPII recovery.

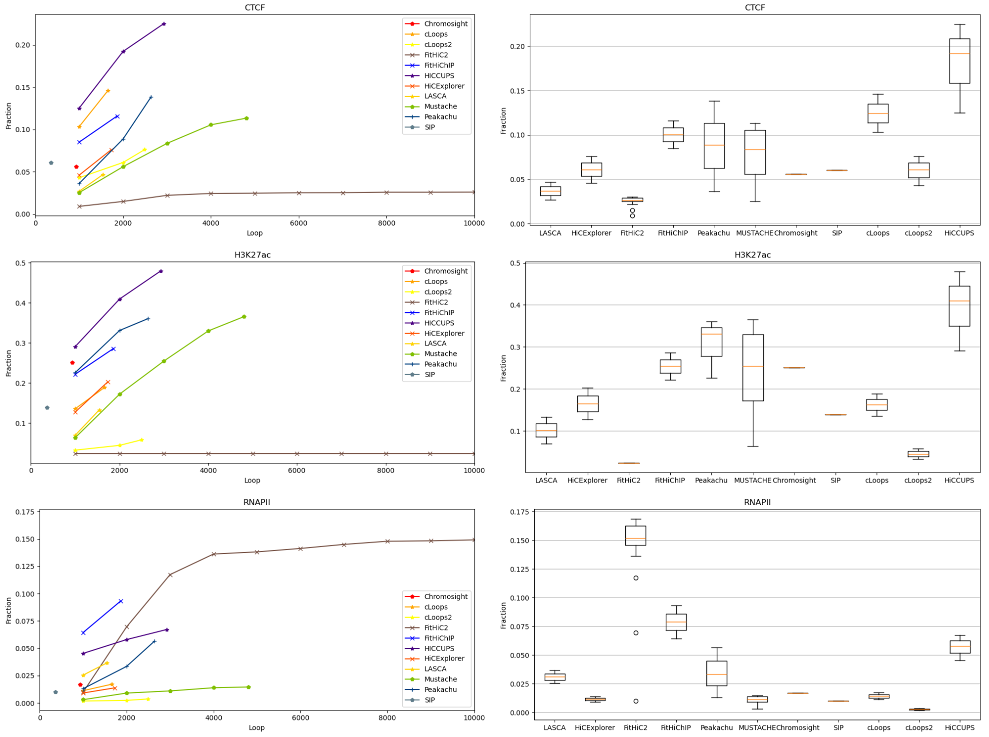

Figure S51 Recovery fraction of CTCF, H3K27ac, and RNAPII for chromosome 6 at 10KB using replicate GM12878. HiCCUPS recovers most of the CTCF and H3K27ac whereas FitHiC2 recovers RNAPII and has outlier points in CTCF and RNAPII recovery. cLoops2 recovers least fraction of H3K27ac and RNAPII.

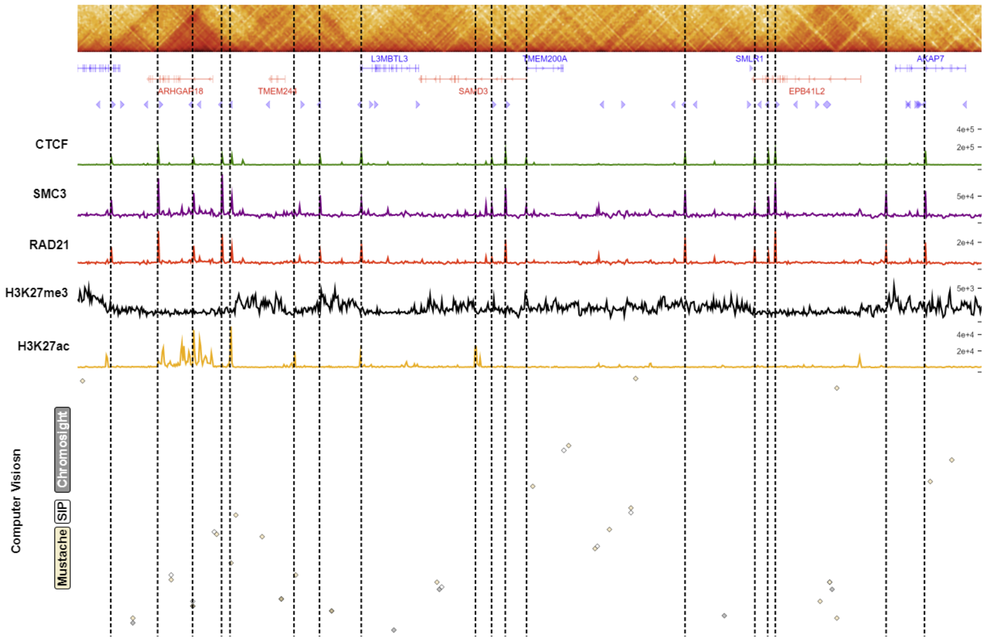

Figure S52 Comparison of computer vision based tools (Mustache, SIP and Chromosight) in a random region (129.7M-131.6M and 62.4M - 62.5M) for chromosome 6 using GM12878 cell line. We depicted gene annotation, CTCT motif orientation, ChIP signals for CTCF, SMC3, RAD21, H3K27me3 and H3K27ac below the contact map (Plotted using HiGlass). At the bottom, loops from three tools show their regions with biologically significant area.

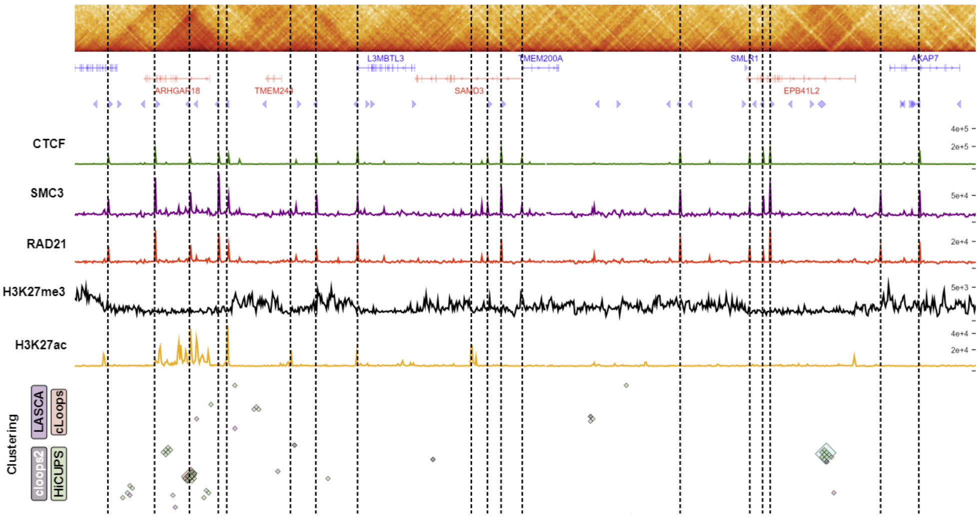

Figure S53 Comparison of clustering based tools (HiCCUPS, cLoops2, cLoops and LASCA) in a random region (129.7M-131.6M and 62.4M - 62.5M) for chromosome 6 using GM12878 cell line. We depicted gene annotation, CTCT motif orientation, ChIP signals for CTCF, SMC3, RAD21, H3K27me3 and H3K27ac below the contact map (Plotted using HiGlass). At the bottom, loops from HiCCUPS, cLoops2, cLoops and LASCA show their regions with biologically significant area.

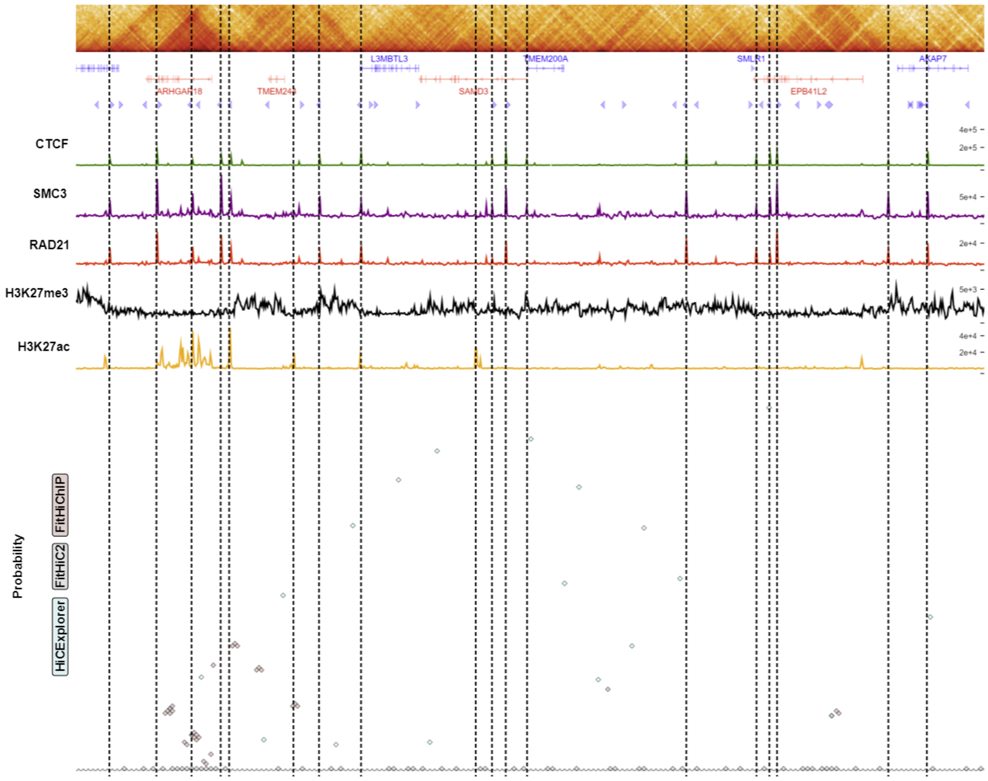

Figure S54 Comparison of probability based tools (HiCExplorer, FitHiC2, and FitHiChIP) in a random region (129.7M-131.6M and 62.4M - 62.5M) for chromosome 6 using GM12878 cell line. We depicted gene annotation, CTCT motif orientation, ChIP signals for CTCF, SMC3, RAD21, H3K27me3 and H3K27ac below the contact map (Plotted using HiGlass). At the bottom, loops from three tools show their regions with biologically significant area.

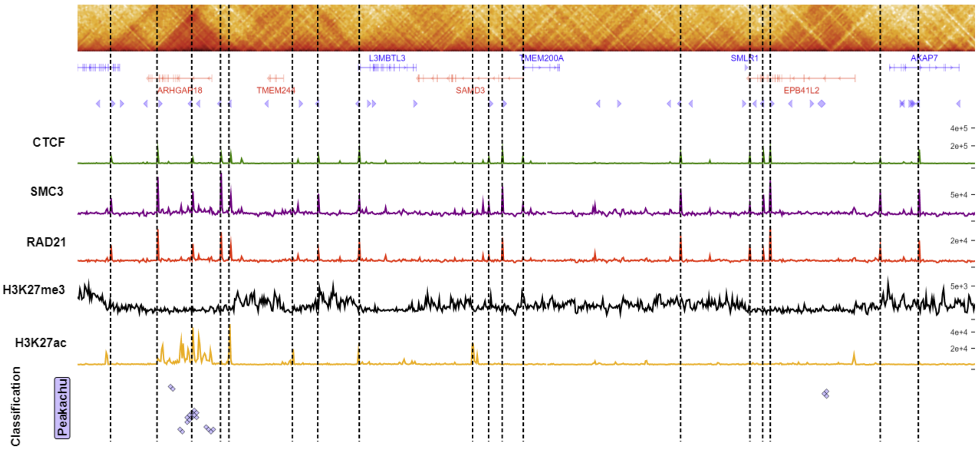

Figure S55 Comparison of classification based tools (Peakachu) in a random region (129.7M-131.6M and 62.4M - 62.5M) for chromosome 6 using GM12878 cell line. We depicted gene annotation, CTCT motif orientation, ChIP signals for CTCF, SMC3, RAD21, H3K27me3 and H3K27ac below the contact map (Plotted using HiGlass). At the bottom, loops from Peakachu shows their regions with biologically significant areas.

References

[1] Galan, S., Serra, F., Marti-Renom, M.A.: Identification of chromatin loops from hi-c interaction matrices by ctcf–ctcf topology classification. NAR genomics and bioinformatics **4**(1), 021 (2022)

[2] Grant, C.E., Bailey, T.L., Noble, W.S.: Fimo: scanning for occurrences of a given motif. Bioinformatics **27**(7), 1017–1018 (2011)

[3] Ma, W., Noble, W.S., Bailey, T.L.: Motif-based analysis of large nucleotide data sets using meme-chip. Nature protocols **9**(6), 1428–1450 (2014)

[4] Serra, F., Baù, D., Goodstadt, M., Castillo, D., Filion, G.J., Marti-Renom, M.A.: Automatic analysis and 3d-modelling of hi-c data using tadbit reveals structural features of the fly chromatin colors. PLoS computational biology **13**(7), 1005665 (2017)

[5] Rao, S.S., Huntley, M.H., Durand, N.C., Stamenova, E.K., Bochkov, I.D., Robinson, J.T., Sanborn, A.L., Machol, I., Omer, A.D., Lander, E.S., *et al.*: A 3d map of the human genome at kilobase resolution reveals principles of chromatin looping. Cell **159**(7), 1665–1680 (2014)

[6] Kohonen, T.: Adaptive, associative, and self-organizing functions in neural computing. Applied optics **26**(23), 4910–4918 (1987)

[7] Luzhin, A.V., Golov, A.K., Gavrilov, A.A., Velichko, A.K., Ulianov, S.V., Razin, S.V., Kantidze, O.L.: Lasca: loop and significant contact annotation pipeline. Scientific Reports **11**(1), 1–9 (2021)

[8] Cao, Y., Chen, Z., Chen, X., Ai, D., Chen, G., McDermott, J., Huang, Y., Guo, X., Han, J.-D.J.: Accurate loop calling for 3d genomic data with cloops. Bioinformatics **36**(3), 666–675 (2020)

[9] Cao, Y., Liu, S., Ren, G., Tang, Q., Zhao, K.: cloops2: a full-stack comprehensive analytical tool for chromatin interactions. Nucleic Acids Research **50**(1), 57–71 (2022)

[10] Wolff, J., Backofen, R., Grüning, B.: Loop detection using hi-c data with hicexplorer. Gigascience **11** (2022)

[11] Heinz, S., Benner, C., Spann, N., Bertolino, E., Lin, Y.C., Laslo, P., Cheng, J.X., Murre, C., Singh, H., Glass, C.K.: Simple combinations of lineage-determining transcription factors prime cis-regulatory elements required for macrophage and b cell identities. Molecular cell **38**(4), 576–589 (2010)

[12] Robinson, M.D., McCarthy, D.J., Smyth, G.K.: edger: a bioconductor package for differential expression analysis of digital gene expression data. bioinformatics

26(1), 139–140 (2010)

[13] McCarthy, D.J., Chen, Y., Smyth, G.K.: Differential expression analysis of multifactor rna-seq experiments with respect to biological variation. Nucleic acids research **40**(10), 4288–4297 (2012)

[14] Ay, F., Bailey, T.L., Noble, W.S.: Statistical confidence estimation for hi-c data reveals regulatory chromatin contacts. Genome research **24**(6), 999–1011 (2014)

[15] Kaul, A., Bhattacharyya, S., Ay, F.: Identifying statistically significant chromatin contacts from hi-c data with fithic2. Nature protocols **15**(3), 991–1012 (2020)

[16] Lagler, T.M., Abnousi, A., Hu, M., Yang, Y., Li, Y.: Hic-act: improved detection of chromatin interactions from hi-c data via aggregated cauchy test. The American Journal of Human Genetics **108**(2), 257–268 (2021)

[17] Liu, Y., Xie, J.: Cauchy combination test: a powerful test with analytic p-value calculation under arbitrary dependency structures. Journal of the American Statistical Association **115**(529), 393–402 (2020)

[18] Yang, T., Zhang, F., Yardımcı, G.G., Song, F., Hardison, R.C., Noble, W.S., Yue, F., Li, Q.: Hicrep: assessing the reproducibility of hi-c data using a stratum-adjusted correlation coefficient. Genome research **27**(11), 1939–1949 (2017)

[19] Bhattacharyya, S., Chandra, V., Vijayanand, P., Ay, F.: Identification of significant chromatin contacts from hichip data by fithichip. Nature communications **10**(1), 4221 (2019)

[20] Benjamini, Y., Hochberg, Y.: Controlling the false discovery rate: a practical and powerful approach to multiple testing. Journal of the Royal statistical society: series B (Methodological) **57**(1), 289–300 (1995)

[21] Imakaev, M., Fudenberg, G., McCord, R.P., Naumova, N., Goloborodko, A., Lajoie, B.R., Dekker, J., Mirny, L.A.: Iterative correction of hi-c data reveals hallmarks of chromosome organization. Nature methods **9**(10), 999–1003 (2012)

[22] Mifsud, B., Martincorena, I., Darbo, E., Sugar, R., Schoenfelder, S., Fraser, P., Luscombe, N.M.: Gothic, a probabilistic model to resolve complex biases and to identify real interactions in hi-c data. PloS one **12**(4), 0174744 (2017)

[23] Carty, M., Zamparo, L., Sahin, M., González, A., Pelossof, R., Elemento, O., Leslie, C.S.: An integrated model for detecting significant chromatin interactions from high-resolution hi-c data. Nature communications **8**(1), 15454 (2017)

[24] Zeileis, A., Kleiber, C., Jackman, S.: Regression models for count data in r. Journal of statistical software **27**(8), 1–25 (2008)

[25] Beaumont, M.A., Zhang, W., Balding, D.J.: Approximate bayesian computation

in population genetics. Genetics **162**(4), 2025–2035 (2002)

[26] Osuntoki, I.G., Harrison, A., Dai, H., Bao, Y., Zabet, N.R.: Ziphic: a novel bayesian framework to identify enriched interactions and experimental biases in hi-c data. Bioinformatics **38**(14), 3523–3531 (2022)

[27] Wu, F.-Y.: The potts model. Reviews of modern physics **54**(1), 235 (1982)

[28] Wang, X., Xu, J., Zhang, B., Hou, Y., Song, F., Lyu, H., Yue, F.: Genome-wide detection of enhancer-hijacking events from chromatin interaction data in rearranged genomes. Nature methods **18**(6), 661–668 (2021)

[29] Wang, S., Lee, S., Chu, C., Jain, D., Kerpedjiev, P., Nelson, G.M., Walsh, J.M., Alver, B.H., Park, P.J.: Hint: a computational method for detecting copy number variations and translocations from hi-c data. Genome biology **21**, 1–15 (2020)

[30] Chakraborty, A., Ay, F.: Identification of copy number variations and translocations in cancer cells from hi-c data. Bioinformatics **34**(2), 338–345 (2018)

[31] Salameh, T.J., Wang, X., Song, F., Zhang, B., Wright, S.M., Khunsriraksakul, C., Ruan, Y., Yue, F.: A supervised learning framework for chromatin loop detection in genome-wide contact maps. Nature communications **11**(1), 3428 (2020)

[32] Xu, Z., Zhang, G., Jin, F., Chen, M., Furey, T.S., Sullivan, P.F., Qin, Z., Hu, M., Li, Y.: A hidden markov random field-based bayesian method for the detection of long-range chromosomal interactions in hi-c data. Bioinformatics **32**(5), 650–656 (2016)

[33] Kindermann, R., Snell, J.L.: Markov Random Fields and Their Applications vol. 1. American Mathematical Society, ??? (1980)

[34] Besag, J., Green, P., Higdon, D., Mengersen, K.: Bayesian computation and stochastic systems. Statistical science, 3–41 (1995)

[35] Gelman, A., Carlin, J.B., Stern, H.S., Rubin, D.B.: Bayesian data analysis chapman & hall. CRC Texts in Statistical Science **136** (2004)

[36] Crowley, C., Yang, Y., Qiu, Y., Hu, B., Abnousi, A., Lipiński, J., Plewczyński, D., Wu, D., Won, H., Ren, B., *et al.*: Firecaller: Detecting frequently interacting regions from hi-c data. Computational and structural biotechnology journal **19**, 355–362 (2021)

[37] Schmitt, A.D., Hu, M., Jung, I., Xu, Z., Qiu, Y., Tan, C.L., Li, Y., Lin, S., Lin, Y., Barr, C.L., *et al.*: A compendium of chromatin contact maps reveals spatially active regions in the human genome. Cell reports **17**(8), 2042–2059 (2016)

[38] Whyte, W.A., Orlando, D.A., Hnisz, D., Abraham, B.J., Lin, C.Y., Kagey, M.H., Rahl, P.B., Lee, T.I., Young, R.A.: Master transcription factors and mediator

establish super-enhancers at key cell identity genes. Cell **153**(2), 307–319 (2013)

[39] Roayaei Ardakany, A., Gezer, H.T., Lonardi, S., Ay, F.: Mustache: multi-scale detection of chromatin loops from hi-c and micro-c maps using scale-space representation. Genome biology **21**, 1–17 (2020)

[40] Rowley, M.J., Poulet, A., Nichols, M.H., Bixler, B.J., Sanborn, A.L., Brouhard, E.A., Hermetz, K., Linsenbaum, H., Csankovszki, G., Aiden, E.L., *et al.*: Analysis of hi-c data using sip effectively identifies loops in organisms from c. elegans to mammals. Genome research **30**(3), 447–458 (2020)

[41] Durand, N.C., Shamim, M.S., Machol, I., Rao, S.S., Huntley, M.H., Lander, E.S., Aiden, E.L.: Juicer provides a one-click system for analyzing loop-resolution hi-c experiments. Cell systems **3**(1), 95–98 (2016)

[42] Schneider, C.A., Rasband, W.S., Eliceiri, K.W.: Nih image to imagej: 25 years of image analysis. Nature methods **9**(7), 671–675 (2012)

[43] Matthey-Doret, C., Baudry, L., Breuer, A., Montagne, R., Guiglielmoni, N., Scolari, V., Jean, E., Campeas, A., Chanut, P.H., Oriol, E., *et al.*: Computer vision for pattern detection in chromosome contact maps. Nature communications **11**(1), 5795 (2020)

[44] Pearce, D.J.: An improved algorithm for finding the strongly connected components of a directed graph. Victoria University, Wellington, NZ, Tech. Rep (2005)

[45] Zhang, S., Plummer, D., Lu, L., Cui, J., Xu, W., Wang, M., Liu, X., Prabhakar, N., Shrinet, J., Srinivasan, D., *et al.*: Deeploop robustly maps chromatin interactions from sparse allele-resolved or single-cell hi-c data at kilobase resolution. Nature genetics **54**(7), 1013–1025 (2022)

[46] Flyamer, I.M., Illingworth, R.S., Bickmore, W.A.: Coolpup. py: versatile pile-up analysis of hi-c data. Bioinformatics **36**(10), 2980–2985 (2020)
